# Mycobactericidal activity of bedaquiline plus rifabutin or rifampin in *ex vivo* whole blood cultures of healthy volunteers: A randomized controlled trial

**DOI:** 10.1371/journal.pone.0196756

**Published:** 2018-05-02

**Authors:** Robert S. Wallis, Caryn E. Good, Mary A. O’Riordan, Jeffrey L. Blumer, Michael R. Jacobs, J. McLeod Griffiss, Amanda Healan, Robert A. Salata

**Affiliations:** 1 Aurum Institute, Johannesburg, South Africa; 2 School of Medicine, Case Western Reserve University and University Hospitals Cleveland Medical Center, Cleveland, Ohio, United States of America; 3 ACT for TB/HIV, Johannesburg, South Africa; 4 University of Toledo, Toledo, Ohio, United States of America; 5 Clinical Research Management, Inc, Hinckley, Ohio, United States of America; Johns Hopkins University, UNITED STATES

## Abstract

**Background:**

Bedaquiline, an antimycobacterial agent approved for drug-resistant tuberculosis, is metabolized by CYP3A4, an hepatic enzyme strongly induced by rifampin, an essential part of drug-sensitive tuberculosis treatment. We examined the pharmacokinetic interactions of bedaquiline plus either rifampin or rifabutin in 33 healthy volunteers. This sub-study of that trial examined the mycobactericidal activity of these drugs against intracellular *Mycobacterium tuberculosis* using *ex vivo* whole blood culture.

**Methods:**

Subjects were randomly assigned to receive two single 400 mg doses of bedaquiline, alone, and, after a 4 week washout period, in combination with steady-state daily dosing of either rifabutin 300 mg or rifampin 600 mg. Blood samples were collected prior to dosing and at multiple time points subsequently, to measure plasma drug concentrations and bactericidal activity in *ex vivo M tuberculosis*-infected whole blood cultures (WBA).

**Results:**

Single oral doses of bedaquiline produced readily detectable WBA *ex vivo*, reaching a maximal effect of -0.28 log/day, with negative values indicating bacterial killing. Plasma concentrations of 355 ng/ml were sufficient for intracellular mycobacteriostasis. Combined dosing with rifampin or rifabutin produced maximal effects of -0.91 and -0.79 log/d, respectively. However, the activity of the rifabutin combination was sustained throughout the dosing interval, thereby producing a greater cumulative or total effect. At low drug concentrations, rifabutin plus bedaquiline yielded greater mycobactericidal activity than the sum of their separate effects. Neither drug metabolites nor cellular drug accumulation could account for this observation.

**Conclusions:**

The combination of rifabutin plus bedaquiline produces sustained intracellular mycobactericidal activity that is greater than the sum of their individual effects. Further studies of the treatment-shortening potential of this combination are warranted.

## Introduction

Despite considerable public health efforts during the past two decades, tuberculosis remains a global medical emergency, causing an estimated 10 million cases and 1.7 million deaths annually [1]. Important unmet medical needs exist for nearly all forms of *Mycobacterium tuberculosis* infections, including those susceptible to first-line TB drugs (DS-TB).

Bedaquiline (TMC207, Sirturo) is a diarylquinoline inhibitor of mycobacterial ATP synthetase [2]. It was approved by the US Food and Drug Administration (FDA) in 2012 for the treatment of multi-drug resistant (MDR) TB, based on improved rates of sputum culture conversion [3]. Bedaquiline has substantial promise to shorten treatment of drug-susceptible TB, based on *in vitro* and animal studies [4, 5]. However, bedaquiline is metabolized by the hepatic cytochrome P450 enzyme CYP3A4, which is strongly induced by both rifampin (a key component of standard TB treatment) and rifapentine (a candidate treatment-shortening agent) [6, 7]. These findings, plus unexpected safety concerns arising during long-term follow-up of the first bedaquiline-treated MDR-TB cohort [3], have delayed clinical trials of bedaquiline combined with first line drugs in the treatment of drug-susceptible TB.

A recently reported study examined the pharmacokinetics (PK) of two single 400 mg doses of bedaquiline administered one month apart to healthy volunteers, with the second dose being given in combination with daily dosing of either rifampin 600 mg or rifabutin 300 mg [8]. The long interval between doses was intended to permit partial elimination of bedaquiline and its M2 metabolite, both of which have long terminal half-lives [6]. The main findings of the study were that rifampin reduced bedaquiline exposure by 44%, whereas rifabutin had minimal effect. The present sub-study of that trial examined the intracellular mycobactericidal activity of bedaquiline alone, and with either rifamycin, using *ex vivo* whole blood culture. The objective was to examine the suitability of the combination of rifabutin plus bedaquiline in new TB regimens, and to help inform its treatment-shortening potential.

## Methods

### Trial design

**Fig 1:**
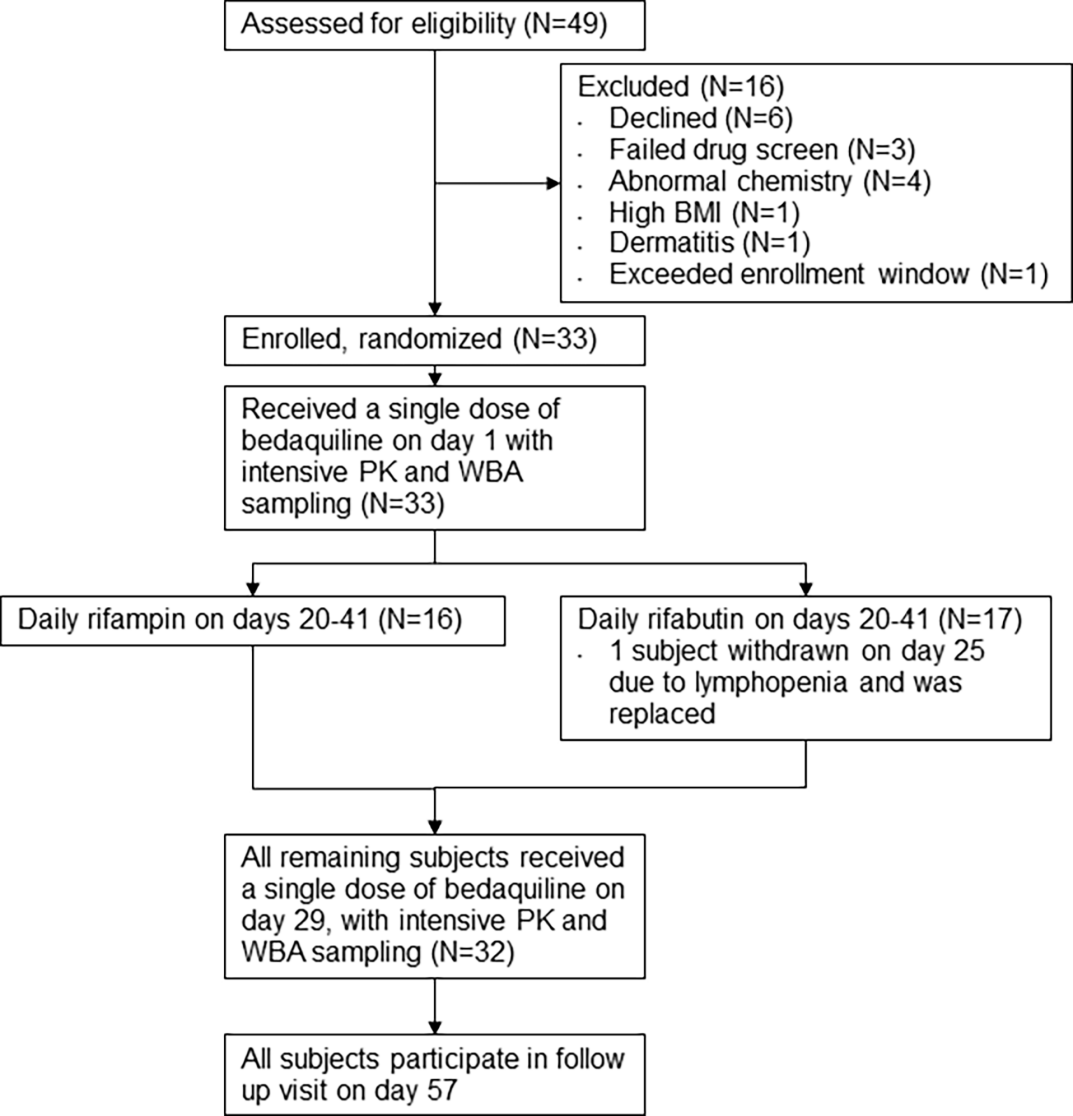
Study flow diagram.

Subjects were 33 healthy adults with normal or negative routine blood or urine tests for hematology, chemistry, coagulation, drug and alcohol use and pregnancy, and normal electrocardiograms. All subjects provided written informed consent to participate in the trial. The study protocol was reviewed by and received ethical approval from the Case Western Reserve University Institutional Review Board. The study was registered as trial NCT01341184 at clinicaltrials.gov. The trial design is illustrated in Fig 1. Following written informed consent and review of screening tests, subjects meeting enrollment criteria were randomly assigned to rifabutin or rifampin. All subjects received oral bedaquiline 400 mg on days 1 and 29, and either oral rifampin 600 mg, or rifabutin 300 mg, on days 20–41. Administration of all study drugs was directly observed. Subjects were admitted overnight for intensive PK and whole blood mycobactericidal activity (WBA) sampling on days 1 and 29. The clinical protocol is provided as a supplemental file. Clinical findings of the study are reported in reference [8].

### PK analysis

Bedaquiline was provided by Tibotec (Janssen). Sigma-Aldrich (St. Louis, MO) was the supplier for rifabutin (99% pure). Rifampicin, 98.4% pure, rifampicin-d4, and rifabutin-d7, 98.5% pure, were purchased from TLC PharmaChem (Ontario, Canada). Detection and analysis of bedaquiline, rifabutin and rifampin were performed using validated LC/MS/MS assays that were developed using a 1200 L Mass Spectrometer (Agilent Technologies, Inc., Santa Clara, CA) interfaced with a SIL-20AC HT Autosampler (Shimadzu Scientific Instruments, Inc., Columbia, MD), a ProStar HPLC system Model 210 (Agilent Technologies, Inc. Santa Clara, CA [1]. All assays were performed at the Analytical Pharmacology Laboratory at the University of Toledo. Mass spectrometry was performed using selective ion monitoring. Monitoring for bedaquiline followed transitions from the two precursor ions at 555.2 and 557.2 atomic mass units (amu) to the product ions at 523.1 and 525.1 amu, respectively [8]. The upper and lower limits of quantitation for bedaquiline were 8,000 and 20 ng/ml, respectively [8].

Mononuclear cells were isolated from heparinized blood using Leucosep tubes (Sigma Aldrich). Cell-associated bedaquiline and M2 concentrations, expressed as ng/10^6^ cells, were calculated by normalizing the concentrations in ng/mL to the number of cells per sample based on the concentration of DNA in each sample, assuming 6 pg DNA per cell.

Monitoring for rifampin followed the transition from the parent ion at 823.2 amu to its transition ion at 791.4 amu compared with the parent ion for the internal standard, rifampicin–d4 at 827.2 amu to its transition ion at 795.4 amu. Monitoring for rifabutin followed the transitions from 847.1 to 815.4 for rifabutin and 854.4 to 822.4 for rifabutin-d7. The upper limit of quantitation for rifampin was 20,000 ng/ml, whereas the lower limit of quantitation was 50 ng/ml. For rifabutin the upper limit of quantitation was 1000 ng/ml and the lower limit of quantitation was 10 ng/ml.

### WBA

Heparinized blood samples for WBA were collected immediately prior to bedaquiline dosing and at 1, 2, 3, 4, 6, 8, and 12 hrs post-dose. Blood was maintained at ambient temperature with slow constant mixing until transported to the laboratory after collection of the 12 hr specimen. Measurement of WBA was performed as previously described [9], using duplicate whole blood cultures. Briefly, *M tuberculosis* H37Rv was grown in the BACTEC MGIT system (Becton Dickinson, Sparks, MD) and frozen in aliquots at -80°C. A titration experiment determined the relationship between log inoculum volume and time to positivity (TTP) in MGIT, and identified the volume predicted to be positive in 5.5 days. For each set of whole blood cultures, the specified volume of *M tuberculosis* stock was inoculated directly into MGIT culture, as a control to assess viability. Results are flagged if stock viability decreases by 0.5 log or more compared to the titration curve. The whole blood cultures consisted of heparinized venous blood, an equal volume of RPMI 1640 tissue culture medium with l-glutamine and HEPES, 15nM dihydroxyvitamin D, and the specified volume of mycobacterial stock. Vitamin D at this concentration facilitates detection of activity of bedaquiline in whole blood culture but is insufficient to directly affect mycobacterial viability [4]. Whole blood cultures were incubated at 37°C for 72 hours, after which cells were collected by sedimentation, the liquid phase removed, and blood cells disrupted by hypotonic lysis. Bacilli were recovered, inoculated into MGIT, and incubated until flagged as positive. Log change in viability was calculated as log(*final*)–log(*initial*), where *final* and *initial* are the volumes corresponding to TTP of the completed cultures and the inoculum control, respectively, based on the titration curve. Results were expressed as log change in viability per day of whole blood culture (Δlog/d), with positive values indicating growth. The laboratory protocol is provided as a supplemental file.

Participants in this study received rifampin or rifabutin only in combination with bedaquiline. To better understand the concentration responses of these drugs and their metabolites individually, they were added directly to whole blood cultures of an additional healthy volunteer in a separate experiment, using ranges of concentrations spanning those encountered clinically.

### Qualification experiment

In preparation for the study, 2 batches of *M tuberculosis* stock were prepared. Titration experiments indicated satisfactory inoculum volumes of 12 and 6 μl, containing 5.6 x10^3^ and 2.1 x10^3^ CFU, respectively. A qualification experiment was performed using blood samples from one subject (RSW), obtained prior to and 2 hrs after a single oral dose of levofloxacin 500 mg. These results, showing 0.251 and -0.401 log/day, respectively, were consistent with previous experiments conducted in other laboratories using this donor and method. The findings overall were considered satisfactory to proceed with samples from study participants.

### Statistics and modeling

The area under the WBA curve (AUC) was calculated for each measurement interval using the trapezoid method (*ie*, the average WBA of 2 consecutive time points times the difference in hours between the time points, divided by 24). Cumulative WBA following drug administration was calculated by adding the AUC of each interval to the sum of those preceding it, starting at the origin (0,0). Results were expressed as Δlog/d • d or simply as log change.

The relationship between log drug concentration and WBA was examined using a 4 parameter (I_max_) equation describing a sigmoid curve, as previously reported [10]. Drug concentrations in whole blood culture were calculated as half those in plasma, due to dilution of blood with tissue culture medium. Curve fitting and statistical testing were performed using Sigmaplot. Correlations were tested by the Pearson product method. Modeling of the combined effects of bedaquiline plus either rifabutin or rifampin was initially performed assuming that drug effects would be independent and additive based on their individual concentration-response curves. Visual inspection of the results for bedaquiline plus rifabutin revealed this assumption was incorrect, and that accurate predictions at low drug concentrations required the introduction of a correction factor determined by linear regression. Subsequent modeling of bedaquiline PK at steady state included this correction factor.

Study data files are available as online appendices.

### Statistical power considerations

The required sample size of the parent trial was determined by its primary endpoint, bedaquiline pharmacokinetics. The statistical power of this WBA sub-study was estimated using data from 9 patients treated with standard TB therapy (daily HRZE), showing a cumulative WBA over 24 hrs of -0.466±0.118 [11]. Assuming similar variability in this trial (SD = .118), the probability was 80 percent that a study with 16 subjects per arm would detect a treatment difference between the 2 arms at a two-sided 0.05 significance level if the true difference between treatments was 0.121 (*ie*, ¼ of the effect of standard treatment). In a paired analysis, a smaller change (0.088) could be detected within each arm with similar power.

## Results

**Fig 2:**
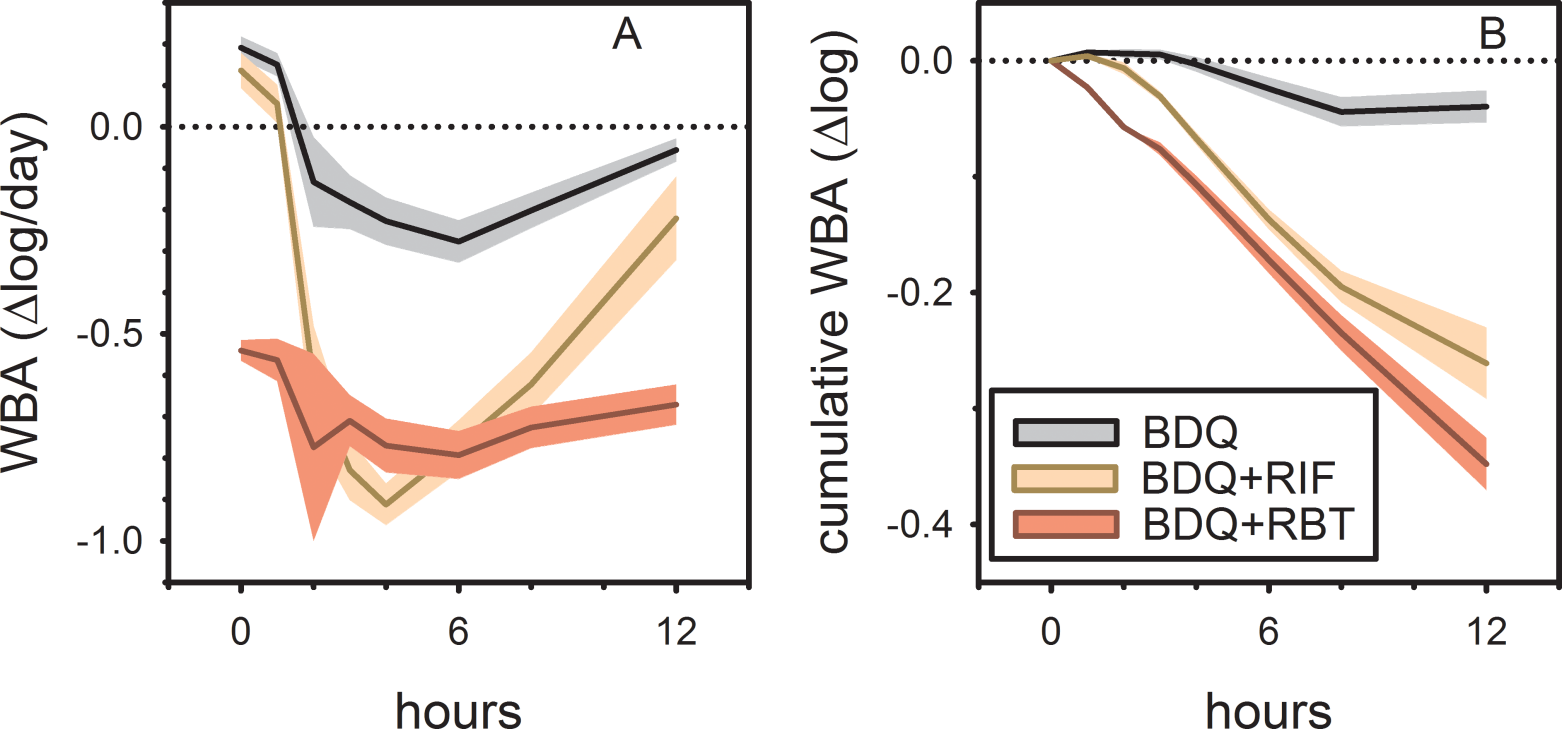
Mycobactericidal activity of single 400 mg doses of bedaquiline in ex vivo whole blood culture (WBA) in healthy volunteers, alone (gray), or combined with either rifabutin 300 mg (RBT, red) or rifampin 600 mg (RIF, yellow), after 9 days of rifamycin dosing. Panel A shows change in viability from beginning to end of each whole blood culture, expressed as log change per day of culture; panel B shows cumulative or total activity over the 12 hours following drug administration. Lines indicate mean values; shading indicates 90% confidence intervals.

At baseline, study participants showed intracellular growth of *M tuberculosis* of 0.19 log/day in whole blood culture, approximately half that expected in enriched broth culture. Oral administration of a single dose of 400 mg bedaquiline on day 1 resulted in the gradual expression of mycobactericidal activity, reaching a maximal effect of -0.28 log/day at 6 hrs post dose (gray curve, Fig 2 panel A). The vertical axis in this figure indicates the change in viability from beginning to end of each whole blood culture, expressed as log change per day of culture. The effect of bedaquiline subsequently declined but did not return to baseline by 12 hours post dose. Prior to dosing on day 29, subjects assigned to rifampin showed growth of *M tuberculosis* very similar to baseline values (0.14 log/day, yellow curve, Fig 2 panel A). Administration of bedaquiline plus rifampin resulted in rapid expression of mycobactericidal activity, reaching a maximal effect of -0.91 log/day at 4 hours post dose. This effect declined by 12 hours to values approaching those of bedaquiline alone. In contrast, subjects assigned to bedaquiline plus rifabutin showed substantial mycobactericidal activity prior to dosing on day 29 (-0.54 log/day, red curve, Fig 2 panel A). The maximum effect of the two drugs was not reached until 6 hours post dose; at 12 hours, these values had not yet returned to baseline. The flattened shape of the bedaquiline+rifabutin curve, showing persistent activity at 0 and 12 hrs, is consistent with the long plasma T½ of rifabutin.

In Fig 2 panel B, WBA data from static drug concentrations at discrete time points (those in Fig 2A) are integrated over time to show evolution of total effect (AUC) over time. This method creates a dynamic (*ie*, concentration varying) time-kill model from static data. The vertical axis in Fig 2 panel B indicates cumulative or total activity since the moment of drug administration. The curves in this figure indicate a significantly greater cumulative effect of the combination of bedaquiline plus rifabutin as compared to bedaquiline plus rifampin over the 12 hour interval.

**Fig 3:**
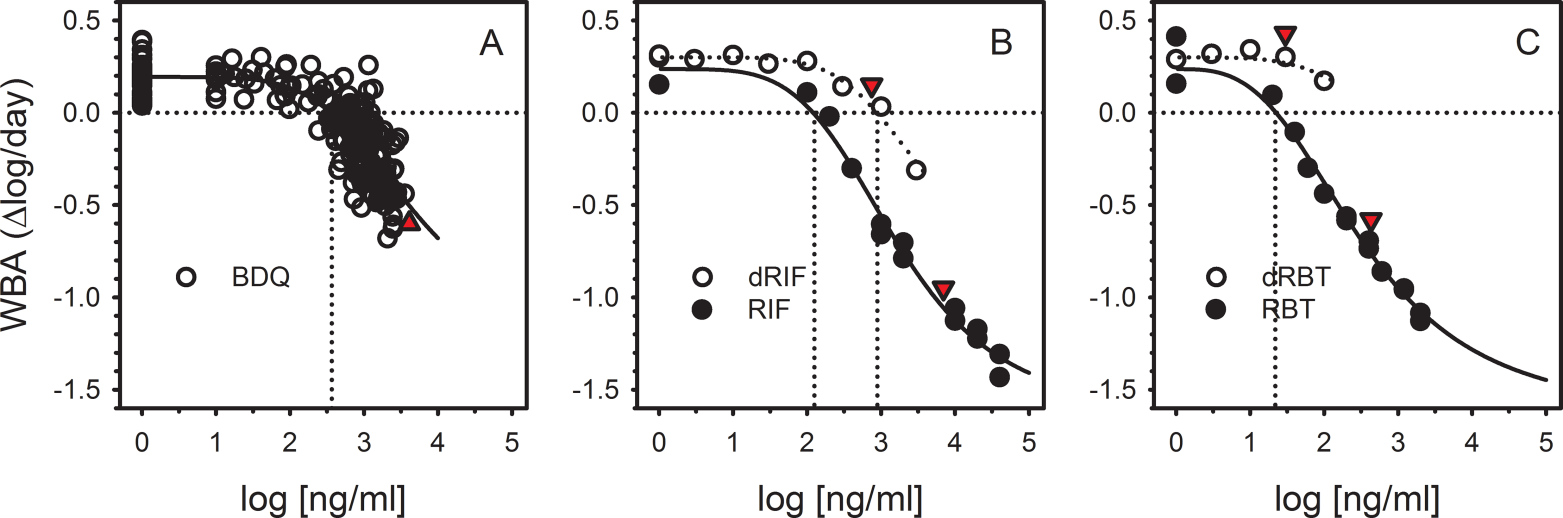
Relationship between drug concentration and whole blood bactericidal activity (WBA) for bedaquiline (panel A), rifampin (panel B), and rifabutin (panel C). Panels B and C also indicate activity of desacetyl rifamycin metabolites (open circles). All drug concentrations reflect those in whole blood culture. For bedaquiline, measured plasma concentrations were reduced by a factor of two to account for dilution with tissue culture medium in whole blood culture. Rifamycins and their metabolites were added directly to whole blood cultures at the indicated concentrations. Red triangles indicate plasma Cmax values, which have also been adjusted to account for dilution in whole blood culture. Vertical dotted lines indicate concentrations required for intracellular mycobacteriostasis (zero WBA).

Several analyses were performed to better understand the basis and significance of this observation. We first examined the relationship between drug concentrations and activity, singly and in combination. The relationship between bedaquiline plasma concentration and mycobactericidal activity, from paired analyses of single specimens on day 1, is shown in Fig 3A. A highly significant relationship (*p*<0.0001) was observed, with a plasma concentration of 355 ng/ml identified as that required for intracellular mycobacteriostasis. For rifampin and rifabutin, the relationship between drug concentration and activity was determined by separate experiments in which these drugs were added directly to whole blood cultures of an additional donor, over a wide range of concentrations (filled circles, Fig 3B and 3C). Concentrations of 120 and 22 ng/ml were required for intracellular mycobacteriostasis for rifampin and rifabutin, respectively. Both values were well below C_max_ values for these drugs in the trial (red triangles). Mammalian metabolism of rifampin and rifabutin results in biologically active desacetyl derivatives, which were also tested by direct addition to whole blood culture. In contrast to the parent drugs, C_max_ concentrations of the rifamycin metabolites were insufficient to yield mycobactericidal effects (open circles, Fig 3B and 3C), although in the case of desacetyl rifampin, clinically achieved concentrations approached those needed for mycobacteriostasis.

**Fig 4:**
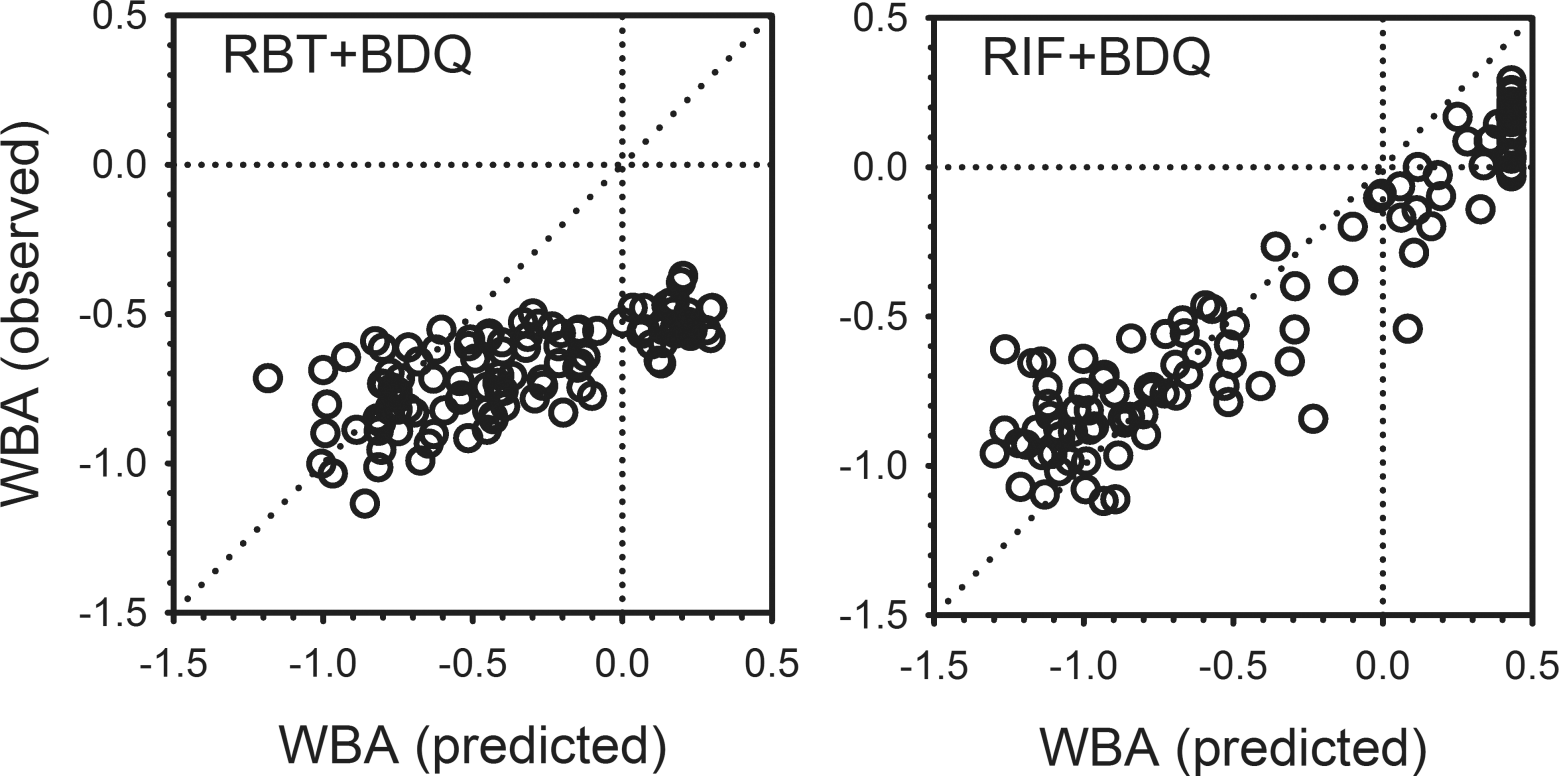
Whole blood bactericidal activity (WBA) observed for bedaquiline plus either rifabutin (left) or rifampin (right) in relation to that predicted as the sum of the activities of each drug alone, based on plasma concentrations.

Having established the relationships between concentration and activity for bedaquiline, rifampin, and rifabutin tested individually, the findings in blood samples obtained during combined dosing on Day 29 were then examined in relation to predicted activity based on the summed effects of each drug alone (Fig 4). In the case of rifampin plus bedaquiline, the observed activity was close to that predicted as the sum of each individually (right panel). However, for rifabutin plus bedaquiline, the observed activity was as much as -0.5 log better than predicted (lower right quadrant, left panel). The discrepancy was greatest in samples obtained prior to and shortly after dosing, at which times the measured concentrations of both drugs were low. For example, the majority of data points in the lower right quadrant of the left panel are from 0 hr (pre-dose) specimens, in which the mean log concentration of rifabutin was 1.65 (45 ng/ml). Only four specimens at this time point had measurable bedaquiline, with a maximum concentration of log 1.54 ng/ml (35 ng/ml). At these concentrations, rifabutin would be anticipated to produce a measurable, albeit small effect alone, whereas no effect would be expected from bedaquiline. Only one specimen had measurable desacetyl rifabutin, at a log concentration of 0.77 (5.9 ng/ml), insufficient to produce a detectable effect. Seventeen specimens had measurable bedaquiline M2 metabolite, with a log mean of 0.90 (8 ng/ml). The potential contribution of the M2 metabolite at this concentration is not known.

**Fig 5:**
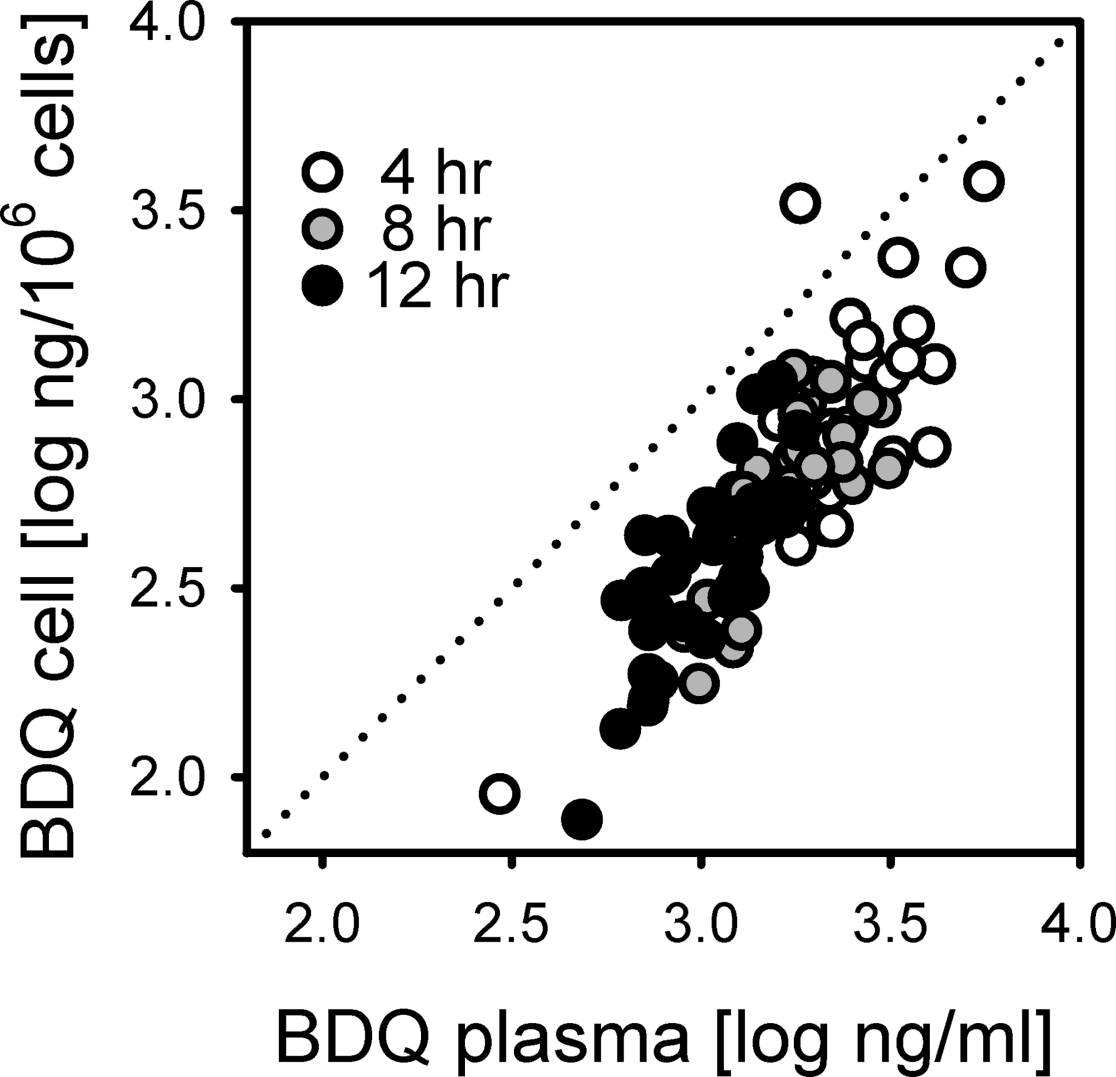
Relationship between measured bedaquiline concentrations in plasma and in blood mononuclear cells.

Differential cellular bedaquiline accumulation was also considered as a possible explanation for the discrepancy. Cell-associated and plasma concentrations of bedaquiline were highly correlated (R^2^ = 0.70, Fig 5). Concentrations of cell-associated bedaquiline were below the level of quantitation in all cell specimens obtained prior to dosing on Day 29, as were all but one M2 concentrations. Thus neither metabolites nor cellular accumulation appeared to account for the more-than-additive interaction of bedaquiline plus rifabutin.

**Fig 6:**
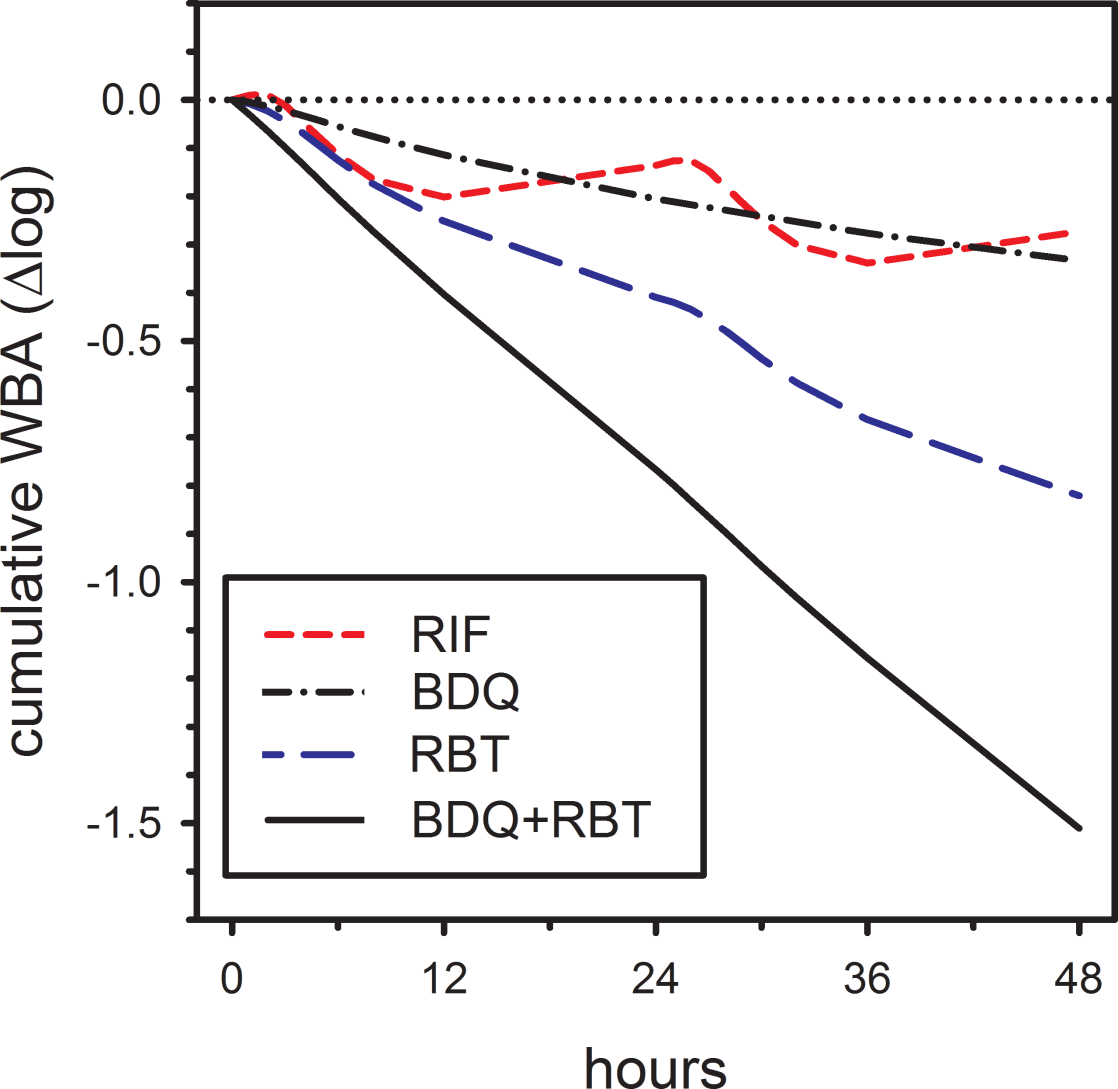
Predicted cumulative whole blood bactericidal activity (WBA) of bedaquiline, rifampin, rifabutin, and the combination of bedaquiline plus rifabutin, based on expected plasma concentrations in vivo during TB treatment, and observed more-than-additive interactions.

To better understand the potential significance of this observation, a linear correction factor was introduced into the prediction of the combined activity of rifabutin plus bedaquiline, taking the form of *a*+*b*x, where x was the sum of the individual effects. Values for *a* and *b* were solved by linear regression. The resulting corrected regression curve precisely predicted the measured effects of rifabutin plus bedaquiline, *ie*, its slope was 1.0 and its intercept was 0.0. This corrected equation was then used to predict the activity of rifabutin plus bedaquiline as it might occur *in vivo* during TB treatment. Results of this modeling are shown in Fig 6. Like Fig 1B, this prediction accounts for the unexpectedly more-than-additive interaction of rifabutin plus bedaquiline. However, it differs from Fig 1B in two key respects. First, plasma bedaquiline concentrations reflect steady state conditions at eight weeks of treatment, at a dose of 200 mg thrice weekly [3], rather than after single 400 mg doses. Secondly, for both bedaquiline and rifabutin, predicted effects reflect concentrations achieved *in vivo* rather than those in 1:1 diluted whole blood cultures. In this model, bedaquiline and rifabutin alone were predicted to reduce *M tuberculosis* viability by -0.16 and -0.45 log per day. Together their combined total effect was -0.75 log per day, -0.14 more than their sum.

## Discussion

This study examined the pharmacodynamic interactions in healthy volunteers of bedaquiline with rifabutin and rifampin in relation to their pharmacokinetics. The main findings were that the combination of bedaquiline plus rifabutin produced greater *ex vivo* mycobactericidal activity than bedaquiline plus rifampin, and that low concentrations of bedaquiline plus rifabutin produced greater combined activity than expected based on their individual effects. Together, these two drugs were predicted to yield WBA of -0.75 log per day *in vivo*. A similar modeling exercise for sutezolid, a novel oxazolidinone being developed for TB, predicted a corresponding *in vivo* effect of -0.27 log per day in patients [10]. Thus, rifabutin plus bedaquiline show nearly three times the effect of sutezolid alone.

WBA is a candidate biomarker for assessment of protective antimycobacterial immunity and curative chemotherapy [12]. Mycobacteria added to whole blood cultures are rapidly and completely ingested by neutrophils and monocytes [13]. The resulting *in vitro* immune response, with associated T cell activation and cytokine production, typically leads to restriction of intracellular mycobacterial growth but generally not bacterial killing [14]. Concentrations of administered drugs (and their metabolites) in the whole blood cultures reflect those in the circulation at the time of phlebotomy. The model thus has important similarities and differences when compared to the macrophage infection model. Cumulative WBA is greater during standard tuberculosis treatment *vs* typical MDR regimens, in inverse proportion to the duration of treatment required for durable cure [13]. Cumulative WBA during standard tuberculosis treatment correlates with two month culture status [15]; culture status and treatment duration are, in turn, predictors of relapse risk [16, 17]. These findings therefore indicate that the substitution of rifabutin plus bedaquiline for rifampin has the potential to shorten drug-susceptible tuberculosis treatment.

Rifabutin was approved by the US FDA in 1992 for prevention of disseminated *M avium* disease in patients with advanced AIDS. Its main use at present is in the treatment of tuberculosis in patients requiring concomitant treatment with HIV protease inhibitors. A Cochrane review by Davies *et al* identified 5 tuberculosis treatment trials with a total of 924 participants in which rifabutin and rifampin were compared [18]. There were no statistically significant differences in rates of cure at end of treatment or rates of relapse. One study with 225 evaluable patients reported rates of sputum culture positivity at eight weeks using solid culture medium of 8.0% in the rifabutin arm and 12.3% in the rifampin arm [19]. Although this finding is favorable, it falls short of the 1% rate proposed as a target for new 4 month tuberculosis regimens [16, 17]. Studies examining the effects of rifabutin plus bedaquiline on sputum culture conversion can help further elucidate its treatment shortening role in DS-TB.

Effective use of rifabutin with bedaquiline will require a thoughtful balancing of the potential risks and benefits of this combination. As we reported elsewhere, several instances of lymphopenia occurred in healthy volunteers assigned to bedaquiline and rifabutin in this trial [8]. Other rifamycins, such as rifapentine, similarly appear to be less well tolerated in healthy volunteers than in tuberculosis patients [20]. Additional studies will be required to better understand the safety of bedaquiline plus rifabutin in tuberculosis patients and to balance these risks against potential therapeutic benefits.

Lastly, a possible role for rifabutin in the treatment of MDR-TB has been described for *M tuberculosis* strains with rifampin resistance due to mutations at codon 516 in *rpoB* [21–23]. These strains, which appear to remain susceptible to rifabutin despite resistance to rifampin, predominate among MDR-TB isolates in the Eastern Cape region of South Africa. They can be detected by the Hain MTBDRplus LIPA test, the B probe of the Cepheid Xpert system, or by phenotypic susceptibility testing. Clinical trials of eight weeks duration in this patient population may be considered as a strategy to advance the testing of the rifabutin plus bedaquiline combination while the safety concerns are being evaluated.

## Supporting information

S1 File CONSORT 2010 checklist. (DOC)

S2 File PK Data. (XLSX)

S3 File WBA PK analysis. (XLSM)

S4 File Clinical protocol. (PDF)

S5 File WBA protocol. (PDF)

## References

[1] World Health Organization. Global tuberculosis report 2017. Geneva; 2017. Report No.: WHO/HTM/TB/2017.23 Contract No.: WHO/HTM/TB/2017.23.

[2] Andries K, Verhasselt P, Guillemont J, Gohlmann HW, Neefs JM, Winkler H, et al A diarylquinoline drug active on the ATP synthase of Mycobacterium tuberculosis. Science. 2005;307(5707):223–7. doi: 10.1126/science.1106753 15591164 10.1126/science.1106753

[3] Diacon AH, Pym A, Grobusch M, Patientia R, Rustomjee R, Page-Shipp L, et al The diarylquinoline TMC207 for multidrug-resistant tuberculosis. N Engl J Med. 2009;360(23):2397–405. doi: 10.1056/NEJMoa0808427 19494215 10.1056/NEJMoa0808427

[4] Wallis RS, Jakubiec W, Mitton-Fry M, Ladutko L, Campbell S, Paige D, et al Rapid Evaluation in Whole Blood Culture of Regimens for XDR-TB Containing PNU-100480 (Sutezolid), TMC207, PA-824, SQ109, and Pyrazinamide. PLoS ONE. 2012;7(1):e30479 doi: 10.1371/journal.pone.0030479 22279595 10.1371/journal.pone.0030479 PMC3261206

[5] Tasneen R, Li SY, Peloquin CA, Taylor D, Williams KN, Andries K, et al Sterilizing activity of novel TMC207- and PA-824-containing regimens in a murine model of tuberculosis. Antimicrob Agents Chemother. 2011;55(12):5485–92. doi: 10.1128/AAC.05293-11 21930883 10.1128/AAC.05293-11 PMC3232786

[6] Svensson EM, Murray S, Karlsson MO, Dooley KE. Rifampicin and rifapentine significantly reduce concentrations of bedaquiline, a new anti-TB drug. J Antimicrob Chemother. 2015;70(4):1106–14. doi: 10.1093/jac/dku504 25535219 10.1093/jac/dku504 PMC4356204

[7] Winter H, Egizi E, Murray S, Erondu N, Ginsberg A, Rouse DJ, et al Evaluation of the pharmacokinetic interaction between repeated doses of rifapentine or rifampin and a single dose of bedaquiline in healthy adult subjects. Antimicrob Agents Chemother. 2015;59(2):1219–24. doi: 10.1128/AAC.04171-14 25512422 10.1128/AAC.04171-14 PMC4335826

[8] Healan AM, Griffiss JM, Proskin HM, O'Riordan MA, Gray WA, Salata RA, et al Impact of Rifabutin or Rifampin on Bedaquiline Safety, Tolerability, and Pharmacokinetics Assessed in a Randomized Clinical Trial with Healthy Adult Volunteers. Antimicrob Agents Chemother. 2018;62(1). 10.1128/AAC.00855-17 PMC5740348 29061739

[9] Wallis RS, Jakubiec W, Kumar V, Bedarida G, Silvia A, Paige D, et al Biomarker assisted dose selection for safety and efficacy in early development of PNU-100480 for tuberculosis. Antimicrob Agents Chemother. 2011;55(2):567–74. doi: 10.1128/AAC.01179-10 21078950 10.1128/AAC.01179-10 PMC3028776

[10] Zhu T, Friedrich SO, Diacon A, Wallis RS. Population Pharmacokinetic/Pharmacodynamic Analysis of the Bactericidal Activities of Sutezolid (PNU-100480) and Its Major Metabolite against Intracellular Mycobacterium tuberculosis in Ex Vivo Whole-Blood Cultures of Patients with Pulmonary Tuberculosis. Antimicrob Agents Chemother. 2014;58(6):3306–11. doi: 10.1128/AAC.01920-13 24687496 10.1128/AAC.01920-13 PMC4068491

[11] Wallis RS, Dawson R, Friedrich SO, Venter A, Paige D, Zhu T, et al Mycobactericidal activity of sutezolid (PNU-100480) in sputum (EBA) and blood (WBA) of patients with pulmonary tuberculosis. PLoS One. 2014;9(4):e94462 doi: 10.1371/journal.pone.0094462 24732289 10.1371/journal.pone.0094462 PMC3986205

[12] Wallis RS. Assessment of whole blood bactericidal activity in the evaluation of new TB drugs In: Donald PR, Van Helden P, editors. Antituberculosis Chemotherapy Progressin Respiratory Research. 40. Basel: Karger; 2011 p. 1–7.

[13] Wallis RS, Palaci M, Vinhas S, Hise AG, Ribeiro FC, Landen K, et al A whole blood bactericidal assay for tuberculosis. J Infect Dis. 2001;183(8):1300–3. doi: 10.1086/319679 11262217 10.1086/319679

[14] Fletcher HA, Tanner R, Wallis RS, Meyer J, Manjaly ZR, Harris S, et al Inhibition of mycobacterial growth in vitro is enhanced following primary BCG vaccination but not BCG revaccination of human subjects. Clin Vaccine Immunol. 2013;20(11):1683–9. doi: 10.1128/CVI.00427-13 23986316 10.1128/CVI.00427-13 PMC3837779

[15] Wallis RS, Vinhas SA, Johnson JL, Ribeiro FC, Palaci M, Peres RL, et al Whole blood bactericidal activity during treatment of pulmonary tuberculosis. J Infect Dis. 2003;187:270–8. doi: 10.1086/346053 12552451 10.1086/346053

[16] Wallis RS, Wang C, Meyer D, Thomas N. Month 2 culture status and treatment duration as predictors of tuberculosis relapse risk in a meta-regression model. PLoS ONE. 2013;8(8):e71116 doi: 10.1371/journal.pone.0071116 23940699 10.1371/journal.pone.0071116 PMC3733776

[17] Wallis RS, Peppard T, Hermann D. Month 2 culture status and treatment duration as predictors of recurrence in pulmonary tuberculosis: model validation and update. PLoS One. 2015;10(4):e0125403 doi: 10.1371/journal.pone.0125403 25923700 10.1371/journal.pone.0125403 PMC4414505

[18] Davies G, Cerri S, Richeldi L. Rifabutin for treating pulmonary tuberculosis. Cochrane Database Syst Rev. 2007(4):CD005159 doi: 10.1002/14651858.CD005159.pub2 17943842 10.1002/14651858.CD005159.pub2 PMC6532710

[19] McGregor MM, Olliaro P, Wolmarans L, Mabuza B, Bredell M, Felten MK, et al Efficacy and safety of rifabutin in the treatment of patients with newly diagnosed pulmonary tuberculosis. Am J Respir Crit Care Med. 1996;154(5):1462–7. doi: 10.1164/ajrccm.154.5.8912765 8912765 10.1164/ajrccm.154.5.8912765

[20] Dooley KE, Savic RM, Park JG, Cramer Y, Hafner R, Hogg E, et al Novel dosing strategies increase exposures of the potent antituberculosis drug rifapentine but are poorly tolerated in healthy volunteers. Antimicrob Agents Chemother. 2015;59(6):3399–405. doi: 10.1128/AAC.05128-14 25824215 10.1128/AAC.05128-14 PMC4432148

[21] Sirgel FA, Warren RM, Bottger EC, Klopper M, Victor TC. The rationale for using rifabutin in the treatment of MDR and XDR tuberculosis outbreaks. PLoS One. 2013;8(3):e59414 doi: 10.1371/journal.pone.0059414 23527189 10.1371/journal.pone.0059414 PMC3602005

[22] Lee H, Ahn S, Hwang NY, Jeon K, Kwon OJ, Huh HJ, et al Treatment outcomes of rifabutin-containing regimens for rifabutin-sensitive multidrug-resistant pulmonary tuberculosis. Int J Infect Dis. 2017;65:135–41. doi: 10.1016/j.ijid.2017.10.013 29224631 10.1016/j.ijid.2017.10.013

[23] Jo KW, Ji W, Hong Y, Lee SD, Kim WS, Kim DS, et al The efficacy of rifabutin for rifabutin-susceptible, multidrug-resistant tuberculosis. Respir Med. 2013;107(2):292–7. doi: 10.1016/j.rmed.2012.10.021 23199704 10.1016/j.rmed.2012.10.021

