# Supplementary material for: Mycobactericidal activity of bedaquiline plus rifabutin or rifampin in ex vivo whole blood cultures of healthy volunteers: A randomized controlled trial
Source: PLoS One. 2018 May 2;13(5):e0196756. doi: 10.1371/journal.pone.0196756 (PMC5931679; doi:10.1371/journal.pone.0196756)
Supplement: S4 File — (PDF) [file pone.0196756.s004.pdf]

A PHASE I OPEN-LABEL TRIAL TO INVESTIGATE THE PHARMACOKINETIC INTERACTION BETWEEN RIFABUTIN OR RIFAMPIN AND A SINGLE DOSE OF TMC207 IN HEALTHY SUBJECTS

DMID Protocol Number: 10-0043

DMID Funding Mechanism: Contract #HHSN27220080026C

Pharmaceutical Support Provided by: TB Alliance

IND Sponsor:

National Institutes of Health

Division of Microbiology and Infectious Diseases

6610 Rockledge Drive

Bethesda, MD

Principal Investigator: John McLeod Griffiss, MD

DMID Clinical Project Manager: Tena Knudsen, RN

DMID Medical Monitor: Soju Chang, MD

Version Number: 7.0

Day Month Year: 21 February 2012

Statement of Compliance

This study will be carried out in accordance with the United States (US) Code of Federal Regulations (CFR), local regulations, and Good Clinical Practice (GCP) as required by the following:

- US CFR applicable to clinical studies (45 CFR 46; and 21 CFR including part 50 and 56 concerning informed consent and institutional review board (IRB) regulations, 21 CFR 11 concerning electronic records, 21 CFR 812 concerning devices, and 21 CFR 312 if under investigational new drug (IND)

- International Conference on Harmonisation (ICH) E6 (R1); 62 Federal Register 25691 (1997)

Signature Page 1

The signature below constitutes approval of this protocol and the attachments and provides the required assurances that this trial will be conducted according to all stipulations of the protocol, including all statements regarding confidentiality, and according to local legal and regulatory requirements, applicable US federal regulations, and (ICH E6 [R1]) guidelines.

Global Principal Investigator: J. Macleod Griffiss, MD

Signed: ______________________________ Date: ______________

Name/Title

Signature Page 2

The signature below constitutes approval of this protocol and the attachments and provides the required assurances that this trial will be conducted according to all stipulations of the protocol, including all statements regarding confidentiality, and according to local legal and regulatory requirements, applicable US federal regulations, and (ICH E6 [R1]) guidelines.

Principal Investigator or Investigator of Record*:* Robert A. Salata, MD

Signed: Date:

Name/Title

Table of Contents

1 Key Roles 15
2 Background Information and Scientific Rationale 18
2.1 Background Information 18
2.2 Scientific Rationale 19
2.3 Preclinical Studies 20
2.4 Phase 1 and 2 Trials 23
2.5 Rifampin and Rifabutin 28
2.6 Potential Risks/Benefits 29
2.6.1 Potential Risks 30
2.6.2 Known Potential Benefits 30
2.6.3 Risk/Benefit Ratio 31
3 Study Objectives 32
4 Study Design 33
4.1 Study Outcome Measures 34
4.1.1 Primary Outcome Measures 35
5 Study Enrollment and Withdrawal 36
5.1 Inclusion Criteria 36
5.2 Exclusion Criteria 37
5.2.1 Study Restrictions 39
5.3 Treatment Assignment Procedures 40
5.3.1 Randomization Procedures 41
5.3.2 Blinding Procedures 41
5.4 Withdrawal 41
5.4.1 Reasons for Withdrawal 41
5.5 Termination of the Study 43
6 Investigational Product(s) 44
6.1 Study Product(s) Description 44
6.1.1 Formulation, Packaging, and Labeling 44
6.1.2 Acquisition 45
6.1.3 Product Storage, Stability, and Expiration 45
6.1.4 Preparation/Handling 45
6.1.5 Administration 45
6.1.6 Accountability/Final Disposition for the Investigational Product(s) 46
7 Study Procedures/Evaluations/Schedule 48
7.1 Clinical/Laboratory Evaluations and Study Schedule 48
7.2 Screening 51
7.3 Enrollment/Baseline 53
7.4 Study Visits 53
7.5 Follow-up Visit 60
7.6 Unscheduled Visit(s) 61
7.7 Early Termination 61

7.8 Final Study Visit 62
7.9 Rescue Therapy 62
7.10 Specimen Preparation, Handling, and Shipping 62
7.10.1 Instructions for Specimen Preparation, Handling, and Storage 62
7.10.2 Specimen Shipment 63
8 DMID Safety Reporting and Safety Monitoring 64
8.1 Responsibilities 64
8.2 Definitions 64
8.3 Safety Reporting Requirements 67
8.3.1 Reporting Interval 67
8.3.2 Notification of the Sponsor of Serious Adverse Events 67
8.3.3 Regulatory Reporting for Studies Conducted Under DMID-Sponsored IND 68
8.3.4 Reporting of Pregnancy 68
8.4 Investigator's Assessment of Adverse Events 69
8.4.1 Assessment of Seriousness 69
8.4.2 Assessment of Severity 69
8.4.3 Assessment of Relationship to Study Products 70
8.5 Safety Monitoring by the DMID Safety Oversight Mechanism 71
8.5.1 Safety Monitoring Committee 72
8.5.2 Interruption or Discontinuation of Study Enrollment and Study Product Administration for all Subjects in the Study 72
8.6 Halting Criteria/Rules 73
9 Clinical Management of Events 75
9.1 Adverse Event Management 75
9.1.1 Temporary Interruption of Study Product in an Individual Subject 75
9.1.2 Pregnancy (if applicable) 75
10 Clinical Monitoring/Site Monitoring Plan 76
11 Statistical Considerations 77
11.1 Study Hypotheses 78
11.2 Sample Size Considerations 79
11.3 Randomization 79
11.4 Blinding 79
11.5 Planned Interim Analyses (if applicable) 79
11.6 Safety Review 79
11.7 Final Analysis Plan 79
12 Data Handling/Record Keeping/Source Documents 81
12.1 Data Capture Methods 81
12.2 Types of Data 82
12.3 Timing and Reports 82
12.4 Study Records Retention 82
12.5 Source Documents 83
12.6 Protocol Deviations 83

13 Quality Control and Quality Assurance....84
14 Ethics/Protection of Human Subjects....85
14.1 Ethical Standard....85
14.2 Institutional Review Board....85
15 Informed Consent Process....87
15.1 Subject Confidentiality....87
15.2 Principal Investigator Responsibility When Subject Withdraws or is Discontinued....88
15.3 Future Use of Stored Specimens....88
16 Publication Policy....89
17 Literature References....90
APPENDIX A: SCHEDULE OF EVENTS....92
APPENDIX B: BLOOD SAMPLING VOLUMES....103
APPENDIX C: TOXICITY TABLE....110
APPENDIX D: SUPPLEMENT TABLE FOR LABS & REFERENCE RANGES....123
APPENDIX F: SUMMARY OF PHARMACOKINETIC DATA AVAILABLE FOR M2....159

List of Abbreviations

AE	Adverse event
ALP	Alkaline phosphatase
ALT	Alanine aminotransferase
APTT	Activated partial thromboplastin time
AST	Aspartate aminotransferase
AUC	Area under the time-concentration curve
BMI	Body mass index
Bpm	Beats per minute
BLQ	Below the limit of quantification
BR	Background regimen
BUN	Blood urea nitrogen
CAD	Cationic amphiphilic drug
CFR	Code of Federal Regulations
CFU	Colony forming unit
CIs	Confidence intervals
C_{max}	Maximum plasma concentration
$C_{max\ (reference)}$	Maximum plasma concentration of TMC207 alone
$C_{max\ (test)}$	Maximum plasma concentration of TMC207 in combination with rifabutin or rifampin
CPK	Creatine phosphokinase
CPK-MB	Creatine phosphokinase for muscle-brain
CRF	Case report form
CRM	Clinical Research Management

CROMS	Clinical research operations and management support
CTSA	Clinical and Translational Science Awards
CYP	Cytochrome P450
DCRU	Dahms Clinical Research Unit
DHHS	Department of Health and Human Services
DMID	Division of Microbiology and Infectious Diseases, NIAID, NIH Adult Toxicity Table
DOTS	Directly observed therapy
EBA	Early bactericidal activity
eEBA	Extended EBA
ECG	Electrocardiogram
FDA	Food and Drug Administration
GCP	Good clinical practice
GLP	Good laboratory practice
GMR	Geometric mean ratio
HBsAg	Hepatitis B surface antigen
HDYF	How do you feel?
HCG	Human chorionic gonadotropin
HCV	Hepatitis C virus
HIPAA	Health Insurance Portability and Accountability Act
HIV-1(2)	Human immunodeficiency virus-type 1 (type 2)
HPLC	High-performance liquid chromatography
IB	Investigator’s brochure
ICF	Informed consent form

ICH	International Conference on Harmonisation
IgM	Immunoglobulin
IMP	Investigational medicinal product
IND	Investigational new drug
INH	Isoniazid
INR	International normalized ratio
IRB	Institutional review board
ISM	Independent safety monitor
IUD	Intrauterine device
Kel	Apparent elimination rate constant
LC-MS/MS	Liquid chromatography-mass spectrometry/mass spectrometry
LDH	Lactate dehydrogenase
LLN	Lower limit of laboratory normal range
LPV/rtit	Lopinavir/Ritonavir
LS	Least square
M2	N-monodesmethyl metabolite of TMC207
MDR-TB	Multidrug-resistant tuberculosis
MedDRA	Medical Dictionary for Regulatory Activities
MGIT	*Mycobacteria* growth indicator tube
MIC	Minimum inhibitory concentration
MOP	Manual of procedures
MPS	Mononuclear phagocytic system
MTB	*Mycobacterium tuberculosis*

N	Number (typically refers to number of subjects)
NIAID	National Institute of Allergy and Infectious Diseases
NIH	National Institute of Health
NOAEL	No observable adverse effect level
NRTI	Nucleoside/Nucleotide Reverse Transcriptase Inhibitors
NVP	Nevirapine
OCRA	Office of Clinical Research Affairs, DMID, NIAID, NIH, DHHS
OHRP	Office for Human Research Protections, DHHS
OHSR	Office for Human Subjects Research, NIH, DHHS
ORA	Office of Regulatory Affairs, DMID, NIAID, NIH, DHHS
PBMC	Peripheral blood mononuclear cells
PD	Pharmacodynamic
pH	Measurement of acidity or alkalinity
PHI	Protected health information
PI	Principal investigator
PK	Pharmacokinetic
PT	Prothrombin time
PZA	Pyrazinamide
QA	Quality assurance
QC	Quality control
QMC	Quality Monitoring Committee
q.d.	Quaque die; once daily
QTcB	QT interval corrected for heart rate according to Bazett

QTcF	QT interval corrected for heart rate according to Fridericia
RBC	Red blood cell
REDIARC	Retinal Diseases Image Analysis Reading Center
RLS	Resource limited settings
RMP	Rifampin
RMSE	Root mean square error
SAE	Serious adverse event
SAP	Statistical analysis plan
SD	Standard deviation
SMC	Safety monitoring committee
SSCC	Serial sputum colony count
TB	Tuberculosis
TBTC	Tuberculosis Trial Consortium of the U.S. CDC
TMC	Tibotec medicinal compound
T_{max}	Time to peak plasma concentration
$t_{1/2}$	Apparent elimination half-life
UA	Urinalysis
UDP-GT	Uridine diphosphoglucuronosyl transferase
UHCMC	University Hospital Case Medical Center
ULN	Upper limit of laboratory normal range
WBA	Whole blood bactericidal assay
WHO	World Health Organization

Protocol Summary

**Title**	A Phase I open-label trial to investigate the pharmacokinetic interaction between Rifabutin or Rifampin and a single dose of TMC207 in healthy subjects
**Abbreviated Title**	TMC207 ± Rifabutin/Rifampin
**Phase**	1
**Population**	32 (16 per treatment group) healthy male or female subjects, 18 – 45 years old
**Number of Sites**	1: Case Western Reserve University/University Hospitals Case Medical Center, 11100 Euclid Avenue, Cleveland, OH, 44106
**Study Duration**	52 weeks; with Subject Participant Duration being approximately 60 days (up to 21 days for screening to admission, includes 28-day follow-up after second TMC207 dose).
**Subject Participation Duration**	Screening to admission: up to 21 days<br>In study: 60 (±3) days (includes 28-day follow-up after 2nd TMC207 dose)
**Agent or Intervention**	Subjects will receive two single oral doses of 400mg TMC207, first on Study Day 1 followed by a 28-day wash-out, the second on Study Day 29. Rifabutin 300mg (Group 1) or rifampin 600mg (Group 2) will be administered once daily during Period 2 from Study Day 20 through Study Day 41. The primary endpoint for the study will be determined on the final study visit, Day 57 (28 days after the last TMC207 dose in Period 2).
**Objectives**	Primary:<br>• To evaluate the effect of repeated doses of 300mg rifabutin or 600mg rifampin on the pharmacokinetics of TMC207(and its M2 metabolite) given as a single dose in healthy subjects<br>• To evaluate the safety and tolerability of TMC207 (and its M2 metabolite) when given with 300mg rifabutin or 600mg rifampin<br>Exploratory:<br>• To evaluate the cell-associated levels of TMC207 and its M2 metabolite in peripheral blood mononuclear cells (PBMCs) after single and multiple doses in healthy subjects<br>• To evaluate whole blood bactericidal assays in subjects receiving TMC207 alone or in combination with rifampin or rifabutin.
**Safety**	Physical examinations, vital signs, ECGs, adverse events, hematology,

**Endpoints**	coagulation, serum chemistry, eye exams, and urinalysis will be used to assess safety and tolerability of TMC207.

Table 1: **Descriptive Schematic of Study Design**

	**Period 1**	**Wash-out**	**Period 2**
**Group 1** (n=16)	Single oral dose of TMC207 (400mg) on Study Day 1	4 weeks between subsequent TMC207 doses	Daily oral doses of **rifabutin** (300mg) on Study Days 20 to 41 plus a single oral dose of TMC207 (400mg) on Study Day 29
**Group 2** (n=16)			Daily oral doses of **rifampin** (600mg) on Study Days 20 to 41 plus a single oral dose of TMC207 (400mg) on Study Day 29

1 KEY ROLES

Principal Investigator

John McLeod Griffiss, MD
Global Principal Investigator
Clinical Research Management, Inc. (CRM)
1265 Ridge Road, Suite A
Hinckley, OH 44233
415-476-5371
jgriffiss@clinicalrm.com
crapaud@loursage.org

Site Investigator

Robert A. Salata, MD
Site Principal Investigator
Case Western Reserve University
University Hospitals Case Medical Center
11100 Euclid Avenue
Cleveland, OH 44106-5083
216-844-6279
ras7@case.edu

Study Pharmacologist

Jeffrey Blumer, MD, PhD
Protocol Pharmacologist
University of Toledo College of Medicine
The Toledo Children's Hospital
2121 Hughes Drive
Harris, McIntosh Tower, Floor E
Toledo, OH 43606
419-291-1115
jeffrey.blumer@utoledo.edu

Medical Monitor

Soju Chang, M.D.
Medical Officer
DMID, NIAID, NIH
6610 Rockledge Drive
Bethesda, MD 20892-6603
301-402-8176
changsoju@niaid.nih.gov

Clinical Project Managers	Tena Knudsen, RN<br>Division of Microbiology and Infectious Diseases<br>National Institute of Allergy and Infectious Diseases<br>National Institutes of Health<br>6610 Rockledge Drive<br>Bethesda, MD 20892-6603<br>301-451-3747<br>knudsen@mail.nih.gov
Medical Officer/Scientific Lead	Mamodikoe Makhene, MD<br>Respiratory Diseases Branch<br>Division of Microbiology and Infectious Diseases<br>National Institute of Allergy and Infectious Diseases<br>National Institutes of Health<br>6610 Rockledge Drive<br>Bethesda, MD 20892-6603<br>301-402-8544<br>mmakhene@niaid.nih.gov
Independent Safety Monitor	Jennifer Hanrahan, DO<br>Division of Infectious Disease<br>MetroHealth Medical Center<br>2500 MetroHealth Drive<br>Cleveland, OH 44109<br>216-778-7828<br>jhanrahan@metrohealth.org
Statistician	Dr. Howard M. Proskin<br>Howard M. Proskin & Associates<br>300 Red Creek Drive, Suite 220<br>Rochester, NY 14623<br>585-359-2420<br>hproskin@hmproskin.com

Data Management	Hector Robertson<br>Clinical Research Management, Inc.<br>1265 Ridge Road, Suite A<br>Hinckley, OH 44233<br>330-278-2343<br>hrobertson@clinicalrm.com
Pharmaceutical/Biotech Contact	Dan Everitt, MD Global Alliance for TB Drug Development<br>40 Wall Street<br>24th Floor<br>New York, NY 10005<br>212-227-7540<br>dan.everitt@tballiance.org
Study Agent Repository	Richard Schrock<br>Fisher BioServices<br>14665 Rothgeb Drive<br>Rockville, MD 20850<br>301-424-6453<br>ricky.schrock@thermofisher.com

2 BACKGROUND INFORMATION AND SCIENTIFIC RATIONALE

2.1 Background Information

TMC207 is a new agent being developed for tuberculosis (TB) treatment. As detailed in the Investigator's Brochure (Tibotec Pharmaceuticals, 2010), TMC207 is a diarylquinoline investigational compound that offers a novel mechanism of anti-TB action by specifically inhibiting mycobacterial adenosine triphosphate (ATP) synthase (Koul, 2007). *In vitro*, TMC207 potently inhibits both drug-sensitive and drug-resistant *M. tuberculosis* isolates (Andries, 2005; Huitric, 2007), and is also bactericidal against non-replicating tubercle bacilli (Koul, 2008). In the murine model of TB, TMC207 is as active as the triple combination of isoniazid (INH), rifampin (RMP), and pyrazinamide (PZA) (Koul, 2007), while addition of TMC207 to this drug regimen results in accelerated clearance of bacilli (Investigator's Brochure, Tibotec Pharmaceuticals, 2010) and synergistic interaction with PZA (Ibrahim, 2007).

Although some progress has been made in recent years in controlling TB globally, TB has remained a persistent problem in resource-limited settings (RLS) of Africa and Asia. TB is currently one of the top three causes of infectious deaths, and there is more TB in the world today than at any other time in history. The current first-line anti-tuberculosis agents have been in use for over 20 years and are relatively ineffective in controlling TB as a public health problem, in part, related to the complexity and efficacy of these regimens, host factors (especially with HIV co-infection), and drug adherence and resistance issues. Although the current regimens and drugs have been very successful in controlled clinical trials, successful treatment requires a minimum of six months. This, plus side effects, result in poor drug adherence, which is particularly likely to occur after the second month of treatment. The full application of directly observed therapy (DOTS) is becoming more and more difficult in RLS that also have the highest burden of TB infection and HIV infection. As a result of poor treatment adherence, drug resistance is becoming more common and fears of an epidemic with virtually untreatable strains of TB are growing. Since the discovery of the rifamycins and their introduction into standard anti-tuberculosis regimens, very few new classes of drugs have been evaluated with a view to their registration as anti-tuberculosis agents (Corbett, 2003).

Following the declaration of TB as a global emergency by the World Health Organization (WHO) in 1993, there has been a resurgence of efforts to develop improved TB therapies and several promising new agents are presently in or approaching clinical evaluation. New agents are desperately needed to shorten treatment to a duration more easily managed by patients and public health services, to provide improved treatment for the growing numbers of patients suffering

from tuberculosis including drug resistant strains, to shorten the treatment of latent TB infection, and to facilitate co-administration of effective TB and HIV treatments.

2.2 Scientific Rationale

Rifabutin is an inducer of CYP3A4 while rifampin is an inducer of a variety of enzymes including the major drug metabolizing cytochrome P450 enzymes CYP3A4, CYP1A2, CYP2C9, and CYP2D6. Other proteins induced by rifampin are glucuronyl transferase and p-glycoprotein. CYP3A4 has an important role in the overall elimination of TMC207. Both rifampin and rifabutin are recognized to cause clinically important drug-drug interactions. The relative ranking of commonly used rifamycins in terms of CYP3A4 enzyme induction potential is rifampin > rifapentine > rifabutin (Li, 1997).

This study is a follow-up to an earlier study, which investigated the relative effects of rifampin and rifapentine on the pharmacokinetics of TMC207 (TMC207-CL-002). In the earlier study, TMC207 (400mg) was administered as a single dose, followed by a four-week washout period to two cohorts of subjects who were randomized to receive either rifampin (600mg once daily) or rifapentine (600mg once daily). Rifampin or rifapentine was then administered for 10 days, 9 days alone (prior to the second dose of TMC207) and then together with TMC207 (400mg) on Day 10 with continued dosing alone for an additional 13 days. Both rifampin and rifapentine reduced TMC207 to a similar extent with overall exposure decreasing by approximately 50%. The present study will determine, for the first time, the relative effect of rifabutin and rifampin on the pharmacokinetics of TMC207.

In this study, the study treatments will consist of rifabutin 300mg once daily or rifampin 600mg once daily. One study treatment will be administered to each of two cohorts over the same total time period. This includes an initial 10-day period prior to TMC207 administration, and an additional 12 days after the administration of the single dose of TMC207. The extended dosing time-period for both inducers is designed to ensure maximum induction of CYP3A4 (Grimm, 2009). The rifampin dose regimen is a standard regime for tuberculosis treatment. The rifabutin 300mg once daily dosing regimen to be employed in this study is the regimen currently recommended by the Centers for Disease Control and Prevention (CDC) for the treatment of tuberculosis in HIV-infected patients (http://www.cdc.gov/tb/publications/guidelines /TB_HIV_Drugs/Table3.htm). The relative extent of the interaction between rifampin and rifabutin with TMC207 will help guide the selection of which of the two rifamycins is more suitable to be co-administered with TMC207 in subsequent studies. The 400-mg dose of TMC207 is the highest dose considered of interest in the TMC207 development program for drug-sensitive TB. Healthy volunteers are being used in this study due to concerns about

administering potentially sub-optimal therapy to TB-infected patients. A single-dose sequential design is being used to minimize both potential exposure in volunteers and to remove uncertainty as to when enzyme activity returns to baseline levels after administration of the inducer has stopped, which is critical information for implementing a valid cross-over design.

2.3 Preclinical Studies

Below is summarized information regarding *in vitro* and *in vivo* preclinical studies involving TMC207 (divided into "Microbiology" and "Non-clinical Safety Studies"). Full details of the preclinical studies are provided in the current Investigator's Brochure (Tibotec Pharmaceuticals, 2010).

Microbiology

In vitro studies have demonstrated that the range of minimum inhibitory concentrations (MICs) for *M. tuberculosis* H37Rv, the international reference strain, and six fully drug-susceptible clinical isolates was 0.030 to 0.120 µg/mL, which is lower than MICs associated with isoniazid and rifampin. The activity of TMC207 appears to be specific for mycobacteria, as the MICs for non-mycobacteria were at least 500-fold higher. The activity of the main metabolite of TMC207, M2, was determined against *M. tuberculosis* H37Rv in both solid and liquid media, and its MIC was found to be 0.1 µg/mL. This MIC shows that M2 is active against *M. tuberculosis* but 3 – 6 times less active than the parent compound TMC207 (MIC of 0.015 – 0.025). TMC207 demonstrated similar *in vitro* efficacy against *M. tuberculosis* clinical isolates resistant to various known anti-TB drugs (INH, RIF, PZA, streptomycin, ethambutol, or fluoroquinolones). As expected, from the lack of cross-resistance with currently used anti-TB agents, TMC207 retained activity against MDR-TB isolates.

In the established murine model of susceptible TB, TMC207 on its own was as active as the standard WHO regimen RMP-INH-PZA, rifampin, isoniazid, and pyrazinamide. Furthermore, when added to RMP-INH-PZA or INH-PZA or RMP-PZA, the lungs of mice harboring 5.94 $\log_{10}$ colony forming unit (CFU) at the start of treatment became culture negative after just two months of treatment (Huitric, 2007). The addition of TMC207 to combinations for treatment of drug-susceptible and MDR-TB appears to accelerate bacterial clearance and may have potential to shorten the duration of TB chemotherapy.

Non-clinical Safety Studies

In single dose toxicity studies there were no mortalities following oral doses of up to 200 mg/kg

in mice and rats. Repeated dose toxicity studies were performed up to three months in mice, rats, and dogs, six months in rats, and nine months in dogs. Recovery was studied in rats and dogs. After single oral administration in rat, mouse, dog, and monkey, absorption of TMC207 was rather rapid. Oral bioavailability was approximately 70% to 80% in mice and rats and 40% in dogs and monkeys. The plasma concentration profiles of TMC207 showed a multi-phasic decline with a long terminal elimination half-life ($t_{1/2}$) ranging from 1 – 3 days in mice to 40 days in dogs. In this respect, intermittent dosing was much better tolerated in dogs, despite a similar exposure as after daily dosing. Intermittent dosing resulted in lower tissue levels of TMC207 and M2. Cessation of treatment or lowering of a toxic dose resulted in clear indications for recovery despite the long half-life and extensive tissue distribution. The dose was slowly excreted in urine and feces after oral administration of 14C-labeled TMC207 to rats, dogs, and monkeys, most of the dose being recovered in feces.

After repeated oral administration, the systemic exposure to the *N*-desmethyl metabolite M2 in mice (AUC) was 2 – 7 times higher than that to unchanged TMC207. In rats and dogs, the exposure to M2 was either comparable or up to two times lower than exposure to TMC207. The plasma levels of TMC207 and M2 increased slowly upon repeated dosing, and steady-state concentrations were not yet reached at one month. The plasma levels of the two compounds increased more or less dose proportionally at low doses and less than dose proportionally at high doses.

Tissue uptake was low in the brain after single oral administration of 14C-labeled TMC207 to pigmented male rats and monkeys. High tissue concentrations were associated with the adrenal gland, lung, spleen, and liver. The decline of the concentrations of total radioactive substances in most tissues was parallel to the plasma concentration decline. Following repeated oral administration of TMC207 to mouse, rat, and dog, tissue concentrations of M2 were generally higher than those of unchanged TMC207. The tissue concentrations of the two compounds increased more than dose proportionally at high dose levels.

In vitro and *in vivo* metabolism studies showed that TMC207 was primarily subjected to oxidative metabolism leading mainly to the N-desmethyl metabolite (M2). Cytochrome P450 3A4 (CYP3A4) is the major CYP form involved *in vitro* in the metabolism of TMC207 and the formation of M2.

In vivo, positive chronotropic effects were seen in the anesthetized guinea pig after intravenous administration but not in the conscious dog. In conscious, telemetered dogs, oral TMC207 had no relevant effects on cardio-hemodynamic and electrocardiogram (ECG) parameters. Respiratory parameters were unaffected by treatment. There were no effects suggestive of

neurological impairment or delayed neurotoxicity in rats.

TMC207 and M2 have cationic amphiphilic drug (CAD) characteristics and therefore induce phospholipidosis, mainly in cells of the mononuclear phagocytic system (MPS) in a variety of organs. It was demonstrated *in vitro* that M2 was a stronger CAD than TMC207. In mice and dogs, a major target organ was the skeletal muscle, in which myopathies were observed in mice, rats, and dogs. In dogs, pronounced myocardial lesions were observed beyond three months of dosing at the highest dose level but other studies only revealed minimal myocardial changes. These findings are consistent with the known ability of CADs to cause myopathies, which tends to occur only after prolonged dosing in humans.

Other noteworthy changes in preclinical studies are as follows. Inflammatory changes in the pancreas were noted at the highest dose level in one dog study after three months of daily dosing and in another dog study only after six months of dosing. The relevance of this finding currently remains unclear. Degenerative lesions have been observed in the fundus of the stomach in mice and dogs. Once established, these lesions stabilized and did not progress. No stomach alterations have been observed in the rat. Pancreatic changes were observed in the dog with normal values in amylase, lipase, and trypsinlike immunoreactivity. Testicular changes (e.g., chronic inflammation, atrophy) were noted in one dog study after six months of dosing but not in another six-month dog study, nor in any other species. The changes were possibly due to the occurrence of phospholipidosis in the Sertoli cells after prolonged dosing, but this remains to be proven. Hepatotoxicity was observed at higher dose levels. In different rat studies it was determined that this was related to a total weekly dose above 100 mg/kg, irrespective of the number of administrations. At dose levels that were associated with overt toxicity, an increase in peripheral neutrophils and neutrophils in tissues were observed.

Corneal eye lesions and intolerance to bright light were observed at the highest dose level after two and three months of dosing in dogs. These changes improved upon continuous dosing. Ophthalmological changes (myopathies) have been described with other CAD drugs, but it remains to be elucidated whether the observed lesions with TMC207 are of a similar nature.

Mice showed high concentrations of the M2 metabolite in plasma, relative to TMC207. Direct dosing of the metabolite showed that the toxicity observed in mice was probably related to M2. It cannot be excluded that this might be the case in other species as well. Furthermore, in *in vitro* experiments, M2 was cytotoxic at lower concentrations than TMC207. In rats and dogs, the exposure to M2 is generally similar to the exposure to TMC207, while in mice M2 exposure is about

In the pre- and post-natal development study in rats, there was no effect of maternal treatment with TMC207 on the attainment of sexual maturity, behavioral development, mating performance, fertility, or reproductive capacity of the F1 offspring. Fetal body weight decreases were observed during lactation and were attributed to pup exposure to TMC207 via the milk. In juvenile rats treated on Days 24 to 60 of age, no effects were seen, there was no new target organ toxicity, and the safety profile was the same as in adult rats.

2.4 Phase 1 and 2 Trials

Based on animal studies, human trials should have dose levels that are in a range associated with linear plasma kinetics and should result in trough levels that do not exceed 7µg/mL. To date, eight Phase I trials in healthy subjects, one short-term Phase IIa and one short-term Phase IIb trial in treatment-naïve patients with sputum smear-positive pulmonary TB have been conducted. One long-term Phase II trial consisting of two different stages has been conducted in patients infected with newly diagnosed sputum smear-positive pulmonary MDR-TB. The principal findings of these trials are summarized below. For more detailed information, please refer to the Investigator's Brochure (Tibotec Pharmaceuticals, 2010) for TMC207. Additionally, a dose-ranging extended early bactericidal activity study has recently completed the treatment and follow-up periods but data are pending. For the Phase I and II trials, except for cutaneous erythema, there were no AEs of special interest (other than those discussed below) that occurred in three or more subjects during treatment with TMC207 co-administered with or without other drugs.

Phase I

The Phase I trials have provided a basic understanding of TMC207's pharmacokinetic characteristics, drug-drug interaction potential, and short-term safety/tolerability profile in 217 healthy subjects. Summaries of available pharmacokinetic data available for TMC207 and its major metabolite M2 are included in Appendices D and E, respectively.

TMC207 was well absorbed with time to reach the maximum plasma concentration (t_{max}) at approximately five hours post-dose. The maximum plasma concentration (C_{max}) and AUC increased proportionally up to the highest doses studied (700mg single dose). Accumulation from Day 1 to Day 14 was approximately two-fold expressed as increase in AUC, while trough concentrations increased up to 3.5-fold after multiple dosing of 400mg. The pharmacokinetics of TMC207 were comparable between healthy subjects and subjects with pulmonary TB. The average $t_{1/2}$ terms of TMC207 and M2 were estimated at 5.5 months and 5.3 months, respectively. Administration of TMC207 as the tablet formulation with food increased the relative bioavailability (by 95%) compared to administration without food.

In the Phase I trials, TMC207 was generally safe and well tolerated. In the overall Phase I safety database (pooled results of all Phase I trials except C110, C111, and C117), the most common adverse events (AEs), irrespective of causality, in the TMC207 dose groups were headache (28.3%), postural dizziness (6.9%), and hyperuricemia (6.2%). During the C110 trial, the most frequently reported AEs were diarrhea (43.8%) and headache (25.0%). During the C111 trial, the most frequently reported AEs, irrespective of causality, were skin laceration and headache under fed conditions (each in 2 [15.4%] subjects), and headache and GGT increased under fasted conditions (each in 2 [13.3%] subjects). The C117 trial had three Phases: during Phase 1 (single dose TMC207 alone), only 2 (12.5%) subjects were reported with an AE (upper respiratory tract infection and diarrhea). During Phase 3 (single-dose TMC207 in combination with nevirapine (NVP, an HIV non-nucleoside reverse transcriptase inhibitor), the most frequently reported AEs were nasopharyngitis (in 4 [25.0%] subjects) and headache (in 3 [18.8%] subjects). All AEs reported during Phase 3 had a similar incidence during Phase 2 (NVP alone), apart from vomiting, which was reported in 2 subjects during Phase 3 but not reported during Phase 2.

No deaths occurred in Phase I trials. Apart from one HIV-1 infected subject who experienced two serious adverse events (SAEs) (grade 3 diarrhea and dehydration; both SAEs were considered not to be related to TMC207, NVP, or nucleoside/nucleotide reverse transcriptase inhibitors (NRTIs) therapy but to be related to HIV), no other SAEs occurred in Phase I trials. Three subjects withdrew due to the following events: a moderate urinary tract infection; pharyngolaryngeal pain and pyrexia; and a grade 3 lipase increase. The lipase increase occurred in three patients in different Groups of two Phase I bioavailability trials. For one subject a grade 3 increase in amylase was already reported predose of the first session (oral solution under fed conditions). Also predose of the first session a grade 3 lipase increase was reported that lasted until Day 11 of that session. TMC207 was well absorbed with a median time to reach the maximum plasma concentration (T_{max}) of about 5 hours after dosing. The maximum plasma concentration (C_{max}) and area under the plasma concentration-time curve (AUC) increased proportionally up to the highest doses studied (700-mg single dose and 400-mg multiple daily doses). Accumulation from Day 1 to Day 14 was approximately 2-fold expressed as an increase in AUC, while trough concentrations increased up to 3.5 fold. The pharmacokinetics of TMC207 in subjects with pulmonary TB were comparable to those in healthy subjects. The average terminal elimination half-lives ($t_{1/2,term}$) of TMC207 and its *N*-monodesmethyl metabolite (M2) were about 132 and 112 days, respectively.

Administration of TMC207 as the tablet formulation with food increased the relative bioavailability (by 95%) as compared to administration without food.

Drug-drug interaction studies confirmed the role of cytochrome P450 3A4 (CYP3A4) in the metabolism of TMC207 to M2. A study with RMP (a CYP3A4 inducer) showed that the exposure (AUC_{336h}) to TMC207 as well as to M2 was significantly reduced (by 52% and 25%, respectively). Furthermore, coadministration of ketoconazole or lopinavir/ritonavir (LPV/rtit) (CYP3A4 inhibitors) increased the systemic exposure to TMC207 without a significant effect on M2. A drug-drug interaction study with the combination of INH/PZA showed a reduction in the exposure (AUC_{24h}) to TMC207 (-13%) after five days of co-administration, while exposure to M2 increased (+30%). TMC207 increased exposure (AUC_{24h}) to both INH and PZA (≤8%) which is not considered clinically relevant. Results from a didanosine trial with NVP in HIV-1 infected subjects demonstrated that steady-state NVP did not significantly influence the single-dose pharmacokinetics of TMC207 or M2. Furthermore, single-dose TMC207 did not influence the exposure to NVP.

Phase II

The Phase II trials provided a first insight on the antibacterial activity of TMC207 in humans.

One short-term Phase IIa trial (Rustomjee, 2008) in 75 individuals with sputum-smear positive pulmonary TB was conducted. The treatment duration was seven days with doses of 25, 100, or 400mg TMC207 q.d. (45 subjects in three treatment groups) or R or H (30 subjects in two control groups). In this study, efficacy (bactericidal activity) was measured by change in the amount of bacilli in the sputum, estimated by changes over time in the number of CFU. The $\log_{10}$ of the CFU counts, the changes from baseline in $\log_{10}$ sputum CFU counts ($\log_{10}$ fall) as well as the average daily change in $\log_{10}$ sputum CFU counts (Early Bactericidal Activity (EBA)), over a period of seven days, were derived.

Over a period of the first two days' treatment, mean values for EBA were 0.01, -0.10, and 0.02 $\log_{10}$ CFU/day for the TMC207 25-mg, 100-mg, and 400-mg groups, respectively. Corresponding values for the control treatments were -0.44 $\log_{10}$ CFU/day for the rifampin group and -0.28 $\log_{10}$ CFU/day for the isoniazid group. By Day 7, changes from baseline in $\log_{10}$ sputum CFU count were -0.04, -0.26, and -0.77 for the TMC207 25-mg, 100-mg, and 400-mg groups, respectively. For the control treatments, the changes versus baseline on Day 7 were -1.70 and -1.88 for the rifampin and isoniazid groups, respectively. For the control treatments, the changes versus baseline on Day 2 were -0.88 and -0.57 for the rifampin and isoniazid groups, respectively. Note that the Day 2 changes for the isoniazid group were influenced by two outliers. All subjects started standard TB therapy after collection of the overnight sputum specimen on Day 7.

There seems to be a delay in onset of response in bactericidal activity for the 400-mg TMC207 treatment group, in that the change in $\log_{10}$ CFU counts for subjects on TMC207 400mg were only evident from Day 4 onward. The lower doses (25mg and 100mg) did not show clinically relevant changes during the seven days of treatment. The reason for this delayed onset of bactericidal activity is not yet clear, but it may be related to the pharmacodynamic characteristics of the TMC207 compound or its mode of action on mycobacteria (energy depletion). This may also be due to suboptimal plasma concentrations and the expected average plasma concentration not being reached in the lowest dose group.

The addition of TMC207 to a 5-drug MDR-TB treatment regimen in an ongoing Phase IIb trial resulted in significantly shorter time to culture conversion compared to placebo. During the eight week treatment in Stage 1 of this trial, 10 of 21 (47.6%) subjects in the TMC207 group became culture negative in the *Mycobacteria* Growth Indicator Tube (MGIT) compared to 2 of 23 (8.7%) subjects in the placebo (background regimen only) group. At Week 24 in Stage 1, after eight weeks of investigational treatment and 24 weeks of background treatment, 81.0% of subjects in the TMC207 group and 65.2% of subjects in the placebo group showed treatment success, i.e., completed Week 24 and were MGIT negative at this time point. The time point from which all evaluable subjects had negative serial sputum colony count (SSCC) cultures was Week 4 for TMC207-treated subjects and Week 12 for placebo-treated subjects. No differences in exposure to TMC207 and M2 in plasma or sputum between subjects with or without culture conversion were observed. The selected dosing regimen (400mg daily for the first two weeks and 200mg three times weekly for the following six weeks) successfully controlled accumulation of TMC207 and M2 in plasma, with most subjects achieving average concentrations above the targeted 600 ng/mL throughout the dosing period. The results of Stage 1 supported the extension of this dosing regimen up to 24 weeks of treatment in Stage 2 of the trial (Diacon, 2009).

Recently, Stage 2 (24-week) data of the Phase IIb trial C208 became available. At the end of the 24-week treatment in Stage 2, 79% of subjects in the TMC207 group became MGIT culture negative compared to 58% of subjects in the background regimen only group. In the first stage of this trial, 47 subjects were enrolled. TMC207 or placebo was administered for 8 weeks on top of a background regimen (BR) (400 mg TMC207 q.d. for 2 weeks followed by 200 mg TMC207 three times/week for six weeks). After the eight week double-blind treatment phase, subjects continued to receive their MDR-TB treatment. They were followed for safety, tolerability, pharmacokinetics, and efficacy for 96 weeks after receiving their last dose of TMC207 or placebo. In Stage 2 of this trial, 161 new subjects with newly diagnosed sputum smear positive pulmonary MDR-TB infection were randomized to receive either TMC207 (400 mg q.d. for the first two weeks and 200 mg three times per week. for the following 22 weeks) or placebo for 24 weeks in combination with MDR-TB treatment for 18 to 24 months. Subjects were then followed

up for 96 weeks after receiving the last dose of TMC207 or placebo. The primary efficacy analysis was performed on all Stage 2 subjects when they had completed the 24-week double-blind treatment period with TMC207 or placebo (or had discontinued earlier).

In the Phase IIa trial, apart from a suggestion of more QTcF prolongation in the TMC207 400-mg group, TMC207 was generally safe and well tolerated. The significance of this QTcF difference is unknown, but there were no cardiovascular AEs reported in either treatment group during the investigational treatment period. The most commonly reported AE was hemoptysis. Two subjects of the TMC207 400-mg group died during the follow-up period.

These were a 25-year old female who died due to two grade 4 SAEs (retroviral infection and pulmonary TB), and a 41-year-old male, who was prematurely withdrawn from trial due to a positive urinalysis test for cannabinoids and who died due to an episode of massive hemoptysis.

There were no other SAEs reported in the TMC207 group. During the 8-week investigational treatment period in Stage 1 of the Phase IIb trial the most common AEs, irrespective of causality, were nausea, arthralgia, hyperuricemia, unilateral deafness, hemoptysis, dizziness, and diarrhea. No deaths were reported during the 8-week investigational treatment of Stage 1, and two subjects experienced other SAEs of which one subject randomized to the TMC207 group experienced a grade 4 diabetic ketoacidosis. One (4.3%) subject in the TMC207 group died during the 96-week BR period: a 33-year-old, HIV-positive, female who experienced grade 4 myocardial infarction that resulted in death on the same day. The event was considered not related to the investigational medication or drugs in the BR and was not related to TB in the opinion of the investigator.

During the 24-week investigational treatment period in Stage 2 of this PhaseIIb trial, the most common AEs, irrespective of causality, were arthralgia, headache, hyperuricemia, nausea, and vomiting. The number of subjects experiencing at least one SAE was comparable in the TMC207 (15.2%) and placebo (12.3%) groups. Three subjects, one (1.2%) on placebo and two (2.5%) on TMC207, died during the study. A 24-year-old female subject with bilateral lung cavitations who was treated with placebo developed massive, fatal hemoptysis, following the 24-week period of study drug administration after the end of treatment, and she died the same day. The AE was considered doubtfully related to the study medication by the investigator. A 54-year-old male subject with cavitation in one lung, treated with TMC207, died of acute alcohol poisoning following the 24-week period of study drug administration. Another subject, an 18-year-old male subject with cavitation in one lung, treated with TMC207, was reported with relapse of MDR-TB following the 24-week period of study drug administration. He died about six months later. The investigators considered both of these deaths not related to the study medication.

The liver, skeletal muscle, heart, pancreas, stomach, and eyes have been identified as target organs in nonclinical toxicology studies. Careful monitoring of these organs will be included in clinical trials. Any abnormalities in the parameters related to these target organs are therefore considered as AEs of special interest. Because other TB compounds with which TMC207 will have to be administered have been associated with rash, skin events are considered AEs of special interest in the TMC207 target population as well.

Recently, a 20 year old female patient who received an unknown amount of TMC207 for treatment of pre-extensively drug resistant tuberculosis experienced adverse events in the form of vertigo, feeling of nausea, and possible drug interaction. These feelings manifested approximately 10 days after initiating TMC207 treatment. TMC207 treatment was withdrawn and the patient recovered within four days. Upon administration of TMC207 for a second time, symptoms reappeared. This patient was also being treated with an unknown dosing regimen of Noxafil (posaconazole) for aspergillosis. The reporter considered the adverse events to be due to a possible drug interaction between TMC207 and posaconazole and permanently discontinued treatment with TMC207. No changes in the patient's posaconazole regimen were made. This report is considered serious (medically significant).

2.5 Rifampin and Rifabutin

Rifampin

Rifampin is an FDA-approved, active, bactericidal anti-tuberculosis drug and is the most "sterilizing" of the current TB drugs (i.e., most effective against the persistent, slowly replicating bacilli). It is also particularly active against the rapidly growing extracellular organisms and also has bactericidal activity intracellularly. It is rapidly absorbed; C_{max} (8 to 20 µg/mL) is reached at 2 hours after a single 600mg oral dose under fasting conditions. With food T_{max} is prolonged to 4 hours post-dose, and C_{max} is reduced by 36%. Rifampin is extensively eliminated by intestinal and hepatic metabolism, and undergoes enterohepatic recirculation. Thirteen (13) to 24% of the dose administered is excreted as unchanged in urine. The terminal half-life ranges from two to five hours. Repeated administration of rifampin increases oral clearance by inducing its metabolism. Autoinduction of rifampin metabolism is characterized by a decrease in AUC and terminal half-life. Steady state conditions are generally achieved after the 6th daily dose of 600mg rifampin. The main concerns associated with the use of rifampin are the adverse effects on the liver and potential for drug interactions.

Rifabutin

Rifabutin is an FDA-approved, semisynthetic ansamycin antibiotic derived from rifamycin S and has been shown to have significant mycobactericidal activity. It is regarded as a less potent enzyme inducer than rifampin and is therefore the preferred rifamycin antibiotic used to treat TB in HIV-infected patients.

Rifabutin is readily absorbed from the gastrointestinal tract with C_{max} 375 ng/mL (141 to 1033 ng/mL) reached 3.3 hours after a single 300-mg oral dose under fasting conditions. With a high fat meal, T_{max} is prolonged, but the overall extent of absorption is unchanged. Plasma protein binding of rifabutin is 85%. It is extensively eliminated by intestinal and hepatic metabolism, and undergoes entero-hepatic recirculation. Renal and biliary excretions each contribute only 5% to the total systemic clearance. The terminal half-life is approximately 45 (range 16 to 69) hours. The rifabutin drug-drug interaction profile is complex. It is an inducer but not an inhibitor of CYP3A4 and thus, induces the clearance of drugs metabolized by CYP3A4. It is also a substrate of CYP3A4 and its exposure is, therefore, reduced by other CYP3A4 enzyme inducers (such as efavirenz) and increased by potent CYP3A4 enzyme inhibitors such as clarithromycin. Repeated administration of rifabutin increases its oral clearance by inducing its own metabolism. Autoinduction of rifabutin metabolism is characterized by a decrease in systemic drug levels of 38%, but its terminal half-life remains unchanged. The main concerns associated with the use of rifabutin are uveitis, neutropenia, and its potential for drug interactions.

2.6 Potential Risks/Benefits

Clinical Safety

In the Phase I trials, TMC207 was generally safe and well tolerated. The most common adverse events (AEs) were headache, nasopharyngitis, postural dizziness, and hyperuricemia. Cases of hyperuricemia in Phase II studies were observed with combination therapy, and hyperuricemia is a known side effect of pyrazinamide. There were no deaths or serious adverse events (SAEs) reported in Phase I trials.

In the short-term Phase IIa trial in 75 treatment-naïve TB-infected subjects, the most commonly reported AE was hemoptysis, a common complication of pulmonary TB. The most commonly reported AE during treatment phases including TMC207 was hemoptysis, reported by one (6%) subject in the 100-mg group and three (21%) subjects in the 400-mg group. All AEs were considered not or doubtfully related to the trial medication, except for two AEs (diarrhea and rash) in the 100-mg TMC207 group and 1 AE (somnolence) in the 400-mg TMC207 group, which were considered possibly related to the study medication. All AEs were grade 1 or 2 in severity, except for one subject in the isoniazid group, with a grade 4 hemoptysis. This event was serious and considered not related to the study medication, but related to TB and led to

premature discontinuation. One additional subject in the isoniazid group prematurely discontinued treatment due to an AE (hemoptysis; grade 3 in severity) that had already started during the screening period.

Two subjects in the TMC207 400-mg group died during the follow-up period. One subject was a 25-year old female who died due to retroviral infection and pulmonary TB, and the other was a 41-year old male who was prematurely withdrawn after 3 days of treatment, started standard TB therapy, and eventually died due to massive hemoptysis.

Changes in QTcF were seen in both the TMC207 and control groups. Average increases of more than 10 ms relative to baseline in QTcF were seen on Day 7 for the TMC207 400-mg group and for both control groups. On Day 7, effects were larger 5 hours post-dose than pre-dose for all treatment groups. Increases by 30 – 60ms in QTcF were observed for one subject in the 100-mg TMC207 group; six subjects in the 400-mg TMC207 group, and for four and two subjects of the rifampin and isoniazid groups, respectively. In addition, one patient in the rifampin group had an increase >60ms in QTcF.

2.6.1 Potential Risks

As noted above, the major end-organ toxicities that have been observed in pre-clinical studies included effects on mononuclear phagocytic system, skeletal and cardiac muscle, testes, stomach, liver, pancreas, and eye (cornea). This clinical trial will include careful assessment of relevant biomarkers for these organs/tissues. In clinical studies to date, headache, nasal congestion, and postural dizziness were observed, but all were grade 1 or 2 in severity. These possible adverse events will be monitored serially in this study. Clearly, there is potential for TMC207 to interact with oral contraceptives, and the safety of this compound has not been established in pregnant or lactating women. As indicated below in Section 5.1, women will be enrolled in this study only if they have undergone hysterectomy, a sterilizing procedure, or if they agree to use two forms of contraception. Long term risks to the study population are not known. However, no long term toxicities have been recognized in humans treated with this drug to date. Further, subjects in this protocol will only receive two single doses of TMC207. The potential for drug-drug interactions (specifically with rifamycins metabolized through CYP3A4) is the major focus of this study.

2.6.2 Known Potential Benefits

The trial has no benefit for participating subjects. However, the knowledge gained will inform the design of future therapeutic trials. Novel compounds in a new class of anti-tuberculosis

agents are critical to develop as the occurrence of tuberculosis remains high in endemic areas with increasing co-infection in HIV-infected individuals.

2.6.3 Risk/Benefit Ratio

To reduce risks in this study, subjects will be monitored closely with serial examinations, laboratory tests, and electrocardiograms (ECGs). Because of the potential issue of corneal abnormalities from TMC207 as observed in animal models and uveitis seen with rifabutin, serial dilated retinal exams will be performed throughout the study by retinal specialists as detailed below in Section 7. This evaluation will include a retinal exam and slit lamp examination. Furthermore, subjects will be educated about possible symptoms of corneal defects and uveitis (loss of vision, eye pain, and visual blurring) to be reported to study staff for immediate ophthalmologic evaluation. In the event of possible allergic reactions (including anaphylaxis), treatment and resuscitation equipment will be available at all times at the study site. Data from prior phase 1 and 2 trials of TMC207 demonstrate the safety and tolerability of this drug.

Once discharged from the research setting, each subject will be provided with clear written instructions of what to do and who to contact in case of side effects or other health concerns.

In addition to the above risks, the risk of volunteering for this study includes a potential failure to protect the subject’s private health and other information through an accidental breach of confidentiality. Safeguards will be taken to protect health and study information. Research and medical records associated with this protocol are subject to the provisions of the Privacy Act. All data and medical information obtained about each subject will be considered privileged and held in confidence. The procedures to protect against the breach of confidentiality include the following: 1) No subject will be identified by name in any published report or presentation of the results. 2) Each subject will be assigned a study number, and this identifier will be used on the study forms. 3) Only study investigators, researchers at the National Institute of Allergy and Infectious Diseases (NIAID), the Food and Drug Administration (FDA), and other authorized study personnel will have access to any protected health information (PHI). 4) The electronic transmission of PHI will be done in compliance with NIH and other federal Information Security Guidelines. To comply with these guidelines, PHI will only be transmitted via a secure line or in an encrypted fashion. This risk of breach of confidentiality and the associated safeguards to protect confidentiality will be defined in each informed consent document.

3 STUDY OBJECTIVES

Primary:

- To evaluate the effect of repeated doses of 300mg rifabutin or 600mg rifampin on the pharmacokinetics of TMC207(and its M2 metabolite) given as a single dose in healthy subjects
- To evaluate the safety and tolerability of TMC207 (and its M2 metabolite) when given with 300mg rifabutin or 600mg rifampin

Exploratory:

- To evaluate the cell-associated levels of TMC207 and its M2 metabolite in peripheral blood mononuclear cells (PBMCs) after single and multiple doses in healthy subjects
- To evaluate whole blood bactericidal assays in subjects receiving TMC207 alone or in combination with rifampin or rifabutin.

Hypothesis:

Rifabutin affects the pharmacokinetics of TMC207, as measured by effects on C_{max} and $AUC_{(0-t)}$, to a lesser degree than observed for rifampin.

4 STUDY DESIGN

This will be a Phase l, open label, single-center, two-period, single-sequence study to evaluate the effect of repeated doses of rifabutin or rifampin on the pharmacokinetics of a single dose of TMC207.

Table 2: Study design

Treatment	Number of subjects	Dose Regimen	Number of Tablets/Capsules
**Group 1** Period 1	16	**TMC207:** 400mg single dose on Day 1	4 tablets of TMC207 per intake (TMC207 100mg per tablet)
Period 2		**TMC207:** 400mg single dose on Day 29<br><br>**Rifabutin:** 300mg on Days 20 to 41	4 tablets of TMC207 per intake (TMC207 100mg per tablet)<br><br>2 capsules of Rifabutin per intake (Rifabutin 150mg per capsule)
**Group 2** Period 1	16	**TMC207:** 400mg single dose on Day 1	4 tablets of TMC207 per intake (TMC207 100mg per tablet)
Period 2		**TMC207:** 400mg single dose on Day 29<br><br>**Rifampin:** 600mg on Days 20 to 41	4 tablets of TMC207 per intake (TMC207 100mg per tablet)<br><br>4 capsules of Rifampin per intake (Rifampin 150mg per capsule)

Thirty-two subjects (16 in each group) will be enrolled; both males and females are eligible for enrollment.

4.1 Study Outcome Measures

Pharmacokinetic profiles over 336 hours will be determined for TMC207 and M2 after administration of TMC207 400mg alone (Study Day 1, Groups 1 and 2), and in combination with steady-state rifabutin 300mg (Study Day 29, Group 1) or rifampin 600mg (Study Day 29, Group 2).

The following primary PK parameters will be estimated for TMC207 and M2:

- C_{max}
- $AUC_{(0-t)}$
- T_{max}
- $T_{1/2}$
- Kel
- ratios of $C_{max(test)}/C_{max(reference)}$, $AUC_{(0-t, test)}/AUC_{(0-t, reference)}$

Note: Test = TMC207 in combination with rifabutin or rifampin; reference = TMC207 alone

Area under the plasma concentration-time curve from time zero to τ ($AUC_{0-\tau}$) will be estimated by integration. The infinity extrapolation for single dose data will be the last measurable concentration (C_τ) divided by λ_Z (C_τ/ λ_Z). Data will also be modeled in a fully parametric sense. Linear models will be discriminated by the Akaike Information Criterion. Michaelis-Menten models will be discriminated by the likelihood ratio test. Half life (if the system is linear) will be estimated by taking at least the last four measurable time points and fitting a single exponential model to the data. The ADAPT V package of programs of D'Argenio and Schumitzky will be employed. A maximum likelihood estimator will be employed. Inverse observation variance will be employed as the weighting scheme.

If necessary, population pharmacokinetic modeling will be employed. The Big NPAG Program of Leary, Jelliffe, Schumitzky and VanGuilder will be employed. Again, multiple models will be evaluated. An inverse variance weighting scheme will be employed. Adaptive γ will also be employed as part of the weighting function. If appropriate, a demographic model will be built.

The frequency and severity of adverse events (AEs, both solicited and unsolicited) will be assessed on a continual basis throughout the study via safety assessments, observation, direct participant reporting, and specific AE inquiry ("How do you feel" or "HDYF" questions) at various points during the study. Physical examinations, eye examination with fundoscopy and slit lamp exams, vital signs, ECGs, hematology, serum chemistry, coagulation, and urinalysis will also be used to assess safety and tolerability.

4.1.1 Primary Outcome Measures

The pharmacokinetics effect of repeated doses of 300mg rifabutin or 600mg rifampin on a single dose of TMC207 and its M2 metabolite in healthy subjects will be determined by measuring plasma levels at multiple time points.

The safety and tolerability of TMC207 when given as a single dose in combination with rifabutin or rifampin will be evaluated by serial assessment of the occurrence of solicited and unsolicited adverse events, including symptoms, physical findings, laboratory testing (hematology, chemistries, urinalysis), ophthalmologic exams, and ECG changes.

5 STUDY ENROLLMENT AND WITHDRAWAL

Thirty-two healthy male and female participants, ages 18 – 45 years will be enrolled in two groups. It is estimated that 96 subjects will need to be screened (3:1, screened: eligible) to enroll 32 subjects. Subjects will be recruited through customary IRB-approved approaches. Children and pregnant or breast-feeding women will not be enrolled. Prisoners and other vulnerable groups will also not be enrolled.

Screening will begin with a brief IRB-approved telephone screen. Information about the study will be presented to potential subjects. The telephone script will include the inclusion/exclusion criteria. Appointments will be made for subjects who are interested in the study at the University Hospitals Case Medical Center (UHCMC) Dahms Clinical Research Unit (part of the Case Western Reserve University CTSA) for further screening procedures and protocol-specific information.

Study retention strategies will include education and explanations of the study schedule and procedures during enrollment. In addition, reimbursements will be dispersed at specified time points during the study with the amount contingent on completion of study procedures. Participants will be reminded before visits, and the clinical research study staff will contact participants who miss appointments to determine if they wish to return for further evaluation.

5.1 Inclusion Criteria

Potential subjects must fulfill all of the following inclusion criteria at baseline to be eligible for participation in the study:

1. Aged between 18 and 45 years, extremes included.
2. Non-tobacco/nicotine using (at least 3 months prior to screening).
3. Body Mass Index (BMI, weight in kg divided by the square of height in meters) of 18.0 to <35.0 kg/m^2.
4. Informed Consent Form (ICF) signed voluntarily before the first trial-related activity.
5. Able to comply with protocol requirements.
6. Healthy on the basis of a medical evaluation or history that reveals the absence of any clinically relevant abnormality and includes a physical examination, medical history, electrocardiogram (ECG), vital signs, ophthalmologic exam, the results of blood biochemistry, and hematology tests, and a urinalysis carried out at screening (See Section 7.2).
7. Subjects will be enrolled in this study only if they have undergone vasectomy/complete hysterectomy, tubal ligation, or other sterilizing procedure, or the subject is a post-menopausal woman for more than two years, or if sexually active subjects agree to use two

of the following forms of adequate contraception during the study and for 12 weeks after the final dose: abstinence, condoms with or without spermicide gel, diaphragm with spermicide gel, hormonal or non-hormonal intrauterine device, oral contraceptive pills, and depot progesterone injections. If a subject is usually not sexually active but becomes active, the subject and his or her partner must use two of the listed contraceptive methods.

5.2 Exclusion Criteria

Potential subjects must meet none of the following exclusion criteria to be eligible for participation in the study:

Medical History

1. History or evidence of current use of alcohol, barbiturate, amphetamine, recreational, or narcotic drug use, which in the investigator's opinion would compromise subject's safety and/or compliance with the trial procedures.
2. Any clinically significant (as deemed by the Principal Investigator) history of acute illness (resolved within four weeks of screening), asthma, or presence of cardiovascular, pulmonary, hepatic, renal, hematologic, gastrointestinal (including eating disorders), endocrine, metabolic, immunologic, dermatologic, neurologic, psychological, or psychiatric disease.
3. Currently significant diarrhea, gastric stasis, or constipation that in the investigator's opinion could influence drug absorption or bioavailability.
4. Any history of significant skin disease such as, but not limited to, rash or eruptions, drug allergies, food allergy, dermatitis, eczema, psoriasis, or urticaria. Subjects with a history of skin disease may be enrolled into the study after consultation with the Sponsor Medical Monitor.
5. Previously demonstrated clinically significant allergy or hypersensitivity to any of the excipients of the investigational medication administered in this trial (i.e., rifabutin, rifampin, and TMC207).
6. Subjects with QTcB [Bazett correction] interval > 450ms at screening
7. Subjects with any other clinically significant ECG abnormality at screening, such as arrhythmia, ischemia, or evidence of heart failure or with a family history of Long QT Syndrome.
8. History or evidence of ophthalmologic diseases except for routine corrected hyperopia, myopia, and presbyopia.
9. Recent history (within past 30 days) of vertigo/nausea.

Specific Treatments

10. Current use of any azole antifungal agent

11. Use of concomitant medication, including over-the-counter products and dietary supplements, without approval from study staff. Subjects will be treated based on symptom presentation, with the exception of medications that affect p450 and 3a metabolic pathways (refer to the MOP for a list of acceptable medications). During outpatient time periods, subjects will be required to discuss with the study staff and receive approval before self-administering any medication. After gaining approval, subjects will also be asked to record any medication taken during outpatient time periods in a provided log.
12. Participation in an investigational drug trial within 60 days prior to the first intake of trial medication and during the duration of the study.
13. Donation of blood or significant loss of blood within 56 days or plasma donation within 7 days preceding the first intake of trial medication.
14. Having received TMC207 in a previous trial.

Based on Laboratory Abnormalities

15. Positive HIV-1 or HIV-2 test by ELISA at screening.
16. Hepatitis A, B, or C infection (confirmed by hepatitis A antibody IgM, hepatitis B surface antigen, or hepatitis C virus antibody, respectively) at screening.
17. A positive urine drug test at screening. Urine will be tested to check the current use of amphetamines, benzodiazepines, cocaine, cannabinoids, and opioids; along with serum alcohol level.
18. Subjects with the following laboratory abnormalities at screening as defined by the NIH, NIAID, Division of Microbiology and Infectious Diseases (DMID) Adult Toxicity Table (Appendix C) and in accordance with the normal ranges of the clinical laboratory:
    a. Serum creatinine grade 1 or greater (> 1.0 x ULN),
    b. Pancreatic lipase grade 1 or greater (> 1.0 x ULN),
    c. Hemoglobin grade 1 or greater (≤ 10.5 g/dL),
    d. Platelet count grade 1 or greater (≤ 99000/mm^3),
    e. Aspartate aminotransferase (AST) or alanine aminotransferase (ALT) grade 1 or greater (> 1.0 x ULN),
    f. Total bilirubin grade 1 or greater (> 1.0 x ULN),
    g. Creatine kinase grade 1 or greater (>1.0 x ULN),
    h. Troponin grade 1 or greater (.1.0 x UNL), or
    i. Any other toxicity grade 2 or above, including: proteinuria (spot urine) > 1+ and gross hematuria. For the second dose of TMC207, any other toxicity grade 3 or above, including: proteinuria (spot urine) > 1+ and gross hematuria.

All inclusion and no exclusion criteria must be met unless, after discussion between the Principal Investigator and the Sponsor Medical Monitor before subject enrollment, the exception is determined to be of no clinical significance and unlikely to have any significant effect on the results of the study. The exception and date of the decision will need to be approved in writing by the Sponsor and it will be documented in the case report form (CRF). Additionally, at the discretion of the PI, subjects may be excluded for reasons not listed above.

5.2.1 Study Restrictions

Foods and Beverages
Subjects may not consume beverages containing alcohol or quinine (e.g., tonic, bitter lemon, bitter alcoholic beverages containing quinine) between 24 hours before the first intake of TMC207 until the last pharmacokinetic blood sample has been taken in each period. Grapefruit and grapefruit juice are not allowed between 7 days before the first intake of TMC207 until the last pharmacokinetic blood sample has been taken in each period.

Prior and Concomitant Medications
While in the DCRU, subjects will be treated based on symptom presentation, with the exception of medications that affect p450 and 3a metabolic pathways (refer to the MOP for a list of acceptable medications). During outpatient time periods, subjects will be required to contact study staff to get approval before self-administering any medication. After gaining approval, subjects will also be asked to record any medication taken during outpatient time periods in a provided log.

Women on hormonal contraception (pills, patches, and IUDs) are eligible for this study. Female gonadal steroids affect P450 metabolism, including 3A, 2C9, and 1A2. Therefore, we will carefully record date of last menses and the type of contraceptive methods utilized by subjects on the CRF forms at Screening. The date of last menses and the type of contraceptive methods utilized by subjects will be recorded in the source documents thereafter. Women who are post-menopausal and on hormone replacement therapy will also allowed to participate.

Activity
Subjects will not significantly change their normal level of exercise and will refrain from strenuous activities during the duration of the trial.

Extreme exposure to the sun or sunbathing should be avoided, as well as the use of tanning devices (e.g., sun bed, solarium) and topical tanning products from screening until the last trial-related visit. In case sun exposure cannot be avoided, subjects must wear protective clothing and, for sun exposed parts of the skin, potent UVB sun blockers must be used during the aforementioned period.

Donation
Subjects should not donate blood, plasma, platelets, or any other blood components for 180 days following the scheduled completion of the treatment phase of the study or following the last dose of TMC207 in the case of early withdrawal.

Pregnancy
Sexually active women can be enrolled into the study as long as they utilize two adequate forms of contraception as defined in Section 5.1. Since the effects of TMC207 on conception are unknown, women who are postmenopausal for more than two years, post-hysterectomy, or post-surgical sterilization, (i.e., tubal ligation or partial or total hysterectomy) will be allowed into the study. Women who do not meet the above criteria for inclusion in the study may be enrolled if they agree to use two adequate forms of contraception, as described in Section 5.1. A serum pregnancy test will be performed at check in of each study period to confirm that subject is not pregnant.

Heterosexually active men must agree to use two concomitant acceptable methods of contraception (e.g., vasectomy combined with latex condom with spermicide, latex condom with spermicide combined with a female partner who practices an acceptable method of contraception according to the history provided by the subject and as deemed by the investigator) (see Section 5.1 above). This requirement will extend from screening onwards until 90 days following the last dose of TMC207 or 90 days after discontinuation of the trial medication in case of premature discontinuation. In addition, male subjects should not donate sperm 90 days following the last dose of TMC207 or 90 days after discontinuation of the trial medication in case of premature discontinuation. Proof of vasectomy (e.g., a physician note) is required for this to count as an acceptable method of contraception.

For details on the existing data with regard to the reproductive toxicity of TMC207, see the current Investigator's Brochure (Tibotec Pharmaceuticals, 2010).

Other Restrictions
Rifabutin and rifampin can produce a reddish discoloration of bodily fluids such as urine, sweat, tears, and sputum and can stain contact lenses or dentures, so these should not be worn during the study.

5.3 Treatment Assignment Procedures

This Phase I study has two groups of subjects. Group 1 will consist of 16 subjects and Group 2 will also include 16 subjects, with both groups receiving a single dose of 400mg of TMC207 at the beginning of the study. Group 1 will begin rifabutin 300mg orally on Days 20 – 41 while Group 2 will begin rifampin 600mg orally through the same time period. On Day 29, after a four-week washout period for the TMC207, a single oral dose of 400mg of TMC207 will be administered to each group. Subjects in Groups 1 and 2 will be replaced should they withdraw from the study (see Section 5.4).

5.3.1 Randomization Procedures

The randomization list will be generated by the Study Biostatistician and transferred to the study pharmacist prior to the start of Period 2. As necessary, additional recruitment of subjects for Groups 1 and 2 will be considered by the Principal Investigator to replace subjects should they withdraw from the study. The Study Biostatistician will conduct an on-site training session with the pharmacist before the start of the study if needed, and perform a randomization audit at the site with the study pharmacist at the end of each Period.

5.3.2 Blinding Procedures

This is an open label study; blinding is not applicable.

5.4 Withdrawal

Each subject will be encouraged to complete the full course of the intervention assignment and study assessments. However, it is understood that the subject may discontinue study participation at any time for any reason. In the event a subject withdraws prematurely from the confinement period of the study, the same procedures as scheduled for Study Day 2 (or as described in the flow chart in case of dropout other than withdrawal of consent) will be performed at the time of study withdrawal, if possible. In addition, subjects should be encouraged to return to the unit within 28 days after dropout for additional safety tests. The reason for early withdrawal must be documented in the CRF and in the source document. Any subject who withdraws who has received any dose of TMC207 will have toxicity, safety, tolerability, and PK data to the time of withdrawl analyzed and included in final reports as supplemental data. The maximum allowable number of replacements for Groups 1 and 2 is three subjects per Group. Replacements must complete all four crossover periods.

5.4.1 Reasons for Withdrawal

Subjects are free to withdraw from the study at any time for any reason.

Subjects should normally be withdrawn from the trial if a serious adverse event (SAE), assessed to be study drug related by the study physician, occurs. Exceptions must be discussed with the Sponsor.

Subjects **must** be withdrawn from the trial if:

1. They withdraw their consent;
2. The investigator considers it in the best interest of the subject that he or she is withdrawn;

3. They experience a grade 3 or 4 adverse event (AE) or grade 3 or 4 confirmed laboratory toxicity believed to be drug-related (a confirmatory test should be performed preferably within 48 hours after the results have become available in case a grade 3 or grade 4 laboratory abnormality occurs); exceptions are asymptomatic grade 3 pancreatic amylase elevations with no history or concomitant disease of pancreatitis and lymphocytopenia of grade 4 severity which persists for up to six days;
4. They experience grade 3 or 4 toxicities as outlined in Appendix C, believed to be drug-related.

The reason for any subject's discontinuation and the date of withdrawal will be recorded in the subject's CRF. The subject's CRF, which will be completed up to the point of withdrawal, will be retained for the Sponsor. The study report will include reasons for subjects' withdrawals as well as details relevant to the subjects' withdrawals. Any subject withdrawn from the trial prior to completion will undergo all procedures indicated in this protocol as being scheduled to occur at discharge or upon early withdrawal, subject to his/her consent. Any subject withdrawn due to an adverse event (whether serious or non-serious) or any clinically significant abnormal laboratory test value will be evaluated by the Principal Investigator or a monitor (see Key Personnel), and will be treated and followed up until the symptoms or values return to normal or acceptable levels, as judged by the Principal Investigator.

Relevant post-study procedures will be performed, wherever possible, on subjects who elect to withdraw.

5.4.2 Handling of Withdrawal

If a subject is withdrawn from participation in the study at any time at his or her request, at the IRB, DMID, FDA, or Principal Investigator's discretion, the reason(s) for discontinuation shall be documented thoroughly in the source documents and CRFs. All subjects who are withdrawn prematurely will undergo the procedures outlined in the Discharge visit (See Schedule of Events, Appendix A), if possible. If a subject is discontinued because of an adverse event, this event will be followed until it is resolved or the subject is clinically stable and will also be documented in the source documents and CRFs.

Subjects who receive any amount of study product and who subsequently withdraw or are withdrawn from the study will be encouraged to continue Follow-up (with subject's consent) for safety. The subject will be asked for permission to continue scheduled evaluations and complete an end-of study evaluation. If an AE or SAE has occurred, every effort will be made to undertake protocol-specific safety follow-up procedures, and the subjects will be encouraged to receive appropriate care until the signs and symptoms or laboratory toxicities resolve. If the subject withdraws or is withdrawn from the study and the timeline for that cohort permits, the subject

may be replaced. Subjects who withdraw before receiving the first dose of study drug will be replaced. Subjects who withdraw after receiving the first dose of study drug may be replaced at the discretion of the Principal Investigator.

5.5 Termination of the Study

The Principal Investigator and the Phase I Clinical Unit reserve the right to terminate the study, in consultation with DMID and TB Alliance, in the interest of subject welfare. The Sponsor reserves the right to terminate the study at any time for any reason. Additionally, the study may be discontinued at the request of FDA or the IRB. Finally, the subjects may withdraw from the study at any time.

Should study discontinuation be necessary, the Principal Investigator, the Phase I Clinical Unit, DMID, and the TB Alliance will jointly arrange discontinuation procedures and notify the appropriate regulatory authorities and the IRB. In terminating the study, the Sponsor and the Principal Investigator will assure that adequate consideration is given to the protection of the subjects' interests. Enrolled subjects will be processed as withdrawals according to the procedures noted in Section 5.4.

6 INVESTIGATIONAL PRODUCT(S)

6.1 Study Product(s) Description

The investigational medication, TMC207, will be manufactured and provided under the responsibility of Tibotec, Johnson & Johnson Pharmaceutical Research and Development, a division of Janssen Pharmaceutica N.V.

The Chemical Abstracts Service Number is 845533-86-0 for the fumarate salt (tablet form) of TMC207 used in the current protocol.

Structure:

Free Base

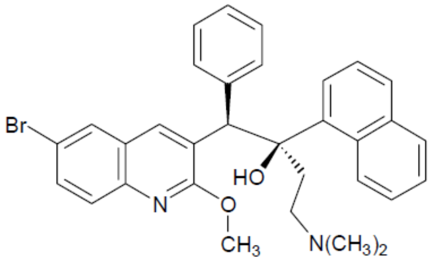

Fumarate Salt

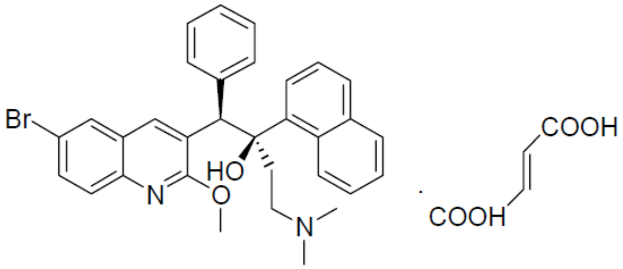

The molecular formula for the tablet (fumarate salt) form of TMC207 used in this protocol is: $C_{32}H_{31}BrN_2O2.C_4H_4O_4$. Its molecular weight is 671.58 Daltons. The chemical name of this form of TMC207 is (1R,2S)-6-bromo-alpha-[2-(dimethylamino)ethyl]-2-methoxy-alpha-1-naphthalenyl-beta-phenyl-3-quinolineethanol (2E)-2-butenedioate(1:1) (salt).

6.1.1 Formulation, Packaging, and Labeling

TMC207 is provided as white to off-white, oval tablets. The complete formulation of TMC207 is provided in the Investigator's Brochure (Tibotec Pharmaceuticals, 2010). TMC207 will be packed per intake and per subject under responsibility of Tibotec. Labels will contain the protocol number, batch or reference number, storage caution statements, dispensing instructions, and 'keep out of reach of children' warning. In addition, the medication will be labeled according to the local regulatory requirements (i.e., "Caution: New Drug Limited by Federal [United States] Law to Investigational Use").

Rifabutin and rifampin will be commercially purchased and labeled for exclusive use in this trial.

The Sponsor will supply sufficient quantities of TMC207 to allow completion of this study. All TMC207 supplied for this study will come from the same lot/batch (or reference number) (within each Phase). The lot number of the rifabutin and rifampin administered will be recorded in the case report form of each subject.

The dose of TMC207 400mg will be administered as four 100-mg tablets. The rifabutin will be administered as two 150-mg capsules for the 300-mg dose. The rifampin 600-mg doses will be administered as four 150-mg capsules.

6.1.2 Acquisition

The TB Alliance will coordinate the shipment of the TMC207 to the drug depot (Fisher). DMID will purchase and supply the rifabutin and rifampin.

6.1.3 Product Storage, Stability, and Expiration

All study medication will be kept securely stored by the site pharmacist/registered dispenser in a secured area with limited access to designated site personnel only. TMC207 will be stored in the supplied containers between 15 and 30°C (59 – 86°F).

Rifabutin and rifampin will be stored as suggested in the manufacturer's Package Insert information.

6.1.4 Preparation/Handling

All study medications are received in tablet form, thus no preparation is required.

6.1.5 Administration

All medication intakes and meals at the unit will be witnessed by the investigator or by the personnel dedicated to the trial and documented in the subject's chart.

If a subject's medication intake is not according to the protocol, the investigator will take the necessary measures to ensure future adherence to the protocol and inform the Sponsor.

Subjects will receive their assigned study drug doses with 240mL of water. Doses will be administered by giving no more than two tablets at a time with a portion of the water until all of the tablets/capsules have been taken. All subjects are required to drink all 240mL of water. On Study Day 29, TMC207 intake should be within five minutes after rifabutin or rifampin intake.

Period 1

Subjects in Groups 1 and 2 will visit the testing facility and medication will be administered as described in the schematic. In Period 1, subjects in both Group 1 and Group 2 will be admitted to the testing facility on Study Day -1. On Study Day 1 subjects having fasted overnight for at least 10 hours will be served a standardized breakfast. This standardized breakfast should be ingested entirely within 30 minutes. TMC207 will be taken within 30 minutes after completion of breakfast with approximately 240mL of water. A standardized lunch (selected by each subject from a menu offered at the DCRU, in accordance with any dietary restrictions) will be served in the testing facility at approximately (but no less than) four hours after intake of TMC207. Intake of water is allowed until two hours before the intake of trial medication and from two hours after intake of trial medication.

Period 2

In Period 2, subjects in Group 1 and Group 2 on Study Days 20 to 28, will be administered rifampin or rifabutin one hour (±15 minutes) prior to breakfast. Subjects in Group 1 and Group 2 will be admitted to the testing facility on Study Day 28 and remain in the unit until Study Day 30.

On Study Day 29, subjects having fasted overnight for at least 10 hours will be served a standardized breakfast which should be ingested entirely within 30 minutes. Rifampin or rifabutin will be taken within 30 minutes after completion of the standardized breakfast followed by TMC207 within 5 minutes of rifampin or rifabutin administration with approximately 240mL water (allowable window for each intake is ±15 minutes, but TMC207 must be given after rifampin/rifabutin). A standardized lunch will be served in the testing facility at approximately (but no less than) 4 hours after intake of TMC207. On Study Day 30, subjects will be discharged from the testing facility after the 24-hour blood collection and administration of rifampin or rifabutin. On Study Days 31 to 41 subjects will return to the testing facility fasted, have their blood drawn, and receive their administration of rifampin or rifabutin. Subjects should be advised that a) if they have eaten within the two hours prior to the scheduled administration, they will have to wait until they have been fasted for two complete hours prior to the scheduled administration; and b) they should not eat for at least one hour after their dose of rifampin or rifabutin.

6.1.6 Accountability/Final Disposition for the Investigational Product(s)

The site pharmacist/registered dispenser will be responsible for dispensing the IMP. Records will be made of the receipt and dispensing of the drugs supplied.

Any remaining unused drugs will either be returned to the Sponsor/designee or destroyed appropriately at the clinical site. If no supplies remain, this fact will be indicated in the drug accountability section of the final report.

7 STUDY PROCEDURES/EVALUATIONS/SCHEDULE

7.1 Clinical/Laboratory Evaluations and Study Schedule

Written informed consent will be obtained before any clinical evaluations are performed. See Appendix A (Schedule of Events) for windows of times indicated below.

This study consists of two Groups of 16 subjects each. This is an open-label study. However, subjects will be randomized to one of two Groups to be treated in parallel through two Periods. Subjects in both Groups will receive a single 400mg oral dose of TMC207 on Day 1 of the study followed by a four-week wash-out period. Then, subjects in both groups will receive a second 400mg oral dose of TMC207 on Day 29 of the study. Concurrently, Group 1 and Group 2 subjects will receive single daily doses of rifabutin (300mg) or rifampin (600mg daily) respectively on Days 20 – 41 (22 total doses) of the study.

The subjects will be staying in the inpatient ward of University Hospitals Case Medical Center (UHCMC) Dahms Clinical Research Unit (DCRU). The DCRU, part of the Case Western Reserve University CTSA, is located on the 7th floor of Rainbow Babies and Children's Hospital (part of UHCMC). DCRU nurses staff the clinical research unit 24 hours daily, seven days per week. The DCRU is an open ward that can allow for freedom of movement. Each subject will have a private room with internet access, television, and food services. Retinal specialists will perform ophthalmological exams at the Retinal Diseases Image Analysis Reading Center (REDIARC) at Case Western University Hospitals Eye Institute. The primary function of REDIARC, founded by Dr. Suber S. Huang (lead study ophthalmologist), is to evaluate fundus photographs, fluorescein angiograms, or other retinal imaging modalities for clinical trials. REDIARC is an experienced Reading Center that has analyzed images for over 2,500 patients, totaling more than 8,000 study visits in the past 10 years. In the course of those studies, REDIARC has certified more than 425 clinical sites and their photographers in 23 countries around the world.

Specifically, during Period 1, subjects in both Groups will be admitted on Day -1 and discharged on Day 2, for a total of 3 days and 2 nights. Subjects in both groups will return to the DCRU on Day 28 where they will be admitted for Period 2. Subjects in both Groups will then remain in the DCRU until discharge on Day 30, for a total of 3 days and 2 nights in Period 2. Thereafter, subjects in both groups will return to the ambulatory component of the DCRU for outpatient blood draws and other assessments on days they are not staying at the DCRU, as indicated below

and in the Schedule of Events (Appendix A). Subjects will also return to the unit on Study Day 57 (28 days after their second TMC207 dose) ±5 days for a safety follow-up visit.

In Period 1, subjects will be required to visit the ambulatory clinic facility for outpatient blood draws on Study Days 3 – 15. On Study Day 7, subjects will have a urine drug screen in addition to the blood draw. On Study Day 15, subjects will have an additional blood draw for safety assessments, urinalysis and alcohol/drug screen, ECG, vital signs and a targeted physical exam if needed.

In Period 2, subjects will be required to visit the ambulatory clinic facility for outpatient blood draws on Study Days 19 through 27 and 31 through 43. On Study Days 19, 36, and 43, subjects will have an additional blood draw for safety assessments, urinalysis, ECG, vital signs, and targeted physical exam if needed. On Study Day 28, any abnormal safety labs will be reviewed by study physician and be determined not to meet the exclusion criteria before administration of the second dose of TMC207. .

Prior to discharge on Study Days 2 (Period 1) and 30 (Period 2), subjects in both groups will be briefly assessed (including a targeted physical examination if necessary) to ensure that he or she is fit and well. If necessary, the subject may be kept at the clinical facility for further observation and/or treatment.

For each subject that completes the full study, the total number of blood draws during the study will be no more than 53 for PK analysis (i.e., 46 x 3mL for TMC207 plus 12 x 5mL for rifampin or rifabutin, which comes to a total of 198mL). An additional 18mL of blood will be drawn for safety monitoring at Screening, and on Study Days -1, 2, 15, 19, 21, 23, 28, 30, 36, 43, and 57. The total volume of blood drawn per subject in this study will be no more than approximately 400mL (about 14 ounces or a little more than 1 and a half cups). Blood volumes are indicated in Appendix B.

PK blood samples (3mL) for TMC207 and M2, will be collected at the following times: at pre-dose and at 1, 2, 3, 4, 5, 6, 8, 12, 24, 48, 72, 96, 120, 144, 168, 192, 216, 240, 264, 288, 312, and 336 hours after TMC207 dosing in both Period 1 and 2; PK blood samples (5mL) for rifabutin, desacetyl rifabutin, rifampin, and desacetyl rifampin will be drawn pre-dose and 2 hours post dose in Period 2 on Study Days 27, 28, 29, 30, 35, and 41 (Appendix B).

Samples from timepoints 0, 1, 4, 8, 12, 72, 120, 168, 240, and 336 will also be used to measure cell-associated levels of TMC207 and its M2 metabolite. Cells will be separated from plasma using Leucosept tubes. It is known that TMC207 binds to phospholipids, thus we will not be able

to distinguish between cell-bound and intracellular levels, and cell-associated levels measured may not be representative of active drug. However, evaluation of cell-associated levels in conjunction with plasma levels will give some indication of available drug that can be delivered to the sites of infection. It is recognized that levels in tissue macrophages would be best, but using PBMCs will enable a surrogate marker. Moreover, given that TB is an intracellular pathogen, cell-associated levels are of interest to the field and to treating physicians. Given that TMC207 may allow TB treatment shortening, cell-associated levels may be critically important.

For PK blood draws through 12 hours post-dose inclusive, an actual draw time ± 10 minutes from the scheduled draw time is allowed. Samples collected at 24 hours should be collected within ±1 hour of the scheduled draw time. For PK blood draws from 48 to 336 hours, a window of 4 hours before/after the scheduled sampling time is allowed. Intake of water is allowed until 2 hours before the intake of trial medication and from 2 hours after intake of trial medication.

Blood draws for a whole blood bactericidal assay will also occur simultaneously with the PK blood draws. The time points for the whole blood assay will be 10 minutes before TMC 207 dosing and, 1, 3, 4, 6, 8, and 12 hours post-dose (± 10 minutes) TMC207 administration (Group 1) and post-dose (± 10 minutes) TMC207 with rifampicin or rifabutin (Group 2). The whole blood BACTEC method (Becton Dickinson) may be useful for the early clinical evaluation of new anti-TB drugs and in the management of individual patients. This assay can be described as an ex vivo model for measuring the whole blood bactericidal activity (WBA) of drugs by whole blood culture.

The following tests will be included as described in the appropriate sections below and as indicated in the Schedule of Events in Appendix A:

Hematology and Coagulation

- Hemoglobin
- Hematocrit
- APTT
- PT
- Red blood cell count
- White blood cell count with differential
- INR
- Platelet count

Serum Chemistry

- Albumin
- Creatinine
- Direct bilirubin
- Total bilirubin
- Lipase (pancreatic)
- Alkaline phosphatase
- Creatine Phosphokinase (CPK)
- Sodium
- Potassium
- Calcium
- Chloride

- Urea
- Uric acid
- Cholesterol, total
- Triglycerides
- Total protein
- AST
- ALT
- Gamma-glutamyl transferase
- Lactate dehydrogenase
- Lipase
- Phosphate
- Fasting glucose
- Creatine phosphokinase MB
- Troponin

Urinalysis

- pH
- Specific gravity
- Protein
- Glucose
- Microalbumin
- Ketones
- Bilirubin
- Nitrite
- Urobilinogen
- Blood
- Leukocyte esterase
- Microscopy

Additional Tests

- HIV-1, -2 ELISA
- Hepatitis A antibody (IgM)
- HBsAg
- HCV antibody
- HCG (if applicable)
- Alcohol (blood level)
- Urine drug screen
  - Cannabinoids
  - Cocaine
  - Amphetamines
  - Opiates
  - Benzodiazepines
  - Barbiturates

7.2 Screening

The Screening visit will occur within 21 days prior to Day -1 for all subjects (both Groups). During the visit, family and personal medical histories (illnesses, surgeries, medications, etc.) and demographic data, including name, sex, age, race, body weight, height, and tobacco/alcohol use will be recorded. Each subject will undergo a physical examination, including a skin examination (partially disrobed), eye examination with fundoscopy, slit lamp exam and retinal photoss, vital signs, and 12-lead ECG. Women will also undergo a pregnancy test. Subjects will also be assessed for inclusion/exclusion criteria. Upon enrollment, each subject will be assigned a unique subject identification number.

The following laboratory tests will be completed at the screening visit:

Hematology and Coagulation

- Hemoglobin
- Hematocrit
- APTT
- PT
- Red blood cell count
- White blood cell count
- INR
- Platelet count

Serum Chemistry

- Creatinine
- Total bilirubin
- Urea
- Uric acid
- Lipase (pancreatic)
- Alkaline phosphatase
- Creatine Phosphokinase (CPK)
- AST
- ALT
- Fasting glucose
- Troponin

Urinalysis

- pH
- Specific gravity
- Protein
- Glucose
- Microalbumin
- Ketones
- Bilirubin
- Nitrite
- Urobilinogen
- Blood
- Leukocyte esterase
- Microscopy

Additional Tests

- HIV-1, -2 ELISA
- Hepatitis A antibody IgM
- HBsAg
- HCV antibody
- HCG (if applicable)
- Alcohol (blood level)
- Urine drug screen
  - Cannabinoids
  - Cocaine
  - Amphetamines
  - Opiates
  - Benzodiazepines
  - Barbiturates

7.3 Enrollment/Baseline

Enrollment/Baseline will occur on Study Day -1 for both Groups. Procedures are indicated in the Schedule of Events in Appendix A, and are as follows. Before study product administration, inclusion/exclusion criteria will be reviewed and the complete screening medical history will be reviewed and updated. Subjects will be questioned about any interim illnesses, any inpatient or outpatient visits, any new medications, and any new symptoms.

Subjects will be admitted to the DCRU. They will undergo a physical examination with skin evaluation, serum pregnancy test (if applicable), serum chemistry, hematology and coagulation, urinalysis, drug/alcohol screen (as listed in Section 7.1), ECG, and vital signs, and women will undergo a pregnancy test. All evaluations will be available and reviewed before administration of study product.

7.4 Study Visits

Subjects will be admitted to the DCRU the day before Day 1 (described below). They will be discharged on Day 2 and undergo multiple outpatient follow-ups. They will be readmitted to the DCRU on Day 28 and discharged on Day 30. Multiple outpatient follow-up visits follow Day 30, as outlined below and in the Appendices.

7.4.1 Clinical and laboratory evaluations for Period 1 (Groups 1 and 2)

Period 1 clinical and laboratory evaluations will be identical for all subjects in Groups 1 and 2. The following evaluations will occur on Study Days 1 – 14 as indicated below and in the Schedule of Events in Appendix A. Adverse events and a log recording intake of concomitant medication will be monitored throughout the trial from the signing of Informed Consent onward until the last trial-related activity.

Day 1:

- ECG within 30 minutes (±15 minutes) before start of breakfast and 4 hours (±1 hour) post-dose
- Vital signs including blood pressure, pulse, respirations, and oral temperature
- Blood pressure and pulse; supine after 5 minutes, standing after 1 minute
- Supervised intake of a single dose of TMC207 (400mg) within 30 minutes after breakfast
- Nine blood draws (3mL each) to measure TMC207 and M2 levels at 10 minutes before TMC207 dosing and 1, 2, 3, 4, 5, 6, 8, and 12 hours post-dose (±10 minutes)
- Seven blood draws (1mL each) for use in the whole blood assay (WBA) at 10 minutes before TMC207 dosing and 1, 3, 4, 6, 8, and 12 hours post-dose (±10 minutes)

Day 2 (24 hours post-TMC207 dose):

- Vital signs including blood pressure, pulse, respirations, and oral temperature
- Blood pressure and pulse; supine after 5 minutes, standing after 1 minute
- ECG approximately 15 minutes prior to blood draw (±15 minutes, must be prior to blood draw)
- Blood draw (3mL) to measure TMC207 and M2 levels and 18mL for hematology and coagulation and serum chemistry tests as listed in Section 7.1
- Urinalysis
- Targeted physical examination if necessary
- Eye exam with fundoscopy and slit lamp exam (to be repeated on Day 15 as deemed necessary by the PI based on results)

Days 3 – 14 (outpatient):

- Blood draws (3mL) to measure TMC207 and M2 levels daily, (i.e. at 48, 72, 96, 120, 144, 168, 192, 216, 240, 264, 288, and 312 hours post-TMC207 dose [±4 hours])
- Blood alcohol level and urine drug screen on Day 7

Day 15 (outpatient):

- Repeat eye exam with fundoscopy and slit lamp exam if necessary
- Vital signs including blood pressure, pulse, respirations, and oral temperature
- Blood pressure and pulse; supine after 5 minutes, standing after 1 minute
- ECG approximately 15 minutes prior to blood draw (±15 minutes, must be prior to blood draw)
- Blood draw (3mL) to measure TMC207 and M2 levels and 18mL for hematology, coagulation,serum chemistry, and blood alcohol tests as listed in Section 7.1
- Urinalysis, including urine drug screen
- Physical examination, including partially disrobed skin examination

7.4.2 Clinical and laboratory evaluations for Period 2 (Groups 1 and 2)

Period 2 clinical and laboratory evaluations will be identical for all subjects in Groups 1 and 2, with the exception of administered drug and, consequently, serum drug level assessments. Subjects in Group 1 will be receiving rifabutin (300mg), whereas subjects in Group 2 will be receiving rifampin (600mg) as indicated below. Thus, serum drug level assessments will measure TMC207 and either rifabutin or rifampin levels as appropriate and indicated below.

The following evaluations will occur on Study Days 19 – 43, as indicated below and in the Schedule of Events in Appendix A. Tests and evaluations are listed in chronological order for each day unless otherwise indicated. Adverse events and intake of concomitant medication will be monitored throughout the trial from the signing of Informed Consent onward until the last trial-related activity.

Day 19 (outpatient):

- Inclusion/exclusion criteria verification

- Serum pregnancy test (for all women)
- Physical examination including skin examination (partially disrobed) and eye examination with fundoscopy slit lamp exam and retinal photos
- Blood draw (18mL) for hematology, coagulation, serum chemistry, and blood alcohol tests as listed in Section 7.1
- Urinalysis, including urine drug screen

Day 20 (outpatient):

- Vital signs including blood pressure, pulse, respirations, and oral temperature
- Blood pressure and pulse; supine after 5 minutes, standing after 1 minute
- ECG
- Supervised intake of rifabutin (300mg, Group 1) or rifampin (600mg, Group 2)

Day 21 (outpatient):

- Supervised intake of rifabutin (300mg, Group 1) or rifampin (600mg, Group 2)
- Urinalysis
- Blood draw (18mL) for hematology and coagulation and serum chemistry tests as listed in Section 7.1

Days 22, 24, 25, 26 (outpatient):

- Supervised intake of rifabutin (300mg, Group 1) or rifampin (600mg, Group 2)

Day 23 (outpatient):

- Supervised intake of rifabutin (300mg, Group 1) or rifampin (600mg, Group 2)
- Urinalysis
- Blood draw (18mL) for hematology and coagulation and serum chemistry tests as listed in Section 7.1.

Day 27 (outpatient):

- Blood draws to measure TMC207, M2, and rifabutin or rifampin levels (8mL) prior to dosing, and rifabutin or rifampin levels (5ml) two hours post-dose.
- Supervised intake of rifabutin (300mg, Group 1) or rifampin (600mg)

Day 28 (inpatient):

- Vital signs including blood pressure, pulse, respirations, and oral temperature
- Blood pressure and pulse; supine after 5 minutes, standing after 1 minute
- ECG approximately 15 minutes prior to blood draw (±15 minutes, must be prior to blood draw)
- Physical examination including skin examination (partially disrobed)
- Blood draws to measure TMC207, M2, and rifabutin or rifampin levels (8mL) prior to dosing, and rifabutin or rifampin levels (5ml) 2 hours post-dose.
- 18mL for hematology, coagulation,serum chemistry, and blood alcohol tests as listed in Section 7.1 prior to dosing
- Urinalysis prior to dosing, including urine drug screen
- Supervised intake of rifabutin (300mg, Group 1) or rifampin (600mg, Group 2)
- Any abnormal safety labs will be reviewed by study physician before administration of the second dose of TMC207 the next day.

Day 29 (inpatient):

- Vital signs including blood pressure, pulse, respirations, and oral temperature
- Blood pressure and pulse; supine after 5 minutes, standing after 1 minute
- ECG at least 30 minutes (±15 minutes) before start of breakfast and 4 hours (±1 hour) post-dosing
- Blood draws (8mL each) to measure TMC207, M2, and rifabutin or rifampin levels immediately (within 10 minutes) prior to dosing and 2 hours post-dose

- Supervised intake of TMC207 400mg plus rifabutin (300mg, Group 1) or rifampin (600mg, Group 2), with TMC207 intake within 5 minutes after rifabutin or rifampin intake. This should occur within 30 minutes after breakfast.

- Seven additional blood draws (3mL each) to measure TMC207 and M2 levels 1, 3, 4, 5, 6, 8, and 12 hours post-dose (±10 minutes)

- Seven blood draws (1mL each) for use in the whole blood assay (WBA) at 10 minutes before TMC207 dosing and 1, 3, 4, 6, 8, and 12 hours post-dose (±10 minutes)

Day 30 (inpatient):

- Vital signs including blood pressure, pulse, respirations, and oral temperature

- Blood pressure and pulse; supine after 5 minutes, standing after 1 minute

- ECG approximately 15 minutes prior to dosing (±15 minutes, must be prior to dosing)

- Blood draws to measure TMC207, M2, and rifabutin or rifampin levels (8mL) prior to dosing, and rifabutin or rifampin levels (5ml) 2 hours post-dose

- Hematology and coagulation and serum chemistry tests (18mL) as listed in Section 7.1 immediately prior to dosing

- Urinalysis prior to dosing

- Supervised intake of rifabutin (300mg, Group 1) or rifampin (600mg, Group 2)

- Targeted physical examination if necessary and eye examination with fundoscopy and slit lamp exam prior to discharge

Days 31 – 34 (outpatient):

- Blood draws (3mL each) immediately prior to dosing to measure TMC207 and M2 levels (i.e., at 48, 72, 96, and 120 ±3 hours post-initial dosage on Day 30)

- Supervised intake of rifabutin (300mg, Group 1) or rifampin (600mg, Group 2)

Day 35 (outpatient):

- Blood draws to measure TMC207, M2, and rifabutin or rifampin levels (8mL) prior to dosing, and rifabutin or rifampin levels (5ml) 2 hours post-dose
- Supervised intake of rifabutin (300mg, Group 1) or rifampin (600mg, Group 2)

Day 36 (outpatient):

- Vital signs including blood pressure, pulse, respirations, and oral temperature
- Blood pressure and pulse; supine after 5 minutes, standing after 1 minute
- ECG approximately 15 minutes prior to dosing (±15 minutes, must be prior to dosing)
- Blood draw (3mL) immediately prior to dosing to measure TMC207 and M2 levels
- Hematology and coagulation and serum chemistry tests (18mL) as listed in Section 7.1 immediately prior to dosing
- Urinalysis prior to dosing
- Supervised intake of rifabutin (300mg, Group 1) or rifampin (600mg, Group 2)

Days 37-40 (outpatient):

- Blood draws (3mL each) immediately prior to dosing to measure TMC207 and M2 levels (i.e., at 192, 216, 240, and 264 ±3 hours post-initial dosage on Day 30)
- Supervised intake of rifabutin (300mg, Group 1) or rifampin (600mg, Group 2)

Day 41 (outpatient):

- Blood draws to measure TMC207, M2, and rifabutin or rifampin levels (8mL) prior to dosing, and rifabutin or rifampin levels (5ml) 2 hours post-dose
- Supervised intake of rifabutin (300mg, Group 1) or rifampin (600mg, Group 2)

Day 42 (outpatient):

- Blood draw (3mL) to measure TMC207 and M2 levels

Day 43 (outpatient):

- Vital signs including blood pressure, pulse, respirations, and oral temperature
- Blood pressure and pulse; supine after 5 minutes, standing after 1 minute
- ECG
- Blood draw (3mL) to measure TMC207 and M2 levels
- Hematology and coagulation and serum chemistry tests (18mL) as listed in Section 7.1
- Urinalysis

7.5 Follow-up Visit

Subjects in both Groups will return on study Day 57 (28 days after last TMC207 intake in Period 2) ±14 business days for a Follow-up visit. As a reminder, adverse events and intake of concomitant medication will be monitored throughout the trial from the signing of Informed Consent onward until the last trial-related activity. During the Follow-up visit, subjects will receive:

- Review of medical history
- Physical examination including skin examination (partially disrobed)
- Eye examination with fundoscopy slit lamp exam and retinal photos
- Serum pregnancy test (for all women)
- Vital signs including blood pressure, pulse, respirations, and oral temperature
- Blood pressure and pulse; supine after 5 minutes, standing after 1 minute
- ECG
- Urinalysis
- Blood draw (18mL) for hematology and coagulation and serum chemistries as indicated in Section 7.1

7.6 Unscheduled Visit(s)

If a subject develops symptoms or incurs illness and needs to be evaluated between scheduled visits, the subject will be asked to come to the clinical research clinic area for assessment. A medical history will be obtained and a targeted physical examination performed by study physicians. Safety laboratories will be repeated if indicated. Findings will be documented on a CRF for unscheduled visits. All AEs will be monitored until they are resolved or determined by the site PI to be medically stable.

7.7 Early Termination

In the case of early termination, the following evaluations will be taken at the time of dropout if the subject is willing:

- Physical examination including skin examination (partially disrobed) and eye examination with fundoscopy and slit lamp exam
- Vital signs including blood pressure, pulse, respirations, and oral temperature
- Blood pressure and pulse; supine after 5 minutes, standing after 1 minute
- Urinalysis
- Blood draw (8mL) to measure TMC207, M2, and rifabutin or rifampin levels plus an additional 18mL for hematology and coagulation and serum chemistries as indicated in Section 7.1

If possible, the following evaluations will be taken 28 days after dropout:

- Physical examination including skin examination (partially disrobed) and eye examination with fundoscopy and slit lamp exam
- Serum pregnancy test (for all women)
- Vital signs including blood pressure, pulse, respirations, and oral temperature
- Blood pressure and pulse; supine after 5 minutes, standing after 1 minute
- ECG
- Urinalysis

- Blood draw (18mL) for hematology and coagulation and serum chemistries as indicated in Section 7.1

7.8 Final Study Visit

See Follow-up Visit, Section 7.5. This is the final study visit for all participating subjects in both Groups 1 and 2 in Period 2.

7.9 Rescue Therapy

A fully stocked emergency crash cart is available in the DCRU, where subjects will be admitted and where the study products will be administered. All DCRU staff is basic life support (BLS)-certified and trained in resuscitation procedures and medications. The University Hospital Case Medical Center resuscitation (Code Blue) team responds immediately to emergency calls for assistance by the DCRU. Subjects will be treated based on symptom presentation, with the exception of medications which affect p450 metabolism. A list of acceptable medications is available in the MOP. Subjects will not be permitted to self-administer medication during the study.

7.10 Specimen Preparation, Handling, and Shipping

7.10.1 Instructions for Specimen Preparation, Handling, and Storage

Preparation:
Blood samples will be collected at the times specified in Section 7.4 in Vacutainer® tubes containing sodium heparin, kept at room temperature. Within one hour after collection, blood samples for PK analysis will be centrifuged at 1500g for seven minutes at room temperature, the plasma separated, and stored in a -20°C freezer. The overall duration of sample processing, from collection to freezing, will be less than two hours after collection. The following processing, storage, and shipping procedures will be followed:

Plasma Sample Storage:

<u>TMC207 and M2</u>

1. This bioanalytical assay requires a total blood volume of 3mL, which will provide approximately 1.4mL of plasma after processing. Using standard laboratory

techniques, an approximate 0.7mL aliquot will be transferred into an appropriately labeled tube. A second aliquot of approximately 0.7mL will be placed in a second labeled tube. The Protocol Pharmacologist will provide specialty tube labels which withstand low temperatures and freeze-thaw cycles will be. Labels should be secured to each storage tube so they do not come off during storage at -20°C or shipping on dry ice. Labels will contain the following information: Protocol number; subject number (e.g., 001, 002, 003, etc.); treatment/study Day; time-point of sample collection (e.g., 8 hours post-dose); analyte; and matrix (i.e., plasma).

2. Samples will be stored frozen and upright at -20°C or lower until shipped.

Rifampin and desacetyl rifampin or rifabutin and desacetyl rifabutin

1. This bioanalytical assay requires a total blood volume of 5mL, which will provide approximately 2.0mL of plasma after processing. Using standard laboratory technique, an approximate 1.0mL aliquot of plasma will be transferred into an appropriately labeled tube. A second aliquot of approximately 1.0mL will be placed in a second labeled tube. The Protocol Pharmacologist will provide tube labels. Labels should be secured to each storage tube so they do not come off during storage at -70°C or shipping on dry ice.

2. Samples will be stored frozen and upright at -70°C or lower until shipped.

7.10.2 Specimen Shipment

After all subjects have completed all study activities, plasma samples for PK determinations will be shipped to the Protocol Pharmacologist in two separate shipments packed in dry ice sufficient to last for three days. The first set of aliquots from all subjects will be sent to the Protocol Pharmacologist. Once the lab confirms the first shipment has arrived in good condition, the second set of aliquots will be sent. Shipments will be made by overnight courier to the following address:

Byron Waldorf / Wes Gray
Health Education Building, Room 210
The University of Toledo
3100 Transverse Drive
Toledo, OH 43614

8 DMID SAFETY REPORTING AND SAFETY MONITORING

Regulatory requirements including the Food and Drug Administration (FDA) regulations, International Conference on Harmonisation (ICH) Guidelines for Good Clinical Practice (GCP), and European Union (EU) Clinical Trials Directive set forth safety monitoring and reporting responsibilities of sponsors and investigators to ensure the safety and protection of human subjects participating in clinical trials.

8.1 Responsibilities

Investigators participating in this clinical trial are responsible for and will:

- Evaluate subject safety including assessment of adverse events (AEs) for seriousness, severity, and causality;
- Notify the sponsor (DMID and TB Alliance) of serious adverse events (SAEs) immediately;
- Provide detailed written reports, including necessary documentation requested by the sponsor or institutional review board (IRB)/independent ethics committee (IEC), promptly following immediate initial reports; and
- Inform the IRB/IEC of AEs as required by applicable regulatory requirements.

8.2 Definitions

Adverse Event (AE)

ICH E6 defines an AE as any untoward medical occurrence in a patient or clinical investigation subject administered a pharmaceutical product regardless of its causal relationship to the study treatment. An AE can therefore be any unfavorable and unintended sign (including an abnormal laboratory finding), symptom, or disease temporally associated with the use of medicinal (investigational) product. The occurrence of an AE may come to the attention of study personnel during study visits and interviews of a study recipient presenting for medical care, or upon review by a study monitor.

All AEs including local and systemic reactions not meeting the criteria for “serious adverse events” should be captured on the appropriate CRF. Information to be collected includes event description, time of onset, clinician’s assessment of severity, relationship to study product (assessed only by those with the training and authority to make a diagnosis, which would include

MD, PA, Nurse Practitioner, DO, or DDS), and time of resolution/stabilization of the event. All AEs occurring while on study must be documented appropriately regardless of relationship. All AEs will be followed to adequate resolution.

Any medical condition that is present at the time that the patient is screened should be considered as baseline and not reported as an AE. However, if it deteriorates at any time during the study, it should be recorded as an AE.

All AEs must be graded for severity and relationship to study product. FDA defines an AE as any untoward medical occurrence associated with the use of a drug in humans, whether or not considered drug related.

Severity of Event:

All AEs will be assessed by the clinician using a protocol defined grading system. For events not included in the protocol defined grading system, than the following guidelines will be used to quantify intensity.

1. Mild: events require minimal or no treatment and do not interfere with the patient’s daily activities.
2. Moderate: events result in a low level of inconvenience or concern with the therapeutic measures. Moderate events may cause some interference with functioning.
3. Severe: events interrupt a patient’s usual daily activity and may require systemic drug therapy or other treatment. Severe events are usually incapacitating.
4. Life threatening: any adverse drug experience that places the patient or subject, in the view of the investigator, at immediate risk of death from the reaction as it occurred, ie, it does not include a reaction that had it occurred in a more severe form, might have caused death.

Changes in the severity of an AE should be documented to allow an assessment of the duration of the event at each level of intensity to be performed. Adverse events characterized as intermittent require documentation of onset and duration of each episode.

Serious Adverse Event (SAE)

An adverse event or suspected adverse reaction is considered “serious” if, in the view of either the investigator or sponsor, it results in any of the following outcomes:

- Death,
- a life-threatening adverse event*,

- inpatient hospitalization or prolongation of existing hospitalization,
- a persistent or significant incapacity or substantial disruption of the ability to conduct normal life functions,
- a congenital anomaly/birth defect,
- Grade 3 or grade 4 laboratory toxicities as described in Section 8.4, or
- Important medical events that may not result in death, be life-threatening, or require hospitalizations may be considered serious when, based upon appropriate medical judgment they may jeopardize the patient or subject and may require medical or surgical intervention to prevent one of the outcomes listed in this definition. Examples of such medical events include allergic bronchospasm requiring intensive treatment in an emergency room or at home, blood dyscrasias or convulsions that do not result in inpatient hospitalization, or the development of drug dependency or drug abuse.

* Life-threatening adverse event. An adverse event is considered "life-threatening" if, in the view of either the investigator or sponsor, its occurrence places the patient or subject at immediate risk of death. It does not include an adverse event, had it occurred in a more severe form, might have caused death.

All SAEs will be:

- recorded on the appropriate SAE CRF
- followed through resolution by a study clinician
- reviewed and evaluated by a study clinician

Unexpected

An unexpected AE is an adverse reaction, the nature or severity of which is not consistent with the applicable product information (e.g., investigator's brochure (IB) for an unapproved investigational medicinal product).

Expedited Safety Report

An expedited safety report is documentation in appropriate form and format summarizing an SAE that meets expedited safety reporting criteria, submitted within the required reporting time frame of applicable regulatory authorities and/or IRBs/IECs of participating countries.

8.3 Safety Reporting Requirements

8.3.1 Reporting Interval

Document all AEs and SAEs from the signing of the Informed Consent form (Day -1) through Study Day 57.

All SAEs will be followed until resolution, even if this extends beyond the study-reporting period. Resolution of an AE is defined as the return to normal range or stabilization of the condition with the expectation that it will remain chronic.

At any time after completion of the study, if the investigator becomes aware of an SAE that is suspected to be related to study product, the investigator will report the event as indicated in Section 8.3.2.

8.3.2 Notification of the Sponsor of Serious Adverse Events

Any AE that meets a protocol-defined serious criterion must be submitted within 24 hours of site awareness on an SAE form to the DMID pharmacovigilance contractor, at the following address:

DMID Pharmacovigilance Group
Clinical Research Operations and Management Support (CROMS)
6500 Rock Spring Dr., Suite 650
Bethesda, MD 20814, USA
SAE Hot Line: 1-800-537-9979 (US) or 1-301-897-1709 (outside US)
SAE FAX Phone Number: 1-800-275-7619 (US) or 1-301-897-1710 (outside US)
SAE Email Address: PVG@dmidcroms.com

Other supporting documentation of the event may be requested by the DMID pharmacovigilance contractor and should be provided as soon as possible. The DMID pharmacovigilance contractor will notify the DMID medical monitor, clinical protocol manager, and the TB Alliance of the SAE. The DMID medical monitor will review and assess the SAE for regulatory reporting and potential impact on study subject safety and protocol conduct. At any time after completion of

the study, if the investigator becomes aware of an SAE that is suspected to be related to study product, the investigator will report the event to the DMID Pharmacovigilance Group.

8.3.3 Regulatory Reporting for Studies Conducted Under DMID-Sponsored IND

Following notification from the investigator, DMID, the IND sponsor, will report any suspected adverse reaction that is both serious and unexpected. DMID will report an adverse event as a suspected adverse reaction only if there is evidence to suggest a causal relationship between the drug and the adverse event. DMID will notify FDA and all participating investigators (i.e., all investigators to whom the sponsor is providing drug under its INDs or under any investigator's IND) in an IND safety report of potential serious risks from clinical trials or any other source, as soon as possible, but in no case later than 15 calendar days after the sponsor determines that the information qualifies for reporting as specified in 21 CFR Part 312.32. DMID will also notify FDA of any unexpected fatal or life-threatening suspected adverse reaction as soon as possible but in no case later than 7 calendar days after the sponsor's initial receipt of the information. ~~:~~ Relevant follow-up information to an IND safety report will be submitted as soon as the information is available. Upon request from FDA, DMID will submit to FDA any additional data or information that the agency deems necessary, as soon as possible, but in no case later than 15 calendar days after receiving the request.

All serious events designated as "not related" to study product(s), will be reported to the FDA at least annually in a summary format.

8.3.4 Reporting of Pregnancy

Women with known or suspected pregnancy are excluded from the trial. However, if pregnancy is discovered during the follow-up period or up to 12 weeks after administration of study drug, the investigator must report information using the Pregnancy Report Form to CRM. All pregnancies will be followed up to final outcome, using the pregnancy follow-up form. At that time, the status of the mother and infant will be noted, including the date of delivery and the infant's gender and weight.

The outcome, including any premature termination, must be reported to CRM. The pregnancy is not considered an AE; however pregnancy complications, including miscarriage or spontaneous abortion, are considered AEs. Any untoward outcome for the mother or infant is considered an SAE. The report of any pregnancy and the outcomes of pregnancy as outlined above will be reported to the DMID Medical Monitor.

8.4 Investigator's Assessment of Adverse Events

The determination of seriousness, severity, and causality will be made by an on-site investigator who is qualified (licensed) to diagnose AE information, provide a medical evaluation of AEs, and classify AEs based upon medical judgment. This includes but is not limited to physicians, physician assistants, and nurse practitioners.

Laboratory abnormalities will be reported as AEs based on the normal range of values for the laboratory at UHCMC. Only those laboratory abnormailities which are judged to be clinically significant by the investigator will be recorded as AEs. Those laboratory abnormalities which are judged not to be clinically significant by the investigator will be recorded in the source documents with a rationale for considering such laboratory abnormalities as not clinically significant. Grade 3 toxicities will be reported to DMID regardless of clinical significance and any judged to be clinically significant will be considered an SAE. All grade 4 events, regardless of clinical significance, will be considered SAEs. The grading of the laboratory AEs will be based on the toxicity table in Appendix C. For values that are not in the toxicity table, the grading tables that have been prepared by the Investigators and are appended will be used.

8.4.1 Assessment of Seriousness

Event seriousness will be determined according to the protocol definition of an SAE (see Section 8.2).

8.4.2 Assessment of Severity

Event severity will be assigned according to the DMID Adult Toxicity Table (Appendix C) and as outlined below:

**GRADE 1**	**Mild –** Transient or mild discomfort (< 48 hours); no medical intervention/therapy required
**GRADE 2**	**Moderate –** Mild to moderate limitation in activity - some assistance may be needed; no or minimal medical intervention/therapy required
**GRADE 3**	**Severe –** Marked limitation in activity, some assistance usually required; medical intervention/therapy required, hospitalizations possible
**GRADE 4**	**Life-threatening** – Extreme limitation in activity, significant assistance required; significant medical intervention/therapy required, hospitalization, or hospice care probable

Serious or life-threatening AEs: ANY clinical event deemed by the clinician to be serious or life threatening should be considered a grade 4 event. Clinical events considered serious or life threatening include, but are not limited to: seizures, coma, tetany, diabetic ketoacidosis, disseminated intravascular coagulation, diffuse petechiae, paralysis, acute psychosis, and severe depression.

8.4.3 Assessment of Relationship to Study Products

Relationship to Study Products: The clinician's assessment of an AE's relationship to test article (vaccine or study drug) is part of the documentation process, but it is not a factor in determining what is or is not reported in the study. If there is any doubt as to whether a clinical observation is an AE, the event should be reported. All AEs must have their relationship to study product assessed using the terms: related or not related. In a clinical trial, the study product must always be suspect. To help assess, the following guidelines are used.

- Related– There is a reasonable possibility that the study product caused the adverse event. Reasonable possibility means that there is evidence to suggest a causal relationship between the study product and the adverse event.

- Not Related– There is **not** a reasonable possibility that the administration of the study product caused the event.

The investigator must provide an assessment of relationship of AEs to the study product based on:

- Temporal relationship of the event to the administration of study product;
- Whether an alternative etiology has been identified;
- Biological plausibility; and
- Existing therapy, and/or concomitant medications.

There are several symptoms, signs, and laboratory abnormalities that have been reported with TMC207 (see Section 2.3). In preclinical studies, data from repeat dose toxicity studies indicate that the target organs affected by TMC207 include the mononuclear phagocytic system, skeletal and cardiac muscle, testes, stomach, liver, pancreas, and eye (cornea). In clinical studies to date, the most commonly observed side effects with TMC207 have included headache, nasopharyngitis, rash, and postural dizziness. Known side effects with rifampin have included rash, fever and hypersensitivity reactions, leukopenia, anemia, thrombocytopenia, nausea/vomiting, diarrhea, dizziness, headache, and hepatitis. The main concerns associated with

the use of rifabutin are headache, diarrhea, anorexia, vomiting, uveitis, thrombocytopenia, and neutropenia. These symptoms, signs, laboratory, and ECG will be collected from the subjects while under treatment in the clinical research inpatient unit. In addition, serial ophthalmological evaluations including slit lamp exams will be performed (see Section 7). Adverse events after Discharge will be assessed at the Follow-up visit and will be aided by diaries that will be supplied to the subjects.

8.5 Safety Monitoring by the DMID Safety Oversight Mechanism

Independent Safety Monitor

The independent safety monitor (ISM) is a physician with relevant expertise whose primary responsibility is to provide independent safety monitoring in a timely fashion. The ISM will review SAEs and other AEs as needed and provide an independent assessment to DMID.

8.5.1 Safety Monitoring Committee

This clinical study will utilize a Safety Monitoring Committee (SMC), which is an independent group of experts that advises DMID and the study investigators for many Phase I and smaller Phase II studies. The primary responsibility of the SMC is to monitor subject safety. The committee is external to DMID and composed of at least three voting members. Its activities will be delineated in an SMC charter that will define membership, responsibilities, and the scope and frequency of data reviews. The SMC will operate on a conflict-free basis independently of the study team. DMID or the SMC may convene ad hoc meetings of the SMC according to protocol criteria, or if there are concerns that arise during the study. The SMC will have access to unblinded data during its closed session, if applicable. After its assessment, the SMC will recommend continuation, modification, or termination of the clinical study. The SMC will meet prior to commencement of this study to become familiar with the protocol and endorse commencement of the study. Thereafter, the SMC will next meet after studies are completed for both Groups prior to finalization of the final study report. Criteria for halting any dosing in the two Groups will include the occurrence of two grade 3 or a single instance of a grade 4 toxicity (except hyperuricemia, unless associated with clinically significant event; see Section 8.6). If serious and/or unexpected adverse events occur, the SMC will convene ad hoc to review these events.

8.5.2 Interruption or Discontinuation of Study Enrollment and Study Product Administration for all Subjects in the Study

DMID, when it is the study sponsor, may interrupt study dosing and/or study entry at any time if medically indicated. To minimize risk, the medical monitor, ISM or SMC, as applicable, will review cumulative safety data as expeditiously as reasonable. The study enrollment and dosing will be stopped, and an ad hoc review will be performed if any of the following occur:

1. Death of an enrolled subject;

2. Occurrence of a life-threatening allergic/hypersensitivity reaction (anaphylaxis), manifested by bronchospasm with or without urticaria or angioedema requiring hemodynamic support with pressor medications or mechanical ventilation;

3. An overall pattern of symptomatic, clinical, or laboratory events that the medical monitor, ISM, or SMC consider associated with study product and that may appear minor in terms of individual events, but that collectively, may represent a serious potential concern for safety.

Upon completion of this review and receipt of the advice of the ISM or SMC, DMID will determine if study entry or study dosing should be interrupted or if study entry and study dosing may continue according to the protocol.

8.6 Halting Criteria/Rules

Administration of further doses of the study drug for any subject in the Groups being studied will be halted if any of the following occur:

1. Death of an enrolled subject;
2. Occurrence of a life-threatening allergic/hypersensitivity reaction (anaphylaxis) or IgE-mediated hypersensitivity within 24 hours after receiving study drug, manifested by:
    - Bronchospasm with or without urticaria or angioedema requiring hemodynamic support with pressor medications or mechanical ventilation,
    - Skin or mucous membrane manifestations: hives, pruritis, flushing, swollen lips, tongue, or uvula
    - Respiratory compromise evidenced by dyspnea, wheezing, stridor, hypoxia, or tachypnea to ≥ 28 bpm
    - A confirmed decrease in systolic blood pressure to < 90 mmHg supine or > 30% decrease from baseline
    - Tachycardia with an increase in resting heart rate to ≥ 130 bpm or > 30% increase from baseline
    - Clinically significant syncope, excluding vasovagal associated with blood draws
    - Clinically significant confusion
    - Any other symptom or sign that the study physician thinks warrants halting further dosing of study product. Severe gastrointestinal symptoms such as nausea, abdominal cramping, and diarrhea may be manifestations of anaphylaxis
3. An overall pattern of symptomatic, clinical, or laboratory events that the medical monitor or ISM and SMC consider associated with study product, and that may appear minor in terms of individual events, but collectively, may represent a serious potential concern for safety.

4. Further dosing of study drug is halted if two grade 3 clinical or laboratory toxicities occur in any body system in anyone subject, or one grade 3 clinical or laboratory toxicity occurs in any body system in any two subjects, or one grade 4 clinical or laboratory toxicity occurs in any body system in anyone subject, with the exception of hyperuricemia or lymphocytopenia of grade 4 severity which persists for up to 6 days. If any subject has grade 4 lymphocytopenia of 7 days or longer duration, the study halting rule will be met, the study drug will not be administered to any additional subjects, and enrollment will stop until the SMC reviews the event.

5. Although hyperuricemia has been observed in some studies involving TMC207, it has always been associated with pyrazinamide use. Therefore, unless hyperuricemia is associated with a clinically significant event (e.g., gout), it will not be used as a halting criterion.

9 CLINICAL MANAGEMENT OF EVENTS

The outcomes measures for this study are as outlined in Section 3.2. They include the pharmacokinetics effect of repeated doses of 300mg rifabutin or 600mg rifampin on a single dose of TMC207 and its M2 metabolite in healthy subjects will be determined by measuring plasma levels at multiple time points.

The safety and tolerability of TMC207 when given as a single dose in combination with rifabutin or rifampin will be evaluated by serial assessment of the occurrence of solicited and unsolicited adverse events, including symptoms, physical findings, laboratory testing (hematology, chemistries, urinalysis), ophthalmologic exams, and ECG changes.

9.1 Adverse Event Management

Any AE (serious or non-serious) observed by the investigator or reported by the subject will be recorded on the Adverse Event Case Report Form. If possible, the investigator should record diagnosis of the AE. In the case where an overall diagnosis cannot be made, each specific sign and/or symptom should be recorded as individual AEs.

The investigator will review each AE and assess its relationship to drug treatment based on all available information at the time of the completion of the case report form. Each sign or symptom reported will be graded on a 4-point severity scale using the definitions according to the DMID Adult Toxicity Table (Appendix C). Additionally, the date and time of onset, temporal relationship to IMP dosing, duration, action taken (none, therapy, hospitalization, IMP stopped), and outcome (recovered with sequelae, recovered without sequelae, persisting, fatal, or unknown [lost to follow-up]) of each event will be noted.

9.1.1 Temporary Interruption of Study Product in an Individual Subject

Further dosing of the study product in an individual subject will be stopped if one or more halting criteria are met (see Section 8.6).

9.1.2 Pregnancy (if applicable)

If pregnancy occurs in a subject after she has received the study product, the site will continue to follow the subject until the end of the pregnancy to determine the outcome of the pregnancy (see Section 8.3.4).

10 CLINICAL MONITORING/SITE MONITORING PLAN

Monitoring will be conducted to ensure that human subjects' rights and well-being are protected, data are accurate, complete, and verifiable from source documents, and the study complies with the protocol/amendment(s), ICH Good Clinical Practice (GCP) guidelines, and applicable regulatory requirements.

In order to ensure protocol compliance, monitoring visits will occur at scheduled intervals prior to and during the study, as well as at study completion. The visit frequency will be defined in a monitoring plan approved by the DMID.

A separate study-specific monitoring plan will define the monitoring details. A CRM Clinical Research Associate (CRA) will be responsible for the clinical monitoring of the study at University Hospitals Case Medical Center (UHCMC). The CRM CRA will operate independently from UHCMC and comply with CRM standard operating procedures (SOPs). The CRA will ensure that the investigator understands the investigational status of the product, all requirements of the protocol, and his/her regulatory responsibilities as an investigator. The CRA will visit the clinical site at appropriate intervals in accordance with the monitoring plan to ensure compliance with the protocol, verify accuracy and completeness of the data, perform accountability of investigational product, and review Essential Regulatory Documents for compliance with DMID and ICH guidelines, and applicable regulatory requirements.

The investigational site will provide direct access to all trial-related documents and records maintained by the investigator, including but not limited to, trial-related source data and pharmacy records for the volunteers in this study. The frequency of monitoring visits and percentage and selection of charts/source documentation to be monitored will be outlined in the monitoring plan.

The global principal investigator is ultimately responsible for ensuring that the CRA's findings are addressed. All reports on monitoring activities will be submitted to the DMID, the global principal investigator, and the site principal investigator.

11 STATISTICAL CONSIDERATIONS

It is planned that 16 subjects will be enrolled in each treatment group (rifampin or rifabutin). The number of subjects to be included is based upon clinical judgment and feasibility and is selected to provide adequate information on the potential effects of rifabutin and rifampin on the pharmacokinetics of TMC207 according to the following statistical statements.

Pharmacokinetics:
Sample from all subjects will undergo pharmacokinetic analysis. This analysis compares the ratio of the treatment effect of rifabutin versus the treatment effect of rifampin where the treatment effect is defined as the ratio of test/reference AUC_{0-t} or C_{max} where test is Plus Inducer and reference is Minus Inducer. If the true geometric mean ratios (rifabutin effect over rifampin effect) of TMC207 AUC and C_{max} are 1.26 and 1.38, respectively, then there is 80% power to yield a 90% CI for each GMR (that for AUC and that for C_{max}) greater than 1.0. This would indicate a statistically significantly greater TMC207 AUC and C_{max} for rifabutin than for rifampin (alpha=0.05, 1-sided). The half-widths of the 90% CI's for GMR's of TMC207 AUC and C_{max} are approximately ±16% and ±23%, respectively. These computations are based on estimated within-subject SDs on log scale of 0.32 and 0.22 for TMC207 C_{max} and AUC, respectively. These estimates derive from Study R207910BAC1003 estimates of 0.28 and 0.19 [Data on file] and are corrected for the small sample size in that study using the method of Browne (Browne, 1995).

Summary statistics (n, mean, median, standard deviation, standard error of the mean, within subject coefficient of variation estimated via log-scale SD x 100% based on the analysis model root mean squared error, minimum, maximum) will be prepared for demographic and baseline characteristics.

Descriptive statistics including n, mean, median, standard deviation, standard error of the mean, within subject coefficient of variation (RMSE from analysis model x 100%), minimum, and maximum will be computed for TMC207 and M2; rifampin and desacetyl rifampin; and rifapentin or desacetyl rifapentin plasma concentrations by time following last dose, treatment period, and treatment.

Descriptive statistics including n, mean, median, standard deviation, standard error of the mean, within subject coefficient of variation (RMSE from analysis model x 100%), minimum, and maximum will be computed for each TMC207 and M2 PK parameter by treatment period and treatment. Descriptive statistics including n, mean, median, standard deviation, standard error of

the mean, with-in subject coefficient of variation (RMSE from analysis model x 100%), minimum, and maximum will also be computed for pre-dose or C_{min} plasma concentrations of rifampin, desacetyl rifampin, rifabutin and desacetyl rifabutin on Study Days 27, 28, 29, 30, and 42.

Descriptive statistics for $AUC_{(0-t),}$ $AUC_{(0-inf)}$ and C_{max} for TMC207 and M2 will be provided for each treatment period and treatment. In addition, mean and median concentration-versus-time graphs will be provided (with error bars as appropriate).

The following least squares geometric mean ratios and 90% CI's will be computed for AUC and C_{max} data for each of TMC207 and M2:

- with rifabutin over without rifabutin treatments
- with rifampin over without rifampin treatments, and
- (with rifabutin over without rifabutin) over (with rifampin over without rifampin), i.e., ratio of ratios.

These computations will be derived from a mixed analysis of variance model including a fixed effect term for treatment and a random effect term for subject. All AUC and C_{max} data will be log-transformed for analysis, and difference results on log scale back-transformed to yield GMR's and their CI's.

Other PK parameters will be analyzed similarly. Distributional assumptions of normality and homogeneity will be accessed via graphical techniques. If distribution of residuals from analysis model deviate substantially from normality and homogeneity, then appropriate transformations will be explored and employed if appropriate. These include the rank transformation as a last resort.

Complete details on the statistical model or models to be used in the analysis will be detailed in a stand-alone Statistical Analysis Plan (SAP).

11.1 Study Hypotheses

Hypothesis:
Rifabutin affects the pharmacokinetics of TMC207 as measured by effects on C_{max} and $AUC_{(0-t)}$ to a lesser degree than rifampin. This hypothesis will be supported by a 90% CI for geometric mean ratio (rifabutin over rifampin) entirely greater than 1.0.

11.2 Sample Size Considerations

It is planned that 16 subjects will be enrolled in each treatment group (rifampin or rifabutin) in this study. The number of subjects to be included is based upon clinical judgment and feasibility and is selected to provide adequate information on the potential effects of rifabutin and rifampin on the pharmacokinetics of TMC207 according to the statistical statements provided above.

11.3 Randomization

The randomization list will be generated by the Study Biostatistician and transferred to the study pharmacist prior to the start of the study. As necessary, additional recruitment of subjects for Groups 1 and 2 will be considered by the Principal Investigator to replace subjects should they withdraw from the study. The Study Biostatistician will conduct an on-site training session with the pharmacist before the start of the study if needed, and perform a randomization audit at the site with the study pharmacist at the end of each Period.

11.4 Blinding

This is an open label study, thus blinding is not applicable.

11.5 Planned Interim Analyses (if applicable)

Not applicable for this study.

11.6 Safety Review

Safety events triggering a pause in the study and/or a halt of the study are described in Section 8.6. There are no statistical stopping rules or statistical testing to be conducted for the decision to halt the study.

11.7 Final Analysis Plan

All statistical analyses and mock tables, listings, and figures for this study will be documented in a stand-alone Statistical Analysis Plan. Analysis populations will be created by Group (overall). Demographic data and baseline characteristics will be described and presented appropriately for continuous (mean, standard deviation, median, minimum, and maximum) and for categorical (frequency and percentage) data. Deaths, serious adverse events (SAEs), and adverse events (AEs) will be summarized by Group and between Groups.

Additionally, AE data will be summarized by:

- System organ class (SOC) and preferred term (PT)
- Severity
- Relationship to study drug and severity

Missing data is expected to be minimal. Each Group analysis population will be defined and presented in summary tables and any missing data for subjects will be detailed in footnotes on the applicable table or indicated as a “missing” category in the presentation of the analysis results.

There are no identified issues in this study for multiplicity, and no adjustments for multiple comparisons will be made.

12 DATA HANDLING/RECORD KEEPING/SOURCE DOCUMENTS

It is the responsibility of the Global Principal Investigator and Site Principal Investigator to ensure that all team members handle data and related documentation appropriately. All subject information, including source documents and laboratory reports, must be reviewed by the PI and clinical team and submitted to the CRM Data Management Team. CRM Data Management will work with the PI(s), CRAs, and Case to develop a data management plan, including eCRF creation and instructions for the study to establish, maintain, and update the study data effectively. To maintain up-to-date information, all clinical and laboratory data will be entered directly from the source documents to the data management system within 72 hours of trial activity. There will be ongoing processing of data and quality checks. CRAs will be responsible for source verification of the eCRFs. Adverse Events will be graded according to the November 2007 draft DMID Adult Toxicity Tables, assessed for severity and causality by study staff, and reviewed and approved by the PI.

The Data Coordinating Center (DCC) will utilize Oracle Clinical Remote Data Capture (RDC) version 4.6 to manage the data. The DCC will follow a data management plan, which includes a data quality and validation plan. A data quality plan outlines specific quality control checks to ensure the integrity of the data. Discrepancy management processes are included in the data management plan to facilitate communication to the site for clarification and resolution of data.

Data security is of paramount concern, and several checks have been built in to assure proper data handling including confidentiality of data. A comprehensive IT Systems security plan with a disaster recovery plan is in place at CRM to safeguard data and systems related to this clinical trial.

12.1 Data Capture Methods

All clinical data (including AEs and concomitant medications) will be entered into the Oracle Clinical Data Management system via Remote Data Capture. This system is a 21 CFR Part 11-compliant Internet Data Entry System (IDES) provided by the CRM Data Coordinating Center. Oracle Clinical will be used to receive, enter, verify, label, process, edit, update, correct, freeze, lock, store, secure, track, and retrieve all clinical and laboratory data. Only those who are fully trained on the IDES and delegated the responsibility from the PI with documentation on the signature delegation log will collect and enter data. The data system includes password

protection and internal quality checks, such as heuristic checks, to identify data that appear inconsistent, incomplete, or inaccurate.

12.2 Types of Data

Data for this study will include demographics, AEs, clinical laboratory data, and pharmacokinetic measurements.

12.3 Timing and Reports

Clinical data will be entered directly from the source documents to the data management system within 72 hours of trial activity. CRAs will perform source verification and query generation. CRAs will work with Data Manager and site personnel to freeze data prior to analysis. Adverse events, medical history, and medications will be coded using MedDRA, and WHO drug dictionaries and data analysis will be performed at time points determined by the SMC and outlined in the Data Management Plan.

Recruitment, follow-up, and logistical reports will be scheduled and distributed to necessary personnel via e-mail, website, or other secure method. All reports will be online to query real time data to support ongoing monitoring of data quality and subject safety. DMID personnel with database passwords will be able to access real-time reports. DMID requirements on expedited reporting will be followed.

12.4 Study Records Retention

Case will maintain appropriate medical and research records for this trial, in compliance with ICH E6, Section 4.9, regulatory and institutional requirements for the protection of confidentiality of subjects. Case will permit authorized representatives of the sponsor (DMID), CRM, and regulatory agencies to examine (and when required by applicable law, copy) clinical records for the purposes of clinical site monitoring, quality assurance reviews, audits, and evaluation of the study safety and progress.

Study documents will be retained for a minimum of 2 years after the last marketing application approval or 2 years from the formal discontinuation of clinical development of an investigational product. These documents will be retained for a longer period, however, if required by local regulations. No record will be destroyed without the written consent of the Sponsor.

12.5 Source Documents

The Case staff may use study-specific paper data collection forms that mirror each EDC form to enter data from the source (the subject's chart or other medical records) onto the form provided. Clinical and research laboratory reports will be printed from the laboratory system and utilized as sources. In addition, hard copy data collection forms derived from the CRFs will be utilized at Case for source documents. To maintain up-to-date information, all clinical and laboratory data will be entered directly from the source documents to the Oracle Clinical data management system within 72 hours of trial activity. The Data Manager at Case will be responsible for ensuring all data is entered per study requirements.

12.6 Protocol Deviations

A protocol deviation is any noncompliance with the clinical trial protocol or Good Clinical Practice (GCP) requirements. The noncompliance may be either on the part of the subject, the investigator, or the study site staff. As a result of deviations, corrective actions are to be developed by the site and implemented promptly.

It is the responsibility of the site PI to use continuous vigilance to identify and report deviations to the Global PI via the CRM Coordinating Center within 3 working days of identification of the protocol deviation or of the scheduled protocol-required activity. The CRM coordinating center will report all protocol deviations to the DMID within 5 days of identification by the site. Appropriate reporting forms to CRM and DMID must be maintained in the regulatory file, as well as in the subject's source document. The site PI is responsible for ensuring all study staff understand the local IRB reporting guidelines and adhere to all related requirements and documentation.

These practices are consistent with ICH E6 (refer also to Section 13, Quality Control and Quality Assurance).

13 QUALITY CONTROL AND QUALITY ASSURANCE

Processes outlined in the Clinical Quality Management Plan will be applied to define procedures for quality assurance and quality control at the investigative site for this protocol.

The Quality Assurance Specialist will develop, implement, and oversee all functions of the DMID-accepted version on file of the Quality Management Plan (QMP). The site PI is responsible for the Total Quality Management Committee and its functions and oversight of the CQMP at Case. The Clinical Research Site Quality Management Coordinator (QMC) will be responsible for quality assurance and quality control activities at the site.

Clinical research processes and data collected from this protocol will be evaluated for compliance with the protocol and accuracy with source documents by means of source verification and product accountability performed by the QMC. The Quality Assurance Specialist from CRM will perform site audits of data, Essential Regulatory Documents, and Test Article accountability. The minimal percentage of records selected for review by the QA Specialist will be ten percent (100% of at least 10% of the enrolled subject's source and CRF data). To proactively assure compliance with protocol procedures and assessment of QM activities, 100% of the first three subjects will be audited. The QA Specialist will also review Site Monitoring Reports and Quality Management Summary Reports to ensure that research processes and data collected from the protocol are compliant with protections for human subjects, applicable federal regulations and Good Clinical Practice.

The following are the Key Quality Indicators that will be audited in each volunteer record selected for QA review, including but not limited to, procedures,(Informed Consent, eligibility, specimen collection, study product administration) and documents (Informed Consent Forms, Case Report Forms, study product accountability, and 100% of AE/SAEs).

The site's clinical staff and QMC will perform Quality Control of records by reviewing 100% of all study visit documentation. Data review by the clinical staff will occur within approximately 24 hours of receipt of volunteer data.

Quality Management records will remain on site in a secure location. The clinical study site will permit access to such records to CRM and DMID.

14 ETHICS/PROTECTION OF HUMAN SUBJECTS

14.1 Ethical Standard

Case Western Reserve University is committed to the integrity and quality of the clinical research it coordinates and implements. Case will assure that the legal and ethical obligations associated with the conduct of clinical research involving human subjects are met. The key regulations and ethical standards governing the protection of human subjects that will be implemented are the following:

- Title 21 CFR Part 50, Subpart A: General Provisions and Subpart B Informed Consent FDA
- Title 21 CFR Part 56, Subparts A – E, Institutional Review Boards
- Title 45 CFR Part 46, Protection of Human Subjects, "Common Rule"
- FDA Information Sheets: Guidance for IRBs and Clinical Investigators, 1998 Update
- International Conference on Harmonization, GCP, (ICH E6): Consolidated Guideline, May 9, 1997
- The Belmont Report

As the Department of Health and Human Services continues to strengthen procedures for human subjects' protection via new regulations, Case will review these evolving standards in relation to these proposed studies and will inform the investigators on those that may apply.

The Case/University Hospitals Case Medical Center IRB/IEC will review this protocol and all protocol-related documents and procedures as required by OHRP and local requirements before subject enrollment. The Case/University Hospitals Case Medical Center IRB currently holds and will maintain a US FWA issued by OHRP for the entirety of this study. The FWA for Case/University Hospitals Case Medical Center is FW A00002989.

This assurance commits a research facility to conduct all human subject research in accordance with the ethical principles in the Belmont Report and any other ethical standards recognized by OHRP. Finally, per OHRP regulations, the research facility will ensure that the mandatory renewal of this assurance occurs at the times specified in the regulations.

14.2 Institutional Review Board

The Case/University Hospitals Case Medical Center IRB will review this protocol and all protocol-related documents and procedures as required by OHRP and local requirements before

subject enrollment. The Case IRB currently holds and will maintain a US FWA issued by OHRP for the entirety of this study.

15 INFORMED CONSENT PROCESS

All subjects must be adequately informed in a language that they can understand and read of the aims, methods, anticipated benefits, potential hazards, and the discomfort the study may entail, as well as their right to abstain from participating in the study and to withdraw their consent at any time without affecting or jeopardizing their medical care.

The Principal Investigator is responsible for administering and obtaining freely given consent, in writing, before entering the subject into the study and performing any study-related procedures. Subjects will be required to read, sign, and date an informed consent form summarizing the discussion at Screening. Written informed consent should be documented by the subject's personally dated signature and the personally dated signature of the Principal Investigator or designee who conducted the informed consent discussion. A copy shall be given to the person signing the form.

In obtaining and documenting informed consent, the Principal Investigator should comply with the applicable regulatory requirements and should adhere to ICH GCP (E6). If important new information is incorporated in the consent form and approved by the Institutional Review Board (IRB), informed consent must be repeated for all subjects still actively participating in the study and a new informed consent document must be signed.

The monitor will inspect the original consent form(s) for all subjects.

15.1 Subject Confidentiality

All site staff, the Sponsor, and any sponsor representatives will preserve the confidentiality of all subjects taking part in the study, in accordance with GCP, local regulations, and to the extent applicable, the Health Insurance Portability and Accountability Act of 1996 (HIPAA). Subject to the requirement for source data verification by the study personnel by reference to the subject's notes, confidentiality of all subject identities will be maintained. Only subject initials, date of birth, and study number will be used on the CRF and in all study correspondence, as permitted. No material bearing a subject's name or personal identifiers will be kept on file by the Sponsor.

15.2 Principal Investigator Responsibility When Subject Withdraws or is Discontinued

If a subject withdraws after receiving any of the study products, the subject will be asked to return for safety visits according to the Schedule of Events (Appendix A) for safety evaluations. Unless the entire dose is administered, samples for PK measurements will not be done.

Withdrawal from the study will not affect the subject's ability to get healthcare at UHCMC.

15.3 Future Use of Stored Specimens

Subjects will be asked for permission to keep any remaining specimen for possible use in future research studies related to the development of the test article and the treatment and/or diagnosis of tuberculosis. Some samples may be stored at the local site and some at a central laboratory facility. Samples may be shared with other investigators at other institutions. The samples will not be sold or used directly for production of any commercial product. No human genetic tests will be performed on samples. Each sample will be encoded (labeled) with a barcode and a unique tracking number to protect subject's confidentiality.

There are no benefits to subjects in the collection, storage, and subsequent research use of specimens. Reports about future research done with subject's samples will not be kept in their health records. Subjects may be given the option to decide if they want their samples to be used for future research or have their samples destroyed at the end of the study. A subject's decision can be changed at any time prior to the end of the study by notifying the study doctors or nurses in writing. However, if a subject consents to future use and some of their venous blood has already been used for research purposes, the information from that research may still be used.

16 PUBLICATION POLICY

The investigators will submit to the National Library of Medicine's PubMed Central an electronic version of the final, peer-reviewed manuscripts upon acceptance for publication, to be made publicly available no later than 12 months after the official date of publication.

Since the DMID is the IND sponsor, the DMID will assume responsibility for registering the trial in the National Library of Medicine registry, www.clinicaltrials.gov. In compliance with Public Law 110-85, the Food and Drug Administration Amendments Act of 2007 (FDAAA), the DMID will also post the results of the trial in accordance to the legal requirements.

The Sponsor (NIAID) requires that any dissemination of findings shall not occur without prior submission to the Project Officer for review. The Project Officer (or designee) shall have seven (7) days from receipt of materials to review and provide comments on abstracts and 30 calendar days from receipt of materials to review and provide comments on any other publications or presentations. In addition, the TB Alliance shall review any abstracts/manuscripts before presentation/publication according to these same timelines.

17 LITERATURE REFERENCES

Andries K, Verhasselt P, Guillemont J, *et al*. A diarylquinoline drug active on the ATP synthase of *Mycobacterium turberculosis*. *Science* 2005; 307: 223-227.

Browne R. On the use of a pilot sample for sample size determination. *Stat Med* 1995; 14: 1933-1940.

Corbett EL, Watt CJ, Walker N, *et al*. The growing burden of tuberculosis: global trends and interactions with the HIV epidemic. *Arch Intern Med* 2003; 163: 1009-1021.

Diacon A, Pym A, Grobusch M, *et al*. The Diarylquinoline TMC207 for multidrug-resistant tuberculosis. *N Engl J Med* 2009; 360 (23): 2397-2405.

Food and Drug Administration. Guidance for industry: Toxicity grading scale for healthy adult and adolescent volunteers enrolled in preventive vaccine clinical trials. Obtained from http://www.fda.gov/BiologicsBloodVaccines/GuidanceComplianceRegulatoryInformation/Guidances/Vaccines/ucm074775.htm January 3, 2012.

Grimm SW, Einolf HJ, Hall SD, *et al*. The conduct of *in vitro* studies to address time-dependent inhibition of drug-metabolizing enzymes: a perspective of the pharmaceutical research and manufacturers of America. *Drug Metab Dispos* 2009; 37:1355-1370.

Huitric E, Verhasselt P, Andries K, Hoffner SE. *In vitro* antimycobacterial spectrum of a diarylquinoline ATP synthase inhibitor. *Antimicrob Agents Chemother* 2007; 51: 4202-4204.

Ibrahim M, Andries K, Lounis N, *et al*. Synergistic activity of R207910 combined with pyrazinamide against murine tuberculosis. *Antimicrob Agents Chemother* 2007; 51: 1011-1015.

Koul A, Dendouga N, Vergauwen K, *et al*. Diarylquinolines target subunit c of mycobacterial ATP synthase. *Nat Chem Biol* 2007; 3: 323-324.

Koul A, Vranckx L, Dendouga N, *et al*. Diarylquinolines are bactericidal for dormant mycobacteria as a result of disturbed homeostasis. *J Biol Chem* 2008; 283: 25273-25280.

Li AP, Reith MK, Rasmussen A, *et al*. Primary human hepatocytes as a tool for the evaluation of structure-activity relationship in cytochrome P450 induction potential of xenobiotics: evaluation of rifampin, rifabutin and rifabutin. *Chem Biol Interact* 1997; 107: 17-23.

Rustomjee R, Diacon AH, Allen J, et al. Early bactericidal activity and pharmacokinetics of the diarylquinoline TMC207 in treatment of pulmonary tuberculosis. *Antimicrob Agents Chemother* 2008 Aug; 52(8): 2831-5.

Tibotec Pharmaceuticals Ltd. TMC207 Investigator's Brochure, Edition 6. December 2010.

Wallis RS., Palaci M, Vinha, S, et al. A whole blood bactericidal assay for tuberculosis. *J Infectious Diseases 2001*; 15 April 183:000-000.

APPENDIX A: SCHEDULE OF EVENTS

Screening:

**Study Day**	**Blood sample**		**Urine sample**		**ECG**	**Vital Signs**[d]	**Other**[a]
	Drug	Safety[b]	Urinalysis	Urine Drug/ Screen			
Screening (≤ 21 days prior to Day -1 of Session I)		X	X	X	X	X	Informed consent, physical examination[c, e], family and personal medical history including history of skin disease, serum pregnancy test (if applicable), smoking habits, inclusion/exclusion criteria, concomitant diseases and/or, subject characteristics and demographics, height, weight, HIV-1 & -2 test, HBsAg, HCV antibody, Hepatitis A IgM antibody test, blood alcohol level.

[a] Adverse events (AEs) and intake of concomitant medication will be monitored throughout the trial from the signing of the informed consent form (ICF) onwards until the last trial-related activity.

[b] Serum chemistry, Hematology and Coagulation. Serum chemistry sample must be taken fasted for at least 10 hours, before breakfast, if applicable.

[c] Physical examination includes skin examination (partially disrobed).

[d] Vital signs include blood pressure, pulse, respirations, and oral temperature. Blood pressure and pulse; supine after five minutes, standing after one minute.

[e] Screening Eye examination with fundoscopy, retinal photograph, and slit lamp exam ±60 business days.

APPENDIX A: SCHEDULE OF EVENTS (cont'd)

Period 1:

Study Day	Time	Blood sample		Urine sample		ECG[e]	Vital Signs[d]	Other[a]
		Drug[h]/ WBA	Safety[b]	Urinalysis	Urine Drug Screen			
-1			X	X	X	X	X	Admission to unit ;<br>Physical examination[c];<br>Checking inclusion/exclusion criteria and review medical history;<br>Serum pregnancy test
1	-2 h					X	X	Start restriction of water intake
	-30 min							Standard breakfast in unit
	0 h	X[f,gj]						Supervised intake TMC207 400mg
	1 h	X[f,j]						
	2 h	X[f,]						Resume water intake
	3 h	X[f,j]						
	4 h	X[f,j]				X		Standard lunch
	5 h	X[f]						
	6 h	X[f,j]						
	8 h	X[f,j]						
	12 h	X[f,j]						
2	24 h	X[f]	X	X		X	X	Discharge from unit, assessments (to include physical examination if necessary[c, i])

APPENDIX A: SCHEDULE OF EVENTS (cont'd)

Period 1 (cont'd)

Study Day	Time	Blood sample		Urine sample		ECG[e]	Vital Signs[d]	Other[a]
		Drug[h]	Safety[b]	Urinalysis	Urine Drug Screen			
3	≈48 h	X[f]						Return to clinic for blood draw
4	≈72 h	X[f]						Return to clinic for blood draw
5	≈96 h	X[f]						Return to clinic for blood draw
6	≈120 h	X[f]						Return to clinic for blood draw
7	≈144 h	X[f]			X			Return to clinic for blood draw including blood alcohol level and urine drug screen
8	≈168 h	X[f]						Return to clinic for blood draw
9	≈192 h	X[f]						Return to clinic for blood draw
10	≈216 h	X[f]						Return to clinic for blood draw
11	≈240 h	X[f]						Return to clinic for blood draw
12	≈264 h	X[f]						Return to clinic for blood draw
13	≈288 h	X[f]						Return to clinic for blood draw
14	≈312 h	X[f]						Return to clinic for blood draw
15	≈336 h	X[f]	X	X	X	X	X	Return to clinic for blood draw including blood alcohol level and assessments[i] (as needed)

[a] AEs and intake of concomitant medication will be monitored throughout the trial from the signing of the ICF onwards until the last trial-related activity.

[b] Serum chemistry, hematology and coagulation. Serum chemistry sample must be taken fasted for at least 10 hours, before breakfast, if applicable.

[c] Physical examination includes skin examination (partially disrobed).

[d] Vital signs include blood pressure, pulse, respirations, and oral temperature. Blood pressure and pulse; supine after five minutes, standing after one minute.

[e] Day 1 and Day 29 ECGs within 30 minutes (±15 minutes) before start of breakfast. All other ECGs should be performed at specified time points (with appropriate windows) indicated in section 7.4.1.
[f] For determination of TMC207 and the M2 metabolite concentrations.
[g] Immediately (within 10 minutes) before intake of trial medication.
[h] For PK blood draws through 12 hours post-dose inclusive, ± 10 minutes is allowed; for later time points post-dose, ± 15 minutes is allowed. For PK blood draws at time points greater than 24 hours post dose, a window of 4 hours before/after the scheduled sampling time is allowed.
[i]Eye examination with fundoscopy and slit lamp exam ±5 business days. Retinal photographs completed at screening, day 19, day 57.
[j] Whole Blood assays ± 10 minutes

APPENDIX A: SCHEDULE OF EVENTS (cont'd)
Period 2:

Study Day	Time	Blood sample		Urine sample		ECG	Vital Signs[d]	Other[a]
		Drug[h]	Safety[b]	Urinalysis	Urine Drug Screen			
19			X	X	X			Physical examination[c,j]; Checking inclusion/exclusion criteria Blood alcohol level Serum pregnancy test
20						X	X	Supervised intake of rifabutin 300mg or rifampin 600mg;
21			X	X				Supervised intake of rifabutin 300mg or rifampin 600mg;
22								Supervised intake of rifabutin 300mg or rifampin 600mg;
23			X	X				Supervised intake of rifabutin 300mg or rifampin 600mg;
24 – 26								Supervised intake of rifabutin 300mg or rifampin 600mg;
27	Pre-dose	X[f,g,i]						Supervised intake of rifabutin 300mg or rifampin 600mg;
	2 h	X[i]						For rifampin or rifabutin drug levels

APPENDIX A: SCHEDULE OF EVENTS

Period 2 (cont'd)

Study Day	Time	Blood sample		Urine sample		ECG	Vital Signs[d]	Other[a]
		Drug[h]	Safety[b]	Urinalysis	Urine Drug Screen			
28	Pre-dose	X[f,g,i]	X	X	X	X	X	Admission to unit,<br>Blood alcohol level<br>Supervised intake of rifabutin 300mg or rifampin 600mg[c]
	2 h	X[i]						For rifampin or rifabutin drug levels
29	-2 h					X[e]	X	Start restriction of water intake
	-30 min							Standard breakfast in unit
	24 h/0 h	X[f,g,i,k]						Supervised intake of TMC207 400mg + rifabutin 300mg or rifampin 600mg (TMC207 intake within 5 minutes after rifabutin or rifampin intake)
	1 h	X[f,k]						
	2 h	X[f,i]						Resume water intake
	3 h	X[f,k]						
	4 h	X[f,k]				X[e]		Standard lunch
	5 h	X[f]						
	6 h	X[f,k]						
	8 h	X[f,k]						
	12 h	X[f,k]						

APPENDIX A: SCHEDULE OF EVENTS
Period 2 (cont'd)

Study Day	Time	Blood sample		Urine sample		ECG	Vital Signs[d]	Other[a]
		Drug[h]	Safety[b]	Urinalysis	Urine Drug Screen			
30	24/0 h	X[f,g,i]	X	X		X[e]	X	Supervised intake rifabutin 300mg or rifampin 600mg. Discharge from unit after assessments (include physical examination if necessary[c,j]);
	2 h	X[i]						
31	≈48 h	X[f,g]						Return to clinic for blood draw and supervised intake rifabutin 300mg or rifampin 600mg
32	≈72 h	X[f,g]						Return to clinic for blood draw and supervised intake rifabutin 300mg or rifampin 600mg
33	≈96 h	X[f,g]						Return to clinic for blood draw and supervised intake rifabutin 300mg or rifampin 600mg
34	≈120 h	X[f,g]						Return to clinic for blood draw and supervised intake rifabutin 300mg, 600mg or rifampin 600mg

APPENDIX A: SCHEDULE OF EVENTS Period 2 (cont'd)

**Study Day**	**Time**	**Blood sample**		**Urine sample**		**ECG**	**Vital Signs**[d]	**Other**[a]
		Drug[h]	Safety[b]	Urinalysis	Urine Drug Screen			
35	≈144 h	X[f,g,i]						Return to clinic for blood draw and supervised intake rifabutin 300mg or rifampin 600mg
	≈146 h	X[i]						
36	≈168 h	X[f,g]	X	X		X[e]	X	Return to clinic for blood draw and supervised intake rifabutin 300mg or rifampin 600mg
37	≈192 h	X[f,g]						Return to clinic for blood draw and supervised intake rifabutin 300mg or rifampin 600mg
38	≈216 h	X[f,g]						Return to clinic for blood draw and supervised intake rifabutin 300mg or rifampin 600mg
39	≈240 h	X[f,g]						Return to clinic for blood draw and supervised intake rifabutin 300mg or rifampin 600mg
40	≈264 h	X[f,g]						Return to clinic for blood draw and supervised intake rifabutin 300mg or rifampin 600mg

APPENDIX A: SCHEDULE OF EVENTS (cont'd)

Period 2 (cont'd)

Study Day	Time	Blood Samples		Urine Samples		ECG	Vital Signs[d]	Other[a]
		Drug[h]	Safety[b]	Urinalysis	Urine Drug Screen			
41	≈288 h	X[f,g,i]						Return to clinic for blood draw and supervised intake rifabutin 300mg or rifampin 600mg
41 (cont'd)	≈290 h	X[i]						
42	≈312 h	X[f]						Return to clinic for blood draw
43	≈336 h	X[f]	X	X		X[e]	X	Return to clinic for blood draw and assessments

[a]AEs and intake of concomitant medication will be monitored throughout the trial from the signing of the ICF until the last trial-related activity.
[b] Serum chemistry, hematology and coagulation. Serum chemistry sample must be taken fasted for at least 10 hours, before breakfast, if applicable.
[c] Physical examination includes skin examination (partially disrobed).
[d] Vital signs include blood pressure, pulse, respirations, and oral temperature. Blood pressure and pulse; supine after five minutes, standing after one minute.
[e] Day 1 and Day 29 ECGs within 30 minutes (±15 minutes) before start of breakfast. All other ECGs should be performed at specified time points (with appropriate windows) indicated in section 7.4.1.
[f] For determination of TMC207 and the M2 metabolite concentrations.
[g] Immediately (within 10 minutes) before intake of trial medication.
[h] For PK blood draws through 12 hours post-dose inclusive, ± 10 minutes is allowed; for later time-points post-dose, ± 15 minutes. For PK blood draws at time points greater than 24 hours post dose, a window of 3-4 hours before/after the scheduled time is allowed.
[i] For rifabutin and rifampin drug levels.
[j]Eye examination with fundoscopy and slit lamp exam ±5 business days. Retinal photographs completed at screening, day 19, day 57.
[k]Whole Blood assays ± 10 minutes

APPENDIX A: SCHEDULE OF EVENTS (cont'd)

Additional safety visits:

**Day**	**Blood sample**		**Urinalysis**	**Vital Signs**[d]	**Other**[a]
	Drug	Safety[b]			
Study Day 57 (Follow-up 28 days after last TMC207 intake in last treatment session)		X	X	X	Review of medical history, Physical examination[c,e]; Serum pregnancy test (if applicable); ECG

[a] AEs and intake of concomitant medication will be monitored throughout the trial from the signing of the ICF onwards until the last-trial related activity.
[b] Serum chemistry, hematology and coagulation. Serumchemistry sample must be taken fasted for at least 10 hours, before breakfast.
[c] Physical examination includes skin examination (partially disrobed).
[d] Vital signs include blood pressure, pulse, respirations, and oral temperature. Blood pressure and pulse; supine after five minutes, standing after one minute.
[e]Eye examination with fundoscopy, retinal photograph, and slit lamp exam ±14 business days.

APPENDIX A: SCHEDULE OF EVENTS (cont'd)

Flow chart in case of dropout other than withdrawal of consent:

Day	Blood sample		Urinalysis	Vital Signs[d]	Other[a]
	Drug	Safety[b]			
At time of dropout	X[e]	X	X	X	Physical examination[c,f]
28 days after dropout		X	X	X	Physical examination[c,f]<br>Serum pregnancy test (if applicable);<br>ECG

[a] AEs and intake of concomitant medication will be monitored throughout the trial from the signing of the ICF onwards until the last trial-related activity.
[b] Serum chemistry, hematology and coagulation. Serumchemistry sample must be taken fasted for at least 10 hours, before breakfast, if applicable. The serum chemistry sample taken at the time of dropout should preferable be taken fasted for at least 10 hours.
[c] Physical examination includes skin examination (partially disrobed).
[d] Vital signs include blood pressure, pulse, respirations, and oral temperature. Blood pressure and pulse: supine after five minutes, standing after one minute.
[e] For determination of concentrations of TMC207, the M2 metabolite, rifabutin or rifampin, if applicable.
[f]Eye examination with fundoscopy and slit lamp exam ±5 days.(Retinal photographs completed at screening, day 19, day 57)

APPENDIX B: BLOOD SAMPLING VOLUMES

Study Day	Analyte	Scheduled time Relative to TMC207 dose (hours)	Required Blood for PK/WBA (mL)	Additional blood required for safety (mL)	Total Blood Volume for the Day
Screening		-48h		18mL	18mL
-1		-24h		18mL	18mL
1	TMC207+M2<br>WBA	0h	4mL		34mL
	TMC207+M2<br>WBA	1h	4mL		
	TMC207+M2<br>WBA	2h	3mL		
	TMC207+M2<br>WBA	3h	4mL		
	TMC207+M2<br>WBA	4h	4mL		
	TMC207+M2	5h	3mL		
	TMC207+M2<br>WBA	6h	4mL		
	TMC207+M2<br>WBA	8h	4mL		
	TMC207+M2	12h	4mL		
2	TMC207+M2	24h	3mL	18mL	21mL

APPENDIX B: BLOOD SAMPLING VOLUMES (cont'd)

Study Day	Analyte	Scheduled time Relative to TMC207 dose (hours)	Required Blood for PK/WBA (mL)	Additional blood required for safety (mL)	Total Blood Volume for the Day
3	TMC207+M2	48h	3mL		3mL
4	TMC207+M2	72h	3mL		3mL
5	TMC207+M2	96h	3mL		3mL
6	TMC207+M2	120h	3mL		3mL
7	TMC207+M2	144h	3mL		3mL
8	TMC207+M2	168h	3mL		3mL
9	TMC207+M2	192h	3mL		3mL
10	TMC207+M2	216h	3mL		3mL
11	TMC207+M2	240h	3mL		3mL
12	TMC207+ M2	264h	3mL		3mL
13	TMC207+ M2	288h	3mL		3mL
14	TMC207+ M2	312h	3mL		3mL
15	TMC207+ M2	336h	3mL	18mL	21mL
19		432h		18mL	18mL
21		480h		18mL	18mL
23		528h		18mL	18mL

APPENDIX B: BLOOD SAMPLING VOLUMES (cont'd)

Study Day	Analyte	Scheduled time Relative to TMC207 dose (hours)	Required Blood for PK/WBA (mL)	Additional blood required for safety (mL)	Total Blood Volume for the Day
27	TMC207+ M2<br>AND<br>Rifabutin + desacetyl rifabutin<br>OR<br>Rifampin + desacetyl rifampin	Pre-dose	8mL		13mL
	Rifabutin+ desacetyl rifabutin<br>OR<br>Rifampin+desacetyl rifampin	2 h	5mL		
28	TMC207+ M2<br>AND<br>Rifabutin + desacetyl rifabutin<br>OR<br>Rifampin + desacetyl rifampin	Pre-dose	8mL	18mL	31mL
	Rifabutin + desacetyl rifabutin<br>OR<br>Rifampin + desacetyl rifampin	2h	5mL		

APPENDIX B: BLOOD SAMPLING VOLUMES (cont'd)

Study Day	Analyte	Scheduled time Relative to TMC207 dose (hours)	Required Blood for PK/WBA (mL)	Additional blood required for safety (mL)	Total Blood Volume for the Day
29	TMC207 + M2<br>AND<br>Rifabutin + desacetyl rifabutin<br>OR<br>Rifampin + desacetyl rifampin<br>WBA	Pre-dose or 0h	9mL		39mL
	TMC207+M2<br>WBA	1h	4mL		
	TMC207+M2<br>AND<br>Rifabutin + desacetyl rifabutin<br>OR<br>Rifampin + desacetyl rifampin<br>WBA	2h	3mL		
	TMC207+M2<br>WBA	3h	4mL		
	TMC207+M2<br>WBA	4h	4mL		
	TMC207+M2	5h	3mL		

APPENDIX B: BLOOD SAMPLING VOLUMES (cont'd)

Study Day	Analyte	Scheduled time Relative to TMC207 dose (hours)	Required Blood for PK/WBA (mL)	Additional blood required for safety (mL)	Total Blood Volume for the Day
29 (cont'd)	TMC207+M2<br>WBA	6h	4mL		
	TMC207+M2<br>WBA	8h	4mL		
	TMC207+M2	12h	4mL		
30	Rifabutin+ desacetyl rifabutin<br>OR<br>Rifampin+desacetyl rifampin	2hr	5ml		
	TMC207+M2<br>AND<br>Rifabutin+ desacetyl rifabutin<br>OR<br>Rifampin + desacetyl rifampin	24h	8mL		23mL
31	TMC207+M2	48h	3mL		3mL
32	TMC207+M2	72h	3mL		3mL
33	TMC207+M2	96h	3mL		3mL
34	TMC207+M2	120h	3mL		3mL

35	TMC207+M2<br>AND<br>Rifabutin + desacetyl rifabutin<br>OR<br>Rifampin + desacetyl rifampin	144h	8mL		13mL
	Rifabutin+ desacetyl rifabutin<br>OR<br>Rifampin + desacetyl rifampin	146h	5mL		

APPENDIX B: BLOOD SAMPLING VOLUMES (cont'd)

Study Day	Analyte	Scheduled time Relative to TMC207 dose (hours)	Required Blood for PK (mL)	Additional blood required for safety (mL)	Total Blood Volume for the Day
36	TMC207+M2	168h	3mL	18mL	21mL
37	TMC207+M2	192h	3mL		3mL
38	TMC207+M2	216h	3mL		3mL
39	TMC207+M2	240h	3mL		3mL
40	TMC207+M2	264h	3mL		3mL
41	TMC207+M2<br>AND<br>Rifabutin + desacetyl rifabutin<br>OR<br>Rifampin + desacetyl rifampin	288h	8mL		13mL

	Rifabutin+ desacetyl rifabutin<br>OR<br>Rifampin+<br>desacetyl rifampin	290h	5mL		

APPENDIX B: BLOOD SAMPLING VOLUMES (cont'd)

Study Day	Analyte	Scheduled time Relative to TMC207 dose (hours)	Required Blood for PK (mL)	Additional blood required for safety (mL)	Total Blood Volume for the Day
42	TMC207+M2	312h	3mL		3mL
43	TMC207+M2	336h	3mL	18mL	21mL
57		672h		18mL	18mL
				**Total Cumulative Blood Volume (per subject in either Group 1 or 2)**	**429mL**

APPENDIX C: TOXICITY TABLE

DMID Adult Toxicity Table, November 2007

ABBREVIATIONS: Abbreviations utilized in the Table:

ULN = Upper Limit of Normal	LLN = Lower Limit of Normal
R_x = Therapy	Req = Required
Mod = Moderate	IV = Intravenous
ADL = Activities of Daily Living	Dec = Decreased

ESTIMATING SEVERITY GRADE

For abnormalities NOT found elsewhere in the Toxicity Tables use the scale below to estimate grade of severity:

**GRADE 1**	**Mild –** Transient or mild discomfort (< 48 hours); no medical intervention/therapy required
**GRADE 2**	**Moderate –** Mild to moderate limitation in activity - some assistance may be needed; no or minimal medical intervention/therapy required
**GRADE 3**	**Severe –** Marked limitation in activity, some assistance usually required; medical intervention/therapy required, hospitalizations possible
**GRADE 4**	**Life-threatening** – Extreme limitation in activity, significant assistance required; significant medical intervention/therapy required, hospitalization, or hospice care probable

SERIOUS OR LIFE-THREATENING AEs

ANY clinical event deemed by the clinician to be serious or life threatening should be considered a grade 4 event. Clinical events considered serious or life threatening include, but are not limited to: seizures, coma, tetany, diabetic ketoacidosis, disseminated intravascular coagulation, diffuse petechiae, paralysis, acute psychosis, and severe depression.

HEMATOLOGY				
	Grade 1	Grade 2	Grade 3	Grade 4
Hemoglobin	9.5 - 10.5 gm/dL	8.0 - 9.4gm/dL	6.5 - 7.9 gm/dL	< 6.5 gm/dL
% Polymorphonuclear Leucocytes + Band Cells	> 80%	90 – 95%	>95%	
Absolute Neutrophil Count	1000-1500/mm^3	750-999/mm^3	500-749/mm^3	<500/mm^3
Platelets	75,000-99,999/mm^3	50,000-74,999/mm^3	20,000-49,999/mm^3	<20,000/mm^3
WBC increase (cells/mm^3) (FDA, 2005)	10,800 – 15,000	15,001 – 20,000	20,001 – 25,000	> 25,000
WBC decrease (cells/mm^3) (FDA, 2005)	2,500 – 3,500	1,500 – 2,499	1,000 – 1,499	<1,000
Lymphocytes Decrease - cell/mm^3 (FDA, 2005)	750 – 1,000	500 – 749	250 – 499	< 250
Abnormal Fibrinogen	Low:<br>100-200 mg/dL<br><br>High:<br>400-600 mg/dL	Low:<br><100 mg/dL<br><br>High:<br>>600 mg/dL	Low:<br>< 50 mg/dL	Fibrinogen associated with gross bleeding or with disseminated coagulation
Fibrin Split Product	20-40 mcg/mL	41-50 mcg/mL	51-60 mcg/mL	> 60 mcg/mL
Prothrombin Time (PT)	1.01 - 1.25 x ULN	1.26-1.5 x ULN	1.51 -3.0 x ULN	>3 x ULN
Activated Partial Thromboplastin (APPT)	1.01 - 1.66 x ULN	1.67 - 2.33 x ULN	2.34 - 3 x ULN	> 3 x ULN
Methemoglobin	5.0 - 9.9 %	10.0 - 14.9 %	15.0 - 19.9%	> 20.0 %

**CHEMISTRIES**				
	Grade 1	Grade 2	Grade 3	Grade 4
Hyponatremia	130-135 mEq/L	123-129 mEq/L	116-122 mEq/L	< 116 mEq/L or abnormal sodium *with* mental status changes or seizures
Hypernatremia	146-150 mEq/L	151-157 mEq/L	158-165 mEq/L	> 165 mEq/L or abnormal sodium *with* mental status changes or seizures
Hypokalemia	3.0 - 3.4 mEq/L	2.5 - 2.9 mEq/L	2.0 - 2.4 mEq/L or intensive replacement therapy or hospitalization required	< 2.0 mEq/L or abnormal potassium *with* paresis, ileus, or life-threatening arrhythmia
Hyperkalemia	5.6 - 6.0 mEq/L	6.1 - 6.5 mEq/L	6.6 - 7.0 mEq/l	> 7.0 mEq/L or abnormal potassium *with* life-threatening arrhythmia
Hypoglycemia	55-64 mg/dL	40-54 mg/dL	30-39 mg/dL	<30 mg/dL or abnormal glucose *with* mental status changes or coma

**CHEMISTRIES (continued)**

	Grade 1	Grade 2	Grade 3	Grade 4
Hyperglycemia<br>(nonfasting and no prior diabetes)	116 - 160 mg/dL	161- 250 mg/dL	251 - 500 mg/dL	> 500 mg/dL or abnormal glucose *with* ketoacidosis<br>or seizures
Hypocalcemia<br>(corrected for albumin)	8.4 - 7.8 mg/dL	7.7 - 7.0 mg/dL	6.9 - 6.1 mg/dL	< 6.1 mg/dL<br>or abnormal calcium *with* life threatening arrhythmia or tetany
Hypercalcemia<br>(correct for albumin)	10.6 - 11.5 mg/dL	11.6 - 12.5 mg/dL	12.6 - 13.5 mg/dL	> 13.5 mg/dL or abnormal calcium *with* life<br>threatening<br>arrhythmia
Hypomagnesemia	1.4 - 1.2 mEq/L	1.1 - 0.9 mEq/L	0.8 - 0.6 mEq/L	< 0.6 mEq/L or abnormal magnesium *with* life-threatening arrhythmia
Hypophosphatemia	2.0 - 2.4 mg/dL	1.5 -1.9 mg/dL or replacement Rx required	1.0 -1.4 mg/dL intensive therapy<br>or<br>hospitalization required	< 1.0 mg/dL or abnormal phosphate *with* life-threatening arrhythmia
Hyperbilirubinemia (when accompanied by any increase in other liver function test)	1.1 - <1.25 x ULN	1.25 - <1.5 x ULN	1.5 – 1.75 x ULN	> 1.75 x ULN

**CHEMISTRIES (continued)**

	Grade 1	Grade 2	Grade 3	Grade 4
Hyperbilirubinemia (when other liver function are in the normal range)	1.1 - <1.5 x ULN	1.5 - <2.0 x ULN	2.0 – 3.0 x ULN	> 3.0 x ULN
BUN	1.25 - 2.5 x ULN	2.6 - 5 x ULN	5.1 - 10 x ULN	> 10 x ULN
Hyperuricemia (uric acid)	7.5 – 10.0 mg/dL	10.1 – 12.0 mg/dL	12.1 – 15.0 mg/dL	>15.0 mg/dL
Creatinine	1.1 - 1.5 x ULN	1.6 - 3.0 x ULN	3.1 - 6 x ULN	> 6 x ULN or dialysis required

**ENZYMES**				
	Grade 1	Grade 2	Grade 3	Grade 4
AST (SGOT)	1.1 - <2.0 x ULN	2.0 – <3.0 x ULN	3.0 – 8.0 x ULN	> 8 x ULN
ALT (SGPT)	1.1 - <2.0 x ULN	2.0 – <3.0 x ULN	3.0 – 8.0 x ULN	> 8 x ULN
GGT	1.1 - <2.0 x ULN	2.0 – <3.0 x ULN	3.0 – 8.0 x ULN	> 8 x ULN
Alkaline Phosphatase	1.1 - <2.0 x ULN	2.0 – <3.0 x ULN	3.0 – 8.0 x ULN	> 8 x ULN
Amylase	1.1 - 1.5 x ULN	1.6 - 2.0 x ULN	2.1 - 5.0 x ULN	> 5.1 x ULN
Lipase	1.1 - 1.5 x ULN	1.6 - 2.0 x ULN	2.1 - 5.0 x ULN	> 5.1 x ULN

**URINALYSIS**

	Grade 1	Grade 2	Grade 3	Grade 4
Proteinuria	1+ or<br>200 mg - 1 gm loss/day	2-3+ or<br>1- 2 gm loss/day	4+ or<br>2-3.5 gm loss/day	nephrotic syndrome or<br>> 3.5 gm loss/day
Hematuria	microscopic only<br><10 rbc/hpf	gross, no clots<br>>10 rbc/hpf	gross, with or without clots, OR red blood cell casts	obstructive or required transfusion

**CARDIOVASCULAR**				
	Grade 1	Grade 2	Grade 3	Grade 4
Cardiac Rhythm		asymptomatic, transient signs, no Rx required	recurrent/persistent; symptomatic Rx required	unstable dysrhythmia; hospitalization and treatment required
Hypertension	transient increase<br>> 20 mm/Hg; no treatment	recurrent, chronic increase<br>> 20mm/Hg; treatment required	acute treatment required; outpatient treatment or hospitalization possible	end organ damage or hospitalization required

CARDIOVASCULAR (continued)

	Grade 1	Grade 2	Grade 3	Grade 4
Hypotension	transient orthostatic hypotension with heart rate increased by <20 beat/min or decreased by <10 mm Hg systolic BP; no treatment required	symptoms due to orthostatic hypotension or BP decreased by <20 mm Hg systolic; correctable with oral fluid treatment	requires IV fluids; no hospitalization required	mean arterial pressure <60mm/ Hg or end organ damage or shock; requires hospitalization and vasopressor treatment
Pericarditis	minimal effusion	mild/moderate asymptomatic effusion; no treatment	symptomatic effusion; pain; EKG changes	tamponade; pericardiocentesis or surgery required
Hemorrhage, Blood Loss	microscopic/occult	mild, no transfusion	gross blood loss; 1-2 units transfused	massive blood loss; > 3 units transfused

**GASTROINTESTINAL**				
	Grade 1	Grade 2	Grade 3	Grade 4
Nausea	mild or transient; maintains reasonable intake	moderate discomfort; intake decreased significantly; some activity limited	no significant intake; requires IV fluids	hospitalization required;
**GASTROINTESTINAL (continued)**				

	Grade 1	Grade 2	Grade 3	Grade 4
Vomiting	1 episode in 24 hours	2-5 episodes in 24 hours	>6 episodes in 24 hours or needing IV fluids	physiologic consequences requiring hospitalization or requiring parenteral nutrition
Constipation	requiring stool softener or dietary modification	requiring laxatives	obstipation requiring manual evacuation or enema	obstruction or toxic megacolon
Diarrhea	mild or transient; 3-4 loose stools/day or mild diarrhea last < 1 week	moderate or persistent; 5-7 loose stools/day or diarrhea lasting >1 week	>7 loose stools/day or bloody diarrhea; or orthostatic hypotension or electrolyte imbalance or >2L IV fluids required	hypotensive shock or physiologic consequences requiring hospitalization
Oral Discomfort/Dysphagia	mild discomfort; no difficulty swallowing	some limits on eating/drinking	eating/talking very limited; unable to swallow solid foods	unable to drink fluids; requires IV fluids

**NEUROLOGICAL**

	Grade 1	Grade 2	Grade 3	Grade 4
Neuro-Cerebellar	slight incoordination dysdiadochokinesis	intention tremor, dysmetria, slurred speech; nystagmus	locomotor ataxia	incapacitated
Psychiatric	mild anxiety or depression	moderate anxiety or depression; therapy required; change in normal routine	severe mood changes requiring therapy; or suicidal ideation; or aggressive ideation	acute psychosis requiring hospitalization; or suicidal gesture/attempt; or hallucinations
Muscle Strength	subjective weakness no objective symptoms/ signs	mild objective signs/symptoms no decrease in function	objective weakness function limited	paralysis
Paresthesia (burning, tingling, etc.)	mild discomfort; no treatment required	moderate discomfort; non-narcotic analgesia required	severe discomfort; or narcotic analgesia required with symptomatic improvement	incapacitating; or not responsive to narcotic analgesia

**NEUROLOGICAL (continued)**				
	Grade 1	Grade 2	Grade 3	Grade 4

Neuro-sensory	mild impairment in sensation (decreased sensation, e.g., vibratory, pinprick, hot/cold in great toes) in focal area or symmetrical distribution; or change in taste, smell, vision, and/or hearing	moderate impairment (mod decreased sensation, e.g., vibratory, pinprick, hot/cold to ankles) and/or joint position or mild impairment that is not symmetrical	severe impairment (decreased or loss of sensation to knees or wrists) or loss of sensation of at least mod degree in multiple different body areas (i.e., upper and lower extremities)	sensory loss involves limbs and trunk; paralysis; or seizures

**MUSCULOSKELATEL**				
	Grade 1	Grade 2	Grade 3	Grade 4
Arthralgia (joint pain)	mild pain not interfering with function	moderate pain, analgesics and/or pain interfering with function but not with activities of daily living	severe pain; pain and/or analgesics interfering with activities of daily living	disabling pain

**MUSCULOSKELATEL (continued)**				
	Grade 1	Grade 2	Grade 3	Grade 4

Arthritis	mild pain with inflammation, erythema or joint swelling – not interfering with function	moderate pain with inflammation, erythema or joint swelling – interfering with function, but not with activities of daily living	severe pain with inflammation, erythema, or joint swelling – interfering with activities of daily living	permanent and/or disabling joint destruction
Myalgia	myalgia with no limitation of activity	muscle tenderness (at other than injection site) or with moderate impairment of activity	severe muscle tenderness with marked impairment of activity	frank myonecrosis

**SKIN**				
	Grade 1	Grade 2	Grade 3	Grade 4
Mucocutaneous	erythema; pruritus	diffuse, maculopapular rash, dry desquamation	vesiculation or moist desquamation or ulceration	exfoliative dermatitis, mucous membrane involvement or erythema, multiforme or suspected Stevens-Johnson or necrosis requiring surgery
**SKIN (continued)**				

	Grade 1	Grade 2	Grade 3	Grade 4
Induration	< 15mm	15-30 mm	>30mm	
Erythema	< 15mm	15-30 mm	>30mm	
Edema	< 15mm	15-30 mm	>30mm	
Rash at Injection Site	< 15mm	15-30 mm	>30mm	
Pruritus	slight itching at injection site	moderate itching at injection extremity	itching over entire body	

RESPIRATORY

	Grade 1	Grade 2	Grade 3	Grade 4
Cough	transient; no treatment	persistent cough; treatment responsive	paroxysmal cough; uncontrolled with treatment	
Bronchospasm, Acute	transient; no treatment; 70% - 80% FEV_1 of peak flow	requires treatment; normalizes with bronchodilator; FEV_1 50% - 70% (of peak flow)	no normalization with bronchodilator; FEV_1 25% - 50% of peak flow; or retractions present	cyanosis: FEV_1 < 25% of peak flow or intubation necessary
Dyspnea	dyspnea on exertion	dyspnea with normal activity	dyspnea at rest	dyspnea requiring oxygen therapy

SYSTEMIC

	Grade 1	Grade 2	Grade 3	Grade 4
Allergic Reaction	pruritus without rash	localized urticaria	generalized urticaria; angioedema	anaphylaxis
Headache	Mild; no treatment required	transient, moderate; treatment required	severe; responds to initial narcotic therapy	intractable; requires repeated narcotic therapy
Fever: oral	37.7 - 38.5°C or 100.0 - 101.5°F	38.6 - 39.5°C or 101.6 - 102.9°F	39.6 - 40.5°C or 103 - 105°F	> 40°C or > 105°F
Fatigue	normal activity reduced < 48 hours	normal activity decreased 25- 50% > 48 hours	normal activity decreased > 50% can't work	unable to care for self

APPENDIX D: SUPPLEMENT TABLE FOR LABS & REFERENCE RANGES

The table reference below is to be used as a supplement to the DMID Adult Toxicity Table (Publish date: November, 2007) to adjust the laboratory values listed in the DMID Toxicity Tables to be consistent with University Hospital Case Medical Center lab normals. Laboratory values listed in this table will supersede the DMID table for toxicity grading of laboratory values.

**DMID 10-0043 Labs & Reference Ranges—UHCMC Lab**							
**Test Name**	**Reference Range**	**EXPANDED RANGES**	**HI-LO**	**GRADE 1**	**GRADE 2**	**GRADE 3**	**GRADE 4**
**Sodium**	136-145 meq/L		HI	146-150 meq/l	151-157 meq/l	158-165 meq/l	>165 meq/l
			LO	130-135 meq/l	123-129 meq/l	116-122 meq/l	<116 meq/l
**Potassium**	3.5-5.3 meq/L	5.4-5.5 meq/L	HI	5.6-6.0 meq/l	6.1-6.5 meq/l	6.6-7.0 meq/l	>7.0 meq/l
			LO	3.0-3.4 meq/l	2.5-2.9 meq/l	2.0-2.4 meq/l	<2.0 meq/l
**Chloride**	98-107 meq/l		HI	>107 meq/l			
			LO	<98 meq/l			
**Glucose (fasting)**	74-106 mg/dl	107-115 mg/dL	HI	116-160 mg/dl	161-250 mg/dl	251-500 mg/dl	>500 mg/dl
		65-73 mg/dL	LO	55-64 mg/dl	40-54 mg/dl	30-39 mg/dl	<30 mg/dl
**BUN**	6-23 mg/dl	24-28		1.25-2.5 x ULN (>28-58)	2.6-5 x ULN (>58-115)	5.1-10 x ULN (>115-230)	>10 x ULN (>230)
**Creatinine**	0.51-1.17 mg/dl	1.18-1.28 mg/dl	HI	1.1-1.5 x ULN (>1.28-1.80)	1.6-3.0 x ULN (1.81-3.60)	3.1-6 x ULN (3.61-7.02)	>6 x ULN or dialysis required (>7.02)

DMID 10-0043 Labs & Reference Ranges—UHCMC Lab							
**Test Name**	**Reference Range**	**EXPANDED RANGES**	**HI-LO**	**GRADE 1**	**GRADE 2**	**GRADE 3**	**GRADE 4**
**Calcium**	8.5-10.1 meq/l	10.2-10.5 meq/L	HI	10.6-11.5 meq/l	11.6-12.5 meq/l	12.6-13.5 meq/l	>13.5 meq/l
			LO	8.4-7.8 meq/l	7.7-7.0 meq/l	6.9-6.1 meq/l	<6.1 meq/l
**Phosphate**	2.5-4.9 mg/dl		HI	5.0-5.4 mg/dl	5.5-5.9 mg/dl	6.0-6.4 mg/dl	>6.4 mg/dl
			LO	2.0- 2.4 mg/dl	1.5-1.9 mg/dl	1.0-1.4 mg/dl	<1.00 mg/dl
**Liver Panel**							
**Alanine Amino Transferase (ALT).**	Females: 7-54 U/L	>54-59.3 U/L	*	1.1-<2.0 x ULN (59.4-<108)	2.0-<3.0 x ULN (108-<162)	3.0-8.0 x ULN (162-432)	>8 x ULN (>432)
	Males: 10-65 U/L	>65-71.4 U/L	*	1.1-<2.0 x ULN (71.5-<130)	2.0-<3.0 x ULN (130-<195)	3.0-8.0 x ULN (195-520)	>8 x ULN (>520)
**Alkaline Phosphatase**	33-136 U/L	>136-149.5 U/L	*	1.1-<2.0 x ULN (149.6-<272)	2.0-<3.0 x ULN (272-<408)	3.0-8.0 x ULN (408-1088)	>8 x ULN (>1088)
**Aspartate Amino Transferase (AST)**	10-37 IU/L	>37-40.6 IU/L	*	1.1-<2.0 x ULN (40.7-<74)	2.0-<3.0 x ULN (74-<111)	3.0-8.0 x ULN (111-296)	>8 x ULN (>296)
**Albumin, Serum**	3.4-5.0 g/dL		**	3.0-3.3 g/dl	2.0-2.9 g/dl	<2.0 g/dl	NA

**DMID 10-0043 Labs & Reference Ranges—UHCMC Lab**							
**Test Name**	**Reference Range**	**EXPANDED RANGES**	**HI-LO**	**GRADE 1**	**GRADE 2**	**GRADE 3**	**GRADE 4**
**Gamma Glutamyl Transferase**	Female: 5-55 IU/L	>55-60.4 IU/L	*	1.1-<2.0 x ULN (60.5-<110)	2.0-<3.0 x ULN (110-<165)	3.0-8.0 x ULN (165-440)	>8 x ULN (>440)
	Male: 15-85 IU/L	>85-93.4 IU/L	*	1.1-<2.0 x ULN (93.5-<170)	2.0-<3.0 x ULN (170-<255)	3.0-8.0 x ULN (255-680)	>8 x ULN (>680)
**Lactic Dehydrogenase, Serum**	84-246 U/L	0-220	HI	>221 U/L			
			LO	<100 U/L			
**Bilirubin, Total,Serum**	0.0-1.2 mg/dl	>1.2-1.31 mg/dL	With increase in other liver functions	1.1-<1.25 x ULN (1.32-<1.5)	1.25-<1.5 x ULN (1.5-<1.8)	1.5-1.75 x ULN (1.8-2.1)	>1.75 x ULN (>2.1)
		>1.2-1.31 mg/dL	Other liver functions are normal	1.1-<1.50 x ULN (1.32-<1.8)	1.50-<2.0 x ULN (1.8-<2.4)	2.0-3.0 x ULN (2.4-3.6)	>3.0 x ULN (>3.6)
**Total Protein**	6.4-8.2 g/dl		HI	>8.2 g/d			
			LO	<6.4 g/d			
**Creatine Kinase (CK), Serum**	Female: 0-215 IU/L		**	510-1003 IU/L	1004-1699 IU/L	1700-3399 IU/L	>3399 IU/L
	Male: 0-308 IU/L		**	585-1151	1152-1949	1950-3899	>3899 IU/L

**DMID 10-0043 Labs & Reference Ranges—UHCMC Lab**							
**Test Name**	**Reference Range**	**EXPANDED RANGES**	**HI-LO**	**GRADE 1**	**GRADE 2**	**GRADE 3**	**GRADE 4**
				IU/L	IU/L	IU/L	
**Prothrombin Time (PT)**	INR: 0.9-1.1		*	1.2-1.5 seconds	1.6-2.0 seconds	2.1-3.0 seconds	>3.0 seconds
	9.7-11.7 seconds	>11.7-11.81	*	1.01-1.25 x ULN (11.82-14.70)	1.26-1.5 x ULN (14.71-17.55)	1.51-3.0 x ULN (17.56-35.1)	>3 x ULN (>35.1)
**APTT**	25-31 seconds		*	1.01-1.66 x ULN (>31-51)	1.67-2.33 x ULN (>51-72)	2.34-3 x ULN (>72-93)	>3 x ULN (>93)
**Heme-8:**							
**Hemoglobin (HGB)**							
Female g/dL	12.0-16.0 g/dL	10.6-11.9 g/dL	**	9.5-10.5 g/dL	8.0-9.4 g/dL	6.5-7.9 g/dL	<6.5 g/dL
Male g/dL	13.5-17.5 g/dL	10.6-13.4 g/dL	**	9.5-10.5 g/dL	8.0-9.4 g/dL	6.5-7.9 g/dL	<6.5 g/dL
**Hematocrit (Packed Cell Volume)**	Female: 36.0-46.0%	GRADE THE SAME AS THE HEMOGLOBIN					
	Male: 41.0-52.0%	GRADE THE SAME AS THE HEMOGLOBIN					
**Erythrocyte count (RBC)**	Adult Female: 4.0 - 5.2 %	GRADE SAME AS					
	Adult Male: 4.5 - 5.9 %	GRADE SAME AS					

**DMID 10-0043 Labs & Reference Ranges—UHCMC Lab**							
**Test Name**	**Reference Range**	**EXPANDED RANGES**	**HI-LO**	**GRADE 1**	**GRADE 2**	**GRADE 3**	**GRADE 4**
		HEMOGLOBIN					
**Leukocyte Count (WBC)**	4.4-11.3 X10E9/L			11.0-13.0 X10E9/L	>13.0-15.0 X10E9/L	>15.0-30.0 X10E9/L	>30.0 or <1.0 X10E9/L
**Differential %**							
**Neutrophils**				DEFER TO ABSOLUTE NUMBERS			
**Immature Forms**				DEFER TO ABSOLUTE NUMBERS			
**Lymphocytes**				DEFER TO ABSOLUTE NUMBERS			
**Monocytes**				DEFER TO ABSOLUTE NUMBERS			
**Eosinophils**				DEFER TO ABSOLUTE NUMBERS			
**Basophils**				DEFER TO			

DMID 10-0043 Labs & Reference Ranges—UHCMC Lab							
**Test Name**	**Reference Range**	**EXPANDED RANGES**	**HI-LO**	**GRADE 1**	**GRADE 2**	**GRADE 3**	**GRADE 4**
				ABSOLUTE NUMBERS			
**Differential Counts**							
**Neutrophils**	1.20-7.70 X10E9/L		HI	>7800 NUMBER/CU MM			
			LO	1000-1300 NUMBER/CU MM	750-999 NUMBER/CU MM	500-749 NUMBER/CU MM	<500 NUMBER/CU MM
**Immature Forms**			*	51-365 NUMBER/CU MM	>365 NUMBER/CU MM		
**Lymphocytes**	1.20-4.80 X10E9/L		HI	6,000-7999 NUMBER/CU MM	8000-11,999 NUMBER/CU MM	12,000-20,000 NUMBER/CU MM	>20,000 NUMBER/CU MM
			LO	750 – 1,000	500 – 749	250 – 499	< 250
**Monocytes**	0.10-1.00 X10E9/L		*	>1200 NUMBER/CU MM			

**DMID 10-0043 Labs & Reference Ranges—UHCMC Lab**							
**Test Name**	**Reference Range**	**EXPANDED RANGES**	**HI-LO**	**GRADE 1**	**GRADE 2**	**GRADE 3**	**GRADE 4**
**Eosinophils**	0.00-0.70 X10E9/L	90-300	*	301-599 NUMBER/CU MM	600-899 NUMBER/CU MM	900-3000 NUMBER/CU MM	>3000 NUMBER/CU MM
**Basophils**	0.00-0.10 X10E9/L		*	>300			
**Erythrocyte Count**	4.50-5.90 X10E12/L			DERIVED VALUES			
**Troponin**	0.0-0.06 ng/ml			N/A	N/A	0.07-0.5 ng/ml	>0.5 ng/ml
**Mean Corpuscular Hemoglobin (MCH),**	26.0-34.0 pg/Cell			DERIVED VALUES			
**MCHC**	32.0-36.0 g/dL			DERIVED VALUES			
**MCV**	80-100fL						
**RDW**	11.5 - 14.5 %			DERIVED VALUES			
**Platelets**	150,000-450,000 cells/cu mm			75,000-99,999/mm^3	50,000-74,999/mm^3	20,000-49,999/mm^3	<20,0000/mm^3

DMID 10-0043 Labs & Reference Ranges—UHCMC Lab							
**Test Name**	**Reference Range**	**EXPANDED RANGES**	**HI-LO**	**GRADE 1**	**GRADE 2**	**GRADE 3**	**GRADE 4**
**Mean Platelet Volume (MPV)**	7.2-11.1 fL			DERIVED VALUE			
**Reticulocyte Count**	0.5-2.0%			DERIVED USE ABSOLUTE RETICS			
			HI	>1.8%			
**Reticulocyte Count (Absolute)**	24,100-87,700 K/cu mm		*	>87,700 K/cu mm			
**Urine Creatinine**							
**Males**	40-278 mg/dl		*	279-556 mg/dl (2x ulN)	>556 mg/dl		
**Females**	29-226 mg/dl		*	227-452 mg/dl (2x ULN)	>452 mg/dl		
**Urinalysis**			***				
**Specific gravity**	1.016-1.022		HI	>1.030			
			LO	<1.003			
**pH**	5-8						
**Protein**	Negative			1+or 200 mg - 1 gm loss/day	2-3+ or 1-2 gm loss/day	4+ or 2-3.5 gm loss/day	Nephritic syndrome or > 3.5 gm loss/day

DMID 10-0043 Labs & Reference Ranges—UHCMC Lab							
**Test Name**	**Reference Range**	**EXPANDED RANGES**	**HI-LO**	**GRADE 1**	**GRADE 2**	**GRADE 3**	**GRADE 4**
**Hematuria**	0-5 rbc/hpf		***	Microscopic only<br><10 rbc/hpf	Gross, no clots<br>>10 rbc/hpf	Gross, with or without clots, OR red blood cell casts	Obstructive or required tranfusion
**Urobilinogen**	0.2-1.0						
**Microalbumin<br>*Only if Urinalysis is reported as abnormal**	0-39						
**Glucose**	Negative						
**Ketones**	Negative						
**Bilirubin**	Negative						
**Nitrite**	Negative						
**ALL OTHER DIPSTICK ANALYTES**	Negative		***	Positive			
**Leukocytes**	0-5 WBC			>5WBC/hpf			
**Crystals (multiple types)**	None Number/hpf			>1<br>Number/hpf			
**Granular Casts**	None Number/hpf			>1<br>Number/hpf			

**DMID 10-0043 Labs & Reference Ranges—UHCMC Lab**							
**Test Name**	**Reference Range**	**EXPANDED RANGES**	**HI-LO**	**GRADE 1**	**GRADE 2**	**GRADE 3**	**GRADE 4**
**Hyaline Casts**	None Number/hpf			>1 Number/hpf			

*** Values marked with an asterisk –In these situations, laboratory results below the lower lab limit are NOT considered AEs**
**** Values marked with double asterisk –In these situations, laboratory results above the upper lab limit are NOT considered AEs**
***** Non menstruating women clean catch specimen**
! Urinary sodium is highly dependent on sodium intake and free water intake

APPENDIX E: SUMMARY OF PHARMACOKINETIC DATA AVAILABLE FOR TMC207

IB Section	Dose type	Species	Strain	Gender (# of subjects listed if applicable)	Dose (mg/kg for animals, mg for humans)	Last Sampling Time (hours)	C0h/min (μ g/mL)	C_{max} (μ g/mL)	AUC (μ g·h/mL)	$t_{1/2}$ (h)	t_{max} (h)	Cl (L/h/kg)	Vdss (L/kg)	% F_{abs}
Table 11	Single IV	Mouse	CD1	M	12.5	32	6.13	NR	14*	NR	NR	NR	NR	NR
				F	12.5	32	8.72	NR	13.9*	NR	NR	NR	NR	NR

IB Section	Dose type	Species	Strain	Gender (# of subjects listed if applicable)	Dose (mg/kg for animals, mg for humans)	Last Sampling Time (hours)	C0h/min (μg/mL)	C_{max} (μg/mL)	AUC (μg·h/mL)	$t_{1/2}$ (h)	t_{max} (h)	Cl (L/h/kg)	Vdss (L/kg)	% F_{abs}
		Rat	Sprague-Dawley	M	5	24	ND	NR	7.6*	NR	NR	NR	NR	NR
		Dog	Beagle	M	1	264	0.88	NR	5.6**	347	NR	0.1	38	NR
		Monkey	Cynomolgus	M	1	168	2.41	NR	4.2*	NR	NR	NR	NR	NR
Table 12	Single oral	Mouse	CD1	M	6.25	168	NR	0.40	5.1	47	1	NR	NR	73
				M	25		NR	1.15	20.0	59	2	NR	NR	72
				M	30		NR	2.14	2525.4	NRNR	3	NR	NR	NR
		Rat	Sprague-Dawley	M	20	24	NR	1.94	24.7*	NR	4	NR	NR	79
				M	50	336	NR	1.50	36.7	80.4	3	NR	NR	NR
				M	200		NR	3.29	132.5	116	20	NR	NR	NR
				M	800		NR	7.23	424.1	68.2	24	NR	NR	NR
				F	50		NR	1.70	89.3	141	5	NR	NR	NR
				F	200		NR	4.08	250.1	210	15	NR	NR	NR
				F	800		NR	6.54	402.9	164	24	NR	NR	NR
		Dog	Beagle	M	5	264	NR	0.48	10.1*	267	2	NR	NR	36
				M	10	1416	NR	0.91	53.8	1003	2	NR	NR	NR
		Monkey	Cynomolgus	M	5	72	NR	0.37	7.8*	NR	4.5	NR	NR	40
				F	10	4368	NR	0.7	10.5	194	4.7	NR	NR	NR

IB Section	Dose type	Species	Strain	Gender (# of subjects listed if applicable)	Dose (mg/kg for animals, mg for humans)	Last Sampling Time (hours)	C0h/min (μg/mL)	C_{max} (μg/mL)	AUC (μg·h/mL)	$t_{1/2}$ (h)	t_{max} (h)	Cl (L/h/kg)	Vdss (L/kg)	% F_{abs}
Table 14	90 day oral	Mouse	CD1	M	5	NR	NR	0.45	3.38	NR	NRNR	NR	NR	NR
				M	10	NR	NR	0.58	7.12	NR	NRNR	NR	NR	NR
				M	20	NR	NR	0.79	7.58	NR	NRNR	NR	NR	NR
				M	30	NR	NR	0.75	11.2	NR	NRNR	NR	NR	NR
				F	5	NR	NR	0.30	4.01	NR	NRNR	NR	NR	NR
				F	10	NR	NR	1.10	6.43	NR	NRNR	NR	NR	NR
				F	20	NR	NR	0.59	5.58	NR	NRNR	NR	NR	NR
				F	30	NR	NR	0.79	7.89	NR	NRNR	NR	NR	NR
Table 15	6 month daily oral	Rat	Sprague-Dawley	M	5	Day 1	NR	0.38	3.42	NR	4	NR	NR	NR
				M	10		NR	0.56	5.37	NR	2	NR	NR	NR
				M	20		NR	1.39	13.5	NR	4	NR	NR	NR
				M	20^		NR	1.14	4.29^^	NR	1-3	NR	NR	NR
				M	5	Day 176	NR	0.69	6.62	NR	4	NR	NR	NR
				M	10		NR	0.84	10.6	NR	2	NR	NR	NR
				M	20		NR	1.48	17.4	NR	4	NR	NR	NR

IB Section	Dose type	Species	Strain	Gender (# of subjects listed if applicable)	Dose (mg/kg for animals, mg for humans)	Last Sampling Time (hours)	C0h/min (μg/mL)	C_{max} (μg/mL)	AUC (μg·h/mL)	$t_{1/2}$ (h)	t_{max} (h)	Cl (L/h/kg)	Vdss (L/kg)	% F_{abs}
				M	20^		NR	1.56	9.93^^	NR	3-8	NR	NR	NR
				F	5	Day 1	NR	0.40	4.94	NR	2	NR	NR	NR
				F	10		NR	0.82	10.5	NR	4	NR	NR	NR
				F	20		NR	1.61	29.2	NR	8	NR	NR	NR
				F	20^		NR	1.71	9.70	NR	3	NR	NR	NR
				F	5	Day 176	NR	1.04	16.8	NR	2	NR	NR	NR
				F	10		NR	2.36	36.1	NR	4	NR	NR	NR
				F	20		NR	3.28	44.6	NR	4	NR	NR	NR
				F	20^		NR	2.38	23.3	NR	1-3	NR	NR	NR
June 2008 Version 5.3.1.4	6 month daily oral	Dog	Beagle	M	2.5	Day 28	NR	0.58	8.3	NR	1	NR	NR	NR
					10		NR	1.83	32.0	NR	1	NR	NR	NR
					40		NR	2.12	36.6	NR	4	NR	NR	NR
					2.5	Day 177/176	NR	1.27	22.8	NR	2	NR	NR	NR
					10		NR	2.82	51.4	NR	3	NR	NR	NR
					20a		NR	4.04	72.1	NR	6	NR	NR	NR
				F	2.5	Day 28	NR	0.99	14.0	NR	2	NR	NR	NR
					10		NR	2.48	40.2	NR	1	NR	NR	NR
					40		NR	3.61	69.4	NR	4	NR	NR	NR
					2.5	Day 177/176	NR	1.16	22.1	NR	6	NR	NR	NR
					10		NR	3.69	57.4	NR	2	NR	NR	NR
					20a		NR	4.66	102.0	NR	8	NR	NR	NR
				M	10	Day	NR	3.40	58.3	NR	2	NR	NR	NR

IB Section	Dose type	Species	Strain	Gender (# of subjects listed if applicable)	Dose (mg/kg for animals, mg for humans)	Last Sampling Time (hours)	C0h/min (μg/mL)	C_{max} (μg/mL)	AUC (μg·h/mL)	$t_{1/2}$ (h)	t_{max} (h)	Cl (L/h/kg)	Vdss (L/kg)	% F_{abs}
					40	28	NR	7.03	107.0	NR	8	NR	NR	NR
					140^		NR	17.5	181.4^^	NR	8	NR	NR	NR
					10	Day 175	NR	3.91	75.6	NR	2	NR	NR	NR
					20b		NR	5.92	119.0	NR	6	NR	NR	NR
					140^		NR	13.0	211.4^^	NR	0.5-8	NR	NR	NR
				F	10	Day 28	NR	7.99	115.0	NR	3	NR	NR	NR
					40		NR	10.30	193.0	NR	8	NR	NR	NR
					140^		NR	13.39	158.6^^	NR	2-8	NR	NR	NR
					10	Day 175	NR	3.63	63.6	NR	2	NR	NR	NR
					20		NR	6.44	121.0	NR	4	NR	NR	NR
					140^		NR	4.84	63.6^^	NR	0.5-8	NR	NR	NR
Table 16	9 month daily oral	Dog	Beagle	M	2	Day 91	NR	1.25	21.2	NR	NR	NR	NR	NR
					6		NR	3.60	65.7	NR	NR	NR	NR	NR
					18		NR	8.60	154	NR	NR	NR	NR	NR
					14c		NR	6.21	53.3^^	NR	NR	NR	NR	NR
					2	Day 182	NR	1.57	27.1	NR	NR	NR	NR	NR
					6		NR	3.35	63.0	NR	NR	NR	NR	NR
					18		NR	10.3	182	NR	NR	NR	NR	NR
					14c		NR	4.79	57.2^^	NR	NR	NR	NR	NR
					2	Day 273	NR	1.58	27.9	NR	NR	NR	NR	NR
					6		NR	3.08	60.9	NR	NR	NR	NR	NR
					18		NR	7.17	145	NR	NR	NR	NR	NR

IB Section	Dose type	Species	Strain	Gender (# of subjects listed if applicable)	Dose (mg/kg for animals, mg for humans)	Last Sampling Time (hours)	C0h/min (μg/mL)	C_{max} (μg/mL)	AUC (μg·h/mL)	$t_{1/2}$ (h)	t_{max} (h)	Cl (L/h/kg)	Vdss (L/kg)	% F_{abs}
					14c		NR	4.41	51.1^^	NR	NR	NR	NR	NR
				F	2	Day 91	NR	1.18	18.5	NR	NR	NR	NR	NR
					6		NR	3.37	57.0	NR	NR	NR	NR	NR
					18		NR	7.28	119	NR	NR	NR	NR	NR
					14c		NR	6.11	53.5^^	NR	NR	NR	NR	NR
					2	Day 182	NR	1.5	23.8	NR	NR	NR	NR	NR
					6		NR	4.48	67.6	NR	NR	NR	NR	NR
					18		NR	10.1	160	NR	NR	NR	NR	NR
					14c		NR	5.10	58.2^^	NR	NR	NR	NR	NR
					2	Day 273	NR	1.19	21.3	NR	NR	NR	NR	NR
					6		NR	3.75	59.7	NR	NR	NR	NR	NR
					18		NR	10.6	201	NR	NR	NR	NR	NR
					14c		NR	5.60	62.8^^	NR	NR	NR	NR	NR
Table 17	Daily	Rat	CD	24 day old M	5	Day 37	NR	0.40	5.54	NR	NR	NR	NR	NR
					15		NR	0.91	14.0	NR	NR	NR	NR	NR
					45		NR	3.23	46.5	NR	NR	NR	NR	NR
				60 day old M	5		NR	0.45	4.25	NR	NR	NR	NR	NR
					15		NR	1.17	13.1	NR	NR	NR	NR	NR
					45		NR	3.51	52.1	NR	NR	NR	NR	NR
				24 day old F	5		NR	0.41	6.24	NR	NR	NR	NR	NR
					15		NR	1.07	16.5	NR	NR	NR	NR	NR
					45		NR	3.98	55.0	NR	NR	NR	NR	NR

IB Section	Dose type	Species	Strain	Gender (# of subjects listed if applicable)	Dose (mg/kg for animals, mg for humans)	Last Sampling Time (hours)	C0h/min (μg/mL)	C_{max} (μg/mL)	AUC (μg·h/mL)	$t_{1/2}$ (h)	t_{max} (h)	Cl (L/h/kg)	Vdss (L/kg)	% F_{abs}
				60 day old F	5		NR	0.87	12.6	NR	NR	NR	NR	NR
					15		NR	2.46	35.6	NR	NR	NR	NR	NR
					45		NR	5.73	103	NR	NR	NR	NR	NR
Appendix 1	IV escalating (15" intervals)	Guinea pig	NR	7F	0.16-2.5	NR	NR	3.63 at 2.5mg/kg	NR	NR	NR	NR	NR	NR
	Single oral (gavage)	Dog	Beagle	4F	20	NR	NR	1.76	NR	NR	NR	NR	NR	NR
				4M	10, 40, 160	NR	NR	4.90 at 160mg/kg	18.3 at 160mg/kg	NR	NR	NR	NR	NR
	Single oral (gavage)	Rat	Sprague-Dawley	5M	50, 200, 800	7 day observation	NR	3.29 at 200mg/kg	132 at 200mg/kg	NR	NR	NR	NR	NR
	Single oral (gavage) 25mL/kg	Mouse	CD1	5M/5F	Vehicle, 50, 200, 800	14 day observation	NR	NR	NR	NR	NR	NR	NR	NR

IB Section	Dose type	Species	Strain	Gender (# of subjects listed if applicable)	Dose (mg/kg for animals, mg for humans)	Last Sampling Time (hours)	C0h/min (μg/mL)	C_{max} (μg/mL)	AUC (μg·h/mL)	$t_{1/2}$ (h)	t_{max} (h)	Cl (L/h/kg)	Vdss (L/kg)	% F_{abs}
Appendix 2	Single IV (slow bolus) 10mL/kg	Mouse	CD1	5M/5F	Vehicle, 12.5, 25, 50	14 day observation	NR	NR	NR	NR	NR	NR	NR	NR
Appendix 2	Single oral (gavage) 25mL/kg	Rat	Sprague-Dawley	5M/5F	Vehicle, 50, 200, 800	14 day observation	NR	NR	M: 132.5<br>F: 250.1	NR	NR	NR	NR	NR
Appendix 2	Single IV (slow bolus) 5mL/kg	Rat	Sprague-Dawley	5M/5F	Vehicle, 6.25, 12.5, 25	14 day observation	NR	NR	NR	NR	NR	NR	NR	NR
Appendix 3	Daily oral (gavage) 10mL/kg	Mouse	C57BL/6	5M/5F (Toxokinetic: vehicle 9M/9F, 30mg/kg 35M/35F)	Vehicle, 10, 30, 60, 100	5 days	NR	NR	M: 13.9<br>F: 10.3	NR	NR	NR	NR	NR

IB Section	Dose type	Species	Strain	Gender (# of subjects listed if applicable)	Dose (mg/kg for animals, mg for humans)	Last Sampling Time (hours)	C0h/min (μg/mL)	C_{max} (μg/mL)	AUC (μg·h/mL)	$t_{1/2}$ (h)	t_{max} (h)	Cl (L/h/kg)	Vdss (L/kg)	% F_{abs}
Appendix 3	Daily oral (gavage) 10mL/kg	Mouse	C57BL/6	10M/10F (+6M/6F toxokinetic)	Vehicle, 80, 100	5 days	NR	NR	80, 100 mg/kg: M: 20.3, 18.4 F: 13.7, 16.4	NR	NR	NR	NR	NR
Appendix 2	Single oral (gavage) 25mL/kg	Mouse	CD1	5M/5F	Vehicle, 50, 200, 800	14 day observation	NR	NR	NR	NR	NR	NR	NR	NR
Appendix 3	Daily oral (gavage) 200mL/kg	Mouse	CD1	10M/10F	Vehicle, 30, 60	28 days	NR	NR	30mg/kg: M: 13.9 F: 7.7	NR	NR	NR	NR	NR

IB Section	Dose type	Species	Strain	Gender (# of subjects listed if applicable)	Dose (mg/kg for animals, mg for humans)	Last Sampling Time (hours)	C0h/min (μg/mL)	C_{max} (μg/mL)	AUC (μg·h/mL)	$t_{1/2}$ (h)	t_{max} (h)	Cl (L/h/kg)	Vdss (L/kg)	% F_{abs}
Appendix 3	Daily oral (gavage) 10mL/kg	Mouse	CD1	10M/10F	Vehicle, 5, 10, 20, 30	13 weeks	NR	NR	5, 10, 20, 30mg/kg (Day 90): M: 3.38, 7.12, 7.58, 11.2 F: 4.01, 6.43, 5.58, 7.89	NR	NR	NR	NR	NR
Appendix 3	Single oral (gavage) 20mL/kg	Rat	Sprague-Dawley	5M	Vehicle, 50, 200, 600	7 day observation	NR	1.47, 3.78, 4.98	39.9, 164, 243	NR	NR	NR	NR	NR
Appendix 3	Daily oral (gavage) 10mL/kg			5M (+3M for other measurements)	Vehicle, 25, 100	14 days	NR	25mg/kg: 1.70 100mg/kg: 2.92	25mg/kg: 18. 100mg/kg: 63.9	NR	NR	NR	NR	NR

IB Section	Dose type	Species	Strain	Gender (# of subjects listed if applicable)	Dose (mg/kg for animals, mg for humans)	Last Sampling Time (hours)	C0h/min (μ g/mL)	C_{max} (μ g/mL)	AUC (μ g·h/mL)	$t_{1/2}$ (h)	t_{max} (h)	Cl (L/h/kg)	Vdss (L/kg)	% F_{abs}
Appendix 3	Daily oral (gavage)			10M/10F	Vehicle, 6, 12, 24	15 or 16 days	NR	NR	M: 3.9, 8.9, 17.9 F: 8.2, 17.3, 28.8	NR	NR	NR	NR	NR
Appendix 3	Oral (gavage) 0.125-2mL/kg	Rat	Sprague-dawley	2M/2F	25, 50, 100 (1x/2 weeks); 25, 50, 75 (1x/1 week); 6.25, 12.5, 25, 50 (2x/1 week); 6.25, 12.5, 25, 100 (5x/week)	1 month	NR	NR	NR	NR	NR	NR	NR	NR

IB Section	Dose type	Species	Strain	Gender (# of subjects listed if applicable)	Dose (mg/kg for animals, mg for humans)	Last Sampling Time (hours)	C0h/min (μg/mL)	C_{max} (μg/mL)	AUC (μg·h/mL)	$t_{1/2}$ (h)	t_{max} (h)	Cl (L/h/kg)	Vdss (L/kg)	% F_{abs}
Appendix 3	Twice weekly oral (gavage) 4mL/kg	Rat	Sprague-dawley	10M/10F	Vehicle, 12.5, 25, 75	1 month + 1month recovery	NR	NR	On Day 28: M: 7.7, 16.5, 35.6 F: 13.9, 37.9, 49.9	NR	NR	NR	NR	NR
Appendix 3	Weekly oral (gavage) 10mL/kg	Rat	Sprague-dawley	5M	Vehicle, 100	10 weeks	NR	NR	After 9th dose: 135.7	NR	NR	NR	NR	NR
Appendix 3	Daily oral (gavage) 5mL/kg	Rat	Sprague-dawley	20M/20F	Vehicle, 1.5, 6, 24 daily, 10 every other day	13 weeks	NR	NR	1.5, 6, 24, 10: M: 1.4, 9.3, 27.5, 12.2 F: 4.4, 20.4, 59.7, 26.6	NR	NR	NR	NR	NR

IB Section	Dose type	Species	Strain	Gender (# of subjects listed if applicable)	Dose (mg/kg for animals, mg for humans)	Last Sampling Time (hours)	C0h/min (μg/mL)	C_{max} (μg/mL)	AUC (μg·h/mL)	$t_{1/2}$ (h)	t_{max} (h)	Cl (L/h/kg)	Vdss (L/kg)	% F_{abs}
Appendix 3	Oral (gavage) 5mL/kg	Rat	CD1	20M/20F (10M/10F in recovery period)	Vehicle, 5, 10, 20 (daily); 20 (twice weekly)	6 months + 12 weeks recovery	NR	NR	NR	NR	NR	NR	NR	NR
Appendix 3	Single oral (gavage) 10mL/kg	Dog	Beagle	1M/1F	25, 100, 300, vehicle		NR	NR	25, 100, 300mg/kg: M: 32.3, 682, 165.7 F: 17.1, 226.3, 62.3	NR	NR	NR	NR	NR
	Daily oral (gavage) 5mL/kg			1M/1F	100	14 days	NR	NR	M: 110.5 F: 83.4	NR	NR	NR	NR	NR

IB Section	Dose type	Species	Strain	Gender (# of subjects listed if applicable)	Dose (mg/kg for animals, mg for humans)	Last Sampling Time (hours)	C0h/min (μg/mL)	C_{max} (μg/mL)	AUC (μg·h/mL)	$t_{1/2}$ (h)	t_{max} (h)	Cl (L/h/kg)	Vdss (L/kg)	% F_{abs}
Appendix 3	Daily oral (gavage) 5mL/kg	Dog	Beagle	3M/3F (2M/2F vehicle and high dose for recovery group)	Vehicle, 10, 40, 160	1 month + 1 month recovery	NR	NR	10, 40, 160mg/kg: M: 40.1, 63.4, 132.6 F: 48.1, 68.5, 145.2	NR	NR	NR	NR	NR
Appendix 3	Daily oral (gavage) 2.5mL/kg	Dog	Beagle	3M/3F	Vehicle, 2.5, 10, 40 (day 1-113/114) /20(Day 114/115-end)	Day 85	NR	NR	M: 15.4, 79.2, 71.3 F: 19.3, 48.8, 117.4	NR	NR	NR	NR	NR
				4M/4F		Day 176	NR	NR	M: 22.8, 51.4, 72.1 F: 22.1, 57.4, 102.0	NR	NR	NR	NR	NR

IB Section	Dose type	Species	Strain	Gender (# of subjects listed if applicable)	Dose (mg/kg for animals, mg for humans)	Last Sampling Time (hours)	C0h/min (μg/mL)	C_{max} (μg/mL)	AUC (μg·h/mL)	$t_{1/2}$ (h)	t_{max} (h)	Cl (L/h/kg)	Vdss (L/kg)	% F_{abs}
Appendix 3 (page 55)	Daily oral (gavage) 5mL/kg	Dog	Beagle	3M/3F	Vehicle, 10, 40/20 (lowered at Day 57) daily, 140 twice weekly	2 months	NR	NR		NR	NR	NR	NR	NR
				3M/3F		6 months	NR	NR	M: 75.6, 119, 1480 F: 63.3, 121, 445	NR	NR	NR	NR	NR
Appendix 3 (page 156)	Daily oral (gavage) 5mL/kg	Dog	Beagle	4M/4F	Vehicle, 2, 6, 18 (qd), 14 3x weekly	Day 273	NR	NR	M: 27.9, 60.9, 145.1, 106.7 F: 21.3, 59.7, 201.1, 131.1	NR	NR	NR	NR	NR

IB Section	Dose type	Species	Strain	Gender (# of subjects listed if applicable)	Dose (mg/kg for animals, mg for humans)	Last Sampling Time (hours)	C0h/min (μg/mL)	C_{max} (μg/mL)	AUC (μg·h/mL)	$t_{1/2}$ (h)	t_{max} (h)	Cl (L/h/kg)	Vdss (L/kg)	% F_{abs}
Appendix 4 (page 157)	Single oral (gavage) 20mL/kg	Mouse	CD1	6M	Vehicle, 37.5, 150, 600	48 hours	NR	NR	NR	NR	NR	NR	NR	NR
Appendix 4 (page 157)	Single oral (gavage) 20mL/kg	Mouse	CD1	5M/5F (+3M/3F, low+medium dose and 5M/5F high dose toxokinetic)	Vehicle, 40, 160, 640	48 hours	NR	NR	At 640mg/kg: M: 52.6 F: 42.0	NR	NR	NR	NR	NR

IB Section	Dose type	Species	Strain	Gender (# of subjects listed if applicable)	Dose (mg/kg for animals, mg for humans)	Last Sampling Time (hours)	C0h/min (μg/mL)	C_{max} (μg/mL)	AUC (μg·h/mL)	$t_{1/2}$ (h)	t_{max} (h)	Cl (L/h/kg)	Vdss (L/kg)	% F_{abs}
Appendix 5 (page 158)	Daily oral (gavage)	Rat	Sprague-Dawley	24M/24F	Vehicle, 1.5, 6, 24	NR	NR	NR	NR	NR	NR	NR	NR	NR
Appendix 5 (page 158)	Daily oral (gavage)	Rat	Sprague-Dawley	8F (pregnant)	Vehicle, 5, 15, 45	Gestation day 14	NR	At 45mg/kg: 4.62	At 45mg/kg:76.6	NR	NR	NR	NR	NR
Appendix 5 (page 158)	Daily oral (gavage)	Rat	Sprague-Dawley	24F (pregnant) + 24F for toxokinetics	Vehicle, 5, 15, 45	Gestation day 16/17	NR	2.99	50.2	NR	NR	NR	NR	NR

IB Section	Dose type	Species	Strain	Gender (# of subjects listed if applicable)	Dose (mg/kg for animals, mg for humans)	Last Sampling Time (hours)	C0h/min (μg/mL)	C_{max} (μg/mL)	AUC (μg·h/mL)	$t_{1/2}$ (h)	t_{max} (h)	Cl (L/h/kg)	Vdss (L/kg)	% F_{abs}
Appendix 5 (page 159)	Daily oral (gavage)	Rabbit	New Zealand white	3F	Vehicle, 10, 30, 90	Day 4/5	NR	At 90mg/kg: 1.46 At 300mg/kg: 3.4	At 90mg/kg: 18.6 At 300mg/kg: 54.3	NR	NR	NR	NR	NR
Appendix 5 (page 159)	Daily oral (gavage)	Rabbit	New Zealand white	6F (pregnant)	Vehicle, 30, 100, 300	Gestation day 16	NR	2.44	42.7	NR	NR	NR	NR	NR
Appendix 5 (page 160	Daily oral (gavage)	Rabbit	New Zealand white	20F (pregnant)	Vehicle, 10, 30, 100	Gestation day 19/20	NR	At 100mg/kg: 1.82	At 100mg/kg: 31.8	NR	NR	NR	NR	NR

IB Section	Dose type	Species	Strain	Gender (# of subjects listed if applicable)	Dose (mg/kg for animals, mg for humans)	Last Sampling Time (hours)	C0h/min (μ g/mL)	C_{max} (μ g/mL)	AUC (μ g·h/mL)	$t_{1/2}$ (h)	t_{max} (h)	Cl (L/h/kg)	Vdss (L/kg)	% F_{abs}
Appendix 5 (page 160	Daily oral (gavage) 5-15mL/kg	Rat	CD	6F (pregnant)	Vehicle, 5, 15, 30, 45	NR	NR	NR	NR	NR	NR	NR	NR	NR
Table 18; Appendix 5 (page 161)	Daily oral (gavage) 7.5mL/kg	Rat	CD	22F	Vehicle, 5, 15, 45	Day 6 of lactation	NR	Parent: 0.37, 1.32, 2.91 Offspring: 1.14, 3.18, 6.17	Parent: 6.09, 22.4, 53.8 Offspring: 23.4, 58.1, 120	NR	Parent: 3.00, 2.33, 2.00 Offspring: 3.67, 3.33, 3.00	NR	NR	NR
Appendix 5 (page 162)	Daily oral (gavage) 5mL/kg	Rat	CD	32M/32F	Vehicle, 5, 15, 45	Day 60	NR	NR	M: 4.25, 13.1, 52.1 F: 12.6, 35.6, 103.0	NR	NR	NR	NR	NR

IB Section	Dose type	Species	Strain	Gender (# of subjects listed if applicable)	Dose (mg/kg for animals, mg for humans)	Last Sampling Time (hours)	C0h/min (μg/mL)	C_{max} (μg/mL)	AUC (μg·h/mL)	$t_{1/2}$ (h)	t_{max} (h)	Cl (L/h/kg)	Vdss (L/kg)	% F_{abs}
Appendix 7 (page 164)	Daily oral (gavage) 10mL/kg	Rat	Wistar	19M/19F	Vehicle, 6, 20, 60	One month	NR	NR	M: 5.0, 18.5, 40.5<br>F: 10.2, 38.0, 69.2	NR	NR	NR	NR	NR
Appendix 7 (page 165)	Daily oral (gavage) 10mL/kg	Rat	Wistar	8M/8F	Vehicle, 6, 20, 60	NR	NR	NR	NR	NR	NR	NR	NR	NR
Note: For human studies doses listed are mg. Concentrations and AUCs listed are ng/mL and ng×h/mL, respectively.														
Table 20 & page 167	Single oral	Healthy human		6M/group	10	<300	NR	68.6±14.8 (SD)	AUC_{144} 1248±233 (SD)<br>AUC_{∞} 1700±291 (SD)	162±84 (SD)	6.3±0.8 (SD)	NR	NR	NR
					30		NR	276±64	4418±1424<br>6052±1861	143±31	5.0±0	NR	NR	NR

IB Section	Dose type	Species	Strain	Gender (# of subjects listed if applicable)	Dose (mg/kg for animals, mg for humans)	Last Sampling Time (hours)	C0h/min (μg/mL)	C_{max} (μg/mL)	AUC (μg·h/mL)	$t_{1/2}$ (h)	t_{max} (h)	Cl (L/h/kg)	Vdss (L/kg)	% F_{abs}
					100		NR	854±283	13604±5115	18134±6577<br>135±24	4.7±1.4	NR	NR	NR
					300		NR	2547±1305	38737±14584	53113±17911<br>169±19	4.2±1.7	NR	NR	NR
					450		NR	3755±1165	64530±26927	79179±31794<br>117±19	4.5±1.2	NR	NR	NR
					700		NR	6747±2210	97816±38074	133125±44913<br>172±37	5.2±0.4	NR	NR	NR
Table 21	Single and multiple oral dose	Healthy human (fed)		6M/group	50	Day 1	C_{24h} 63.4±10.0	428±112	3989±830	NA	5.3±0.5	NR	NR	NR
					150		180±53.0	1132±401	9922±3199	NA	5.0±0	NR	NR	NR
					400		512±114	3005±493	27206±5361	NA	3.7±1.5	NR	NR	NR

IB Section	Dose type	Species	Strain	Gender (# of subjects listed if applicable)	Dose (mg/kg for animals, mg for humans)	Last Sampling Time (hours)	C0h/min (μg/mL)	C_{max} (μg/mL)	AUC (μg·h/mL)	$t_{1/2}$ (h)	t_{max} (h)	Cl (L/h/kg)	Vdss (L/kg)	% F_{abs}
					50	Day 14	187±44	590±116	7914±2009	169±77	5.2±0.4	NR	NR	NR
					150		604±147	1972±559	24265±5670	167±48	5.0±0	NR	NR	NR
					400		1280±309	4298±1315	51525±10123	173±35	5.0±1.1	NR	NR	NR
Table 22	Daily	Humans with Tb		13	25	Day 7	98.3±36.8	319±97.7	3973±1238	NR	4.0±2.0-6.0	NR	NR	NR
				14	100		380±165	1208±395	16050±5076	NR	4.0±2.0-8.0	NR	NR	NR
				13	400		1448±437	5502±2695	64750±20700	NR	4.0±2.0-6.0	NR	NR	NR
Table 25	Daily	Humans with Tb		12-19?	400qd (2wks) + +1200E	Week 2	396.9±291.3	3210±1322	12h 19740±6946; 24h 24240±4606	NR	NR	NR	NR	NR

IB Section	Dose type	Species	Strain	Gender (# of subjects listed if applicable)	Dose (mg/kg for animals, mg for humans)		Last Sampling Time (hours)	C0h/min (μg/mL)	C_{max} (μg/mL)	AUC (μg·h/mL)	$t_{1/2}$ (h)		t_{max} (h)	Cl (L/h/kg)	Vdss (L/kg)	% F_{abs}
					200tiw (next 6 wks)	+1000KAN		NR	40550±12090	182600±49950	192500±52480	NR	NR	NR	NR	NR
						+1500Z		9.774±5.118	42.81±12.57	380.6±119.8	605.5±229.0	NR	NR	NR	NR	NR
						+600OFL		0.4423±0.5608	6.741±1.791	50.23±14.25	73.97±21.89	NR	NR	NR	NR	NR
Table 28	Single capsule	Healthy humans (fed)		12M	100		240 hours	NR	708.2±207.9	16710±5815	NR		6.0(3.0-8.0)	NR	NR	NR

IB Section	Dose type	Species	Strain	Gender (# of subjects listed if applicable)	Dose (mg/kg for animals, mg for humans)	Last Sampling Time (hours)	C0h/min (μg/mL)	C_{max} (μg/mL)	AUC (μg·h/mL)	$t_{1/2}$ (h)	t_{max} (h)	Cl (L/h/kg)	Vdss (L/kg)	% F_{abs}
		Healthy humans (fasted)					NR	370.1± 91.44	12740±4 383	NR	8.0(4 .0- 8.0)	NR	NR	NR
	Single tablet	Healthy humans (fed)		12M	100	240 hours	NR	732.8± 180.7	17580±4 944	NR	4.5(3 .0- 6.0)	NR	NR	NR
		Healthy humans (fasted)					NR	282.5± 90.20	9361±36 04	NR	8.0(4 .0- 12.0)	NR	NR	NR
Tabl e 30	Phase II tablet	Healthy humans (fed)		12M	100	672	NR	1153± 330.8	27730±1 2540	NR	5.5(2 .0- 6.0)	NR	NR	NR
		Healthy humans (fasted)					NR	323.2± 138.6	11920±5 679	NR	8.0(5 .0- 8.0)	NR	NR	NR
	Filmcoat ed fine tablet	Healthy humans (fed)		12M			NR	1037± 317.2	26450±1 0010	NR	4.0(2 .0- 6.0)	NR	NR	NR
		Healthy humans (fasted)					NR	303.7± 174.4	11450±6 481	NR	8.0(6 .0- 8.0)	NR	NR	NR

IB Section	Dose type	Species	Strain	Gender (# of subjects listed if applicable)	Dose (mg/kg for animals, mg for humans)	Last Sampling Time (hours)	C0h/min (μg/mL)	C_{max} (μg/mL)	AUC (μg·h/mL)	$t_{1/2}$ (h)	t_{max} (h)	Cl (L/h/kg)	Vdss (L/kg)	% F_{abs}
	Filmcoated course tablet	Healthy humans (fed)		12M			NR	927.3±448.4	22270±9036	NR	5.5(3.0-6.0)	NR	NR	NR
		Healthy humans (fasted)					NR	199.6±62.61	7987±1917	NR	8.0(6.0-12.0)	NR	NR	NR
Table 32	Oral solution	Healthy humans (fed)		12M	300	144	NR	2613±826	40889±14163	135±51	4.8±1.1	NR	NR	NR
		Healthy humans (fasted)		12M			NR	1665±665	32893±13362	115±42	5.7±1.7	NR	NR	NR
Table 33	Single oral	Healthy humans		16M	300	336	NR	1004	29149	NR	NR	NR	NR	NR
	Single oral + daily rifampin			16M	300 TMC207 + ? rifampin		NR	572	14041	NR	NR	NR	NR	NR
Table 34	Daily oral	Healthy humans		22M	400	Session 2, Days	1062±342.4	4408±1532	51360±15750	NR	5.0(3.0-6.0)	NR	NR	NR

IB Section	Dose type	Species	Strain	Gender (# of subjects listed if applicable)	Dose (mg/kg for animals, mg for humans)	Last Sampling Time (hours)	C0h/min (μg/mL)	C_{max} (μg/mL)	AUC (μg·h/mL)	$t_{1/2}$ (h)	t_{max} (h)	Cl (L/h/kg)	Vdss (L/kg)	% F_{abs}
	Daily oral + daily H/Z			22M	400 TMC207 + 300/2000 H/Z	10 and 15	971.2±289.7	4115±1316	44790±13690	NR	5.0(3.0-6.0)	NR	NR	NR
Table 37	Daily	Healthy humans		15M	400qd	Session 2 Days 11 and 14	1405±572.6	6400±2096	77740±26770	NR	5.0(2.0-6.0)	NR	NR	NR
	Daily+ketoconazole			15M	400qd TMC207 400qd ketoconazole		1890±831.8	7467±4014	99910±52130	NR	5.0(4.0-8.0)	NR	NR	NR
Table 40	Single	Healthy humans		16?	400	336	NR	3299±692.6	62390±12670	NR	5.0(2.0-6.0)	NR	NR	NR
	Single + LPV/rtv bid			13?	400 TMC207 + 100/100 LPV/rtv		NR	3274±688.9	75420±20740	NR	5.0(2.0-6.0)	NR	NR	NR

IB Section	Dose type	Species	Strain	Gender (# of subjects listed if applicable)	Dose (mg/kg for animals, mg for humans)	Last Sampling Time (hours)	C0h/min (μg/mL)	C_{max} (μg/mL)	AUC (μg·h/mL)	$t_{1/2}$ (h)	t_{max} (h)	Cl (L/h/kg)	Vdss (L/kg)	% F_{abs}
Table 44	Single	HIV-1 infected humans		16?	400	336	NR	5131±1901	74220±18010	NR	5.0 (3.0-6.0)	NR	NR	NR
	Single + NVP bid + 2 N(t)RTIs			16?	400 TMC207 + 200 NVP		NR	4562±2114	79440±23590	NR	5.0 (5.0-8.0)	NR	NR	NR

NR=not reported in the Investigator's Brochure
*Value likely underestimated because of too short sampling time period
**AUC: 0-264 hours
^Intermittent dosing (twice weekly)
^^AUC determined over 168h and divided by 7
a=dose reduced from 40 to 20mg/kg on Day 115/114
b=dose reduced from 40 to 20 mg/kg at 2 months
c=intermittent dosing (three times per week)

APPENDIX F: SUMMARY OF PHARMACOKINETIC DATA AVAILABLE FOR M2

IB Section	Dose type	Species	Strain	Gender (# of subjects listed if applicable)	Dose (mg/kg for animals, mg for humans)	Last Sampling Time (hours)	C0h/min (μ g/mL)	C_{max} (μ g/mL)	AUC (μ g·h/mL)	t1/2 (h)	t_{max} (h)	Cl (L/h/kg)	Vdss (L/kg)	% F_{abs}
Table 13	Single oral	Mouse	CD1	M	30	168	NR	0.97	83.3	70	24	NR	NR	NR
		Dog	Beagle	M	10	1416	NR	0.12	33.5	318	6.7	NR	NR	NR
		Monkey	Cynomolgus	F	10	4368	NR	0.11	6.8	42	6.7	NR	NR	NR
Table 14	90 day oral	Mouse	CD1	M	5	NR	NR	0.53	11.7	NR	8	NR	NR	NR
					10	NR	NR	1.23	26.6	NR	8	NR	NR	NR
					20	NR	NR	2.27	47.8	NR	8	NR	NR	NR
					30	NR	NR	3.63	70.8	NR	8	NR	NR	NR
				F	5	NR	NR	0.38	8.14	NR	3	NR	NR	NR
					10	NR	NR	1.15	17.9	NR	1	NR	NR	NR
					20	NR	NR	1.23	27.1	NR	8	NR	NR	NR
					30	NR	NR	2.22	43.0	NR	8	NR	NR	NR
Table 15	6 month oral	Rat	Sprague-Dawley	M	5	Day 1	NR	0.068	1.25	NR	8	NR	NR	NR
					10	Day 1	NR	0.15	2.74	NR	8	NR	NR	NR
					20	Day 1	NR	0.24	4.38	NR	8	NR	NR	NR
					20^	Day 1	NR	0.32	4.14	NR	8	NR	NR	NR

IB Section	Dose type	Species	Strain	Gender (# of subjects listed if applicable)	Dose (mg/kg for animals, mg for humans)	Last Sampling Time (hours)	C0h/min (μ g/mL)	C_{max} (μ g/mL)	AUC (μ g·h/mL)	t1/2 (h)	t_{max} (h)	Cl (L/h/kg)	Vdss (L/kg)	% F_{abs}
					5	Day 176	NR	0.27	5.07	NR	4	NR	NR	NR
					10	Day 176	NR	0.72	14.1	NR	8	NR	NR	NR
					20	Day 176	NR	1.04	21.8	NR	4	NR	NR	NR
					20^	Day 176	NR	0.54	8.69	NR	8-24	NR	NR	NR
				F	5	Day 1	NR	0.052	0.99	NR	24	NR	NR	NR
					10	Day 1	NR	0.097	1.71	NR	8	NR	NR	NR
					20	Day 1	NR	0.38	4.97	NR	24	NR	NR	NR
					20^	Day 1	NR	0.22	3.77	NR	24	NR	NR	NR
					5	Day 176	NR	0.49	8.85	NR	4	NR	NR	NR
					10	Day 176	NR	0.96	20.0	NR	4	NR	NR	NR
					20	Day 176	NR	1.80	36.0	NR	4	NR	NR	NR

IB Section	Dose type	Species	Strain	Gender (# of subjects listed if applicable)	Dose (mg/kg for animals, mg for humans)	Last Sampling Time (hours)	C0h/min (μ g/mL)	C_{max} (μ g/mL)	AUC (μ g·h/mL)	t1/2 (h)	t_{max} (h)	Cl (L/h/kg)	Vdss (L/kg)	% F_{abs}
					20^	Day 176	NR	0.56	9.89^^	NR	24	NR	NR	NR
Table 17	Daily	Rat	CD	24 day old M	5	Day 37	NR	0.033	0.64	NR	NR	NR	NR	NR
					15		NR	0.10	1.93	NR	NR	NR	NR	NR
					45		NR	0.31	6.00	NR	NR	NR	NR	NR
				60 day old M	5		NR	0.16	2.61	NR	NR	NR	NR	NR
					15		NR	0.55	10.5	NR	NR	NR	NR	NR
					45		NR	1.56	34.1	NR	NR	NR	NR	NR
				24 day old F	5		NR	0.039	0.73	NR	NR	NR	NR	NR
					15		NR	0.10	2.02	NR	NR	NR	NR	NR
					45		NR	0.36	6.91	NR	NR	NR	NR	NR
				60 day old F	5		NR	0.20	4.66	NR	NR	NR	NR	NR
					15		NR	0.75	16.3	NR	NR	NR	NR	NR
					45		NR	2.43	55.0	NR	NR	NR	NR	NR
June 2008 version 5.3.1.4	6 month daily oral	Dog	Beagle	M	2.5	Day 28	NR	0.47	10.5	NR	4	NR	NR	NR
					10		NR	1.64	35.8	NR	7	NR	NR	NR
					40		NR	1.81	35.8	NR	5	NR	NR	NR
					2.5	Day 177/176	NR	0.71	14.2	NR	4	NR	NR	NR
					10		NR	1.61	33.1	NR	8	NR	NR	NR
					20a		NR	1.89	38.1	NR	.	NR	NR	NR
				F	2.5	Day 28	NR	0.64	14.1	NR	5	NR	NR	NR
					10		NR	2.54	56.3	NR	6	NR	NR	NR

IB Section	Dose type	Species	Strain	Gender (# of subjects listed if applicable)	Dose (mg/kg for animals, mg for humans)	Last Sampling Time (hours)	C0h/min (μ g/mL)	C_{max} (μ g/mL)	AUC (μ g·h/mL)	t1/2 (h)	t_{max} (h)	Cl (L/h/kg)	Vdss (L/kg)	% F_{abs}
					40		NR	4.72	101.3	NR	6	NR	NR	NR
					2.5	Day 177/176	NR	0.81	17.5	NR	3	NR	NR	NR
					10		NR	2.06	42.7	NR	4	NR	NR	NR
					20a		NR	2.28	51.3	NR	16	NR	NR	NR
				M	10	Day 28	NR	1.96	42.4	NR	6	NR	NR	NR
					40		NR	3.10	58.8	NR	7	NR	NR	NR
					140^		NR	3.66	72.4^^	NR	8	NR	NR	NR
					10	Day 175	NR	2.06	45.0	NR	8	NR	NR	NR
					20b		NR	3.43	75.5	NR	5	NR	NR	NR
					140^		NR	3.14	59.6^^	NR	0.5-96	NR	NR	NR
				F	10	Day 28	NR	4.94	94.9	NR	11	NR	NR	NR
					40		NR	4.31	89.1	NR	7	NR	NR	NR
					140^		NR	3.46	68.7^^	NR	2-72	NR	NR	NR
					10	Day 175	NR	2.66	60.5	NR	6	NR	NR	NR
					20		NR	2.31	51.4	NR	7	NR	NR	NR
					140^		NR	1.77	34.3^^	NR	8-24	NR	NR	NR
Table 16	9 month daily oral	Dog	Beagle	M	2	Day 91	NR	0..8	8.15	NR	NR	NR	NR	NR
					6		NR	1.18	24.8	NR	NR	NR	NR	NR
					18		NR	3.00	63.9	NR	NR	NR	NR	NR
					14c		NR	1.80	33.0^^	NR	NR	NR	NR	NR
					2	Day 182	NR	0.51	11.0	NR	NR	NR	NR	NR
					6		NR	1.32	28.9	NR	NR	NR	NR	NR

IB Section	Dose type	Species	Strain	Gender (# of subjects listed if applicable)	Dose (mg/kg for animals, mg for humans)	Last Sampling Time (hours)	C0h/min (μ g/mL)	C_{max} (μ g/mL)	AUC (μ g·h/mL)	t1/2 (h)	t_{max} (h)	Cl (L/h/kg)	Vdss (L/kg)	% F_{abs}
					18		NR	2.06	49.0	NR	NR	NR	NR	NR
					14c		NR	1.74	36.8^^	NR	NR	NR	NR	NR
					2	Day 273	NR	0.50	11.7	NR	NR	NR	NR	NR
					6		NR	1.45	27.8	NR	NR	NR	NR	NR
					18		NR	2.95	43.5	NR	NR	NR	NR	NR
					14c		NR	1.56	33.0^^	NR	NR	NR	NR	NR
				F	2	Day 91	NR	0.69	10.2	NR	NR	NR	NR	NR
					6		NR	1.67	31.0	NR	NR	NR	NR	NR
					18		NR	2.70	63.7	NR	NR	NR	NR	NR
					14c		NR	1.80	29.0^^	NR	NR	NR	NR	NR
					2	Day 182	NR	0.65	15.2	NR	NR	NR	NR	NR
					6		NR	2.00	34.9	NR	NR	NR	NR	NR
					18		NR	3.13	55.4	NR	NR	NR	NR	NR
					14c		NR	2.11	34.7^^	NR	NR	NR	NR	NR
					2	Day 273	NR	NR	13.2	NR	NR	NR	NR	NR
					6		NR	NR	40.5	NR	NR	NR	NR	NR
					18		NR	NR	62.4	NR	NR	NR	NR	NR
					14c		NR	NR	39.9^^	NR	NR	NR	NR	NR

IB Section	Dose type	Species	Strain	Gender (# of subjects listed if applicable)	Dose (mg/kg for animals, mg for humans)	Last Sampling Time (hours)	C0h/min (μ g/mL)	C_{max} (μ g/mL)	AUC (μ g·h/mL)	t1/2 (h)	t_{max} (h)	Cl (L/h/kg)	Vdss (L/kg)	% F_{abs}
Appendix 3	Daily oral (gavage) 10mL/kg	Mouse	C57BL/6	5M/5F (Toxokinetic: vehicle 9M/9F, 30mg/kg 35M/35F)	Vehicle, 10, 30, 60, 100	5 days	NR	NR	M: 82.6 F: 67.6	NR	NR	NR	NR	NR
Appendix 3	Daily oral (gavage) 10mL/kg	Mouse	C57BL/6	10M/10F (+6M/6F toxokinetic)	M2: Vehicle, 100, 130	5 days	NR	NR	M2 100mg/kg:M: 172.0 F: 108.0	NR	NR	NR	NR	NR
Appendix 3	Daily oral (gavage) 10mL/kg	Mouse	C57BL/6	10M/10F (+6M/6F toxokinetic)	Vehicle, 80, 100	5 days	NR	NR	80, 100 mg/kg: M: 92.9, 119.0 F: 69.5, 78.8	NR	NR	NR	NR	NR

IB Section	Dose type	Species	Strain	Gender (# of subjects listed if applicable)	Dose (mg/kg for animals, mg for humans)	Last Sampling Time (hours)	C0h/min (μ g/mL)	C_{max} (μ g/mL)	AUC (μ g·h/mL)	t1/2 (h)	t_{max} (h)	Cl (L/h/kg)	Vdss (L/kg)	% F_{abs}
June 2008 version Appendix 2 (page 100)	Daily oral (gavage) 200mL/kg	Mouse	CD1	10M/10F	Vehicle, 30, 60	28 days	NR	NR	M: 61.8 F: 34.3	NR	NR	NR	NR	NR
June 2008 version Appendix 2 (page 100)	Daily oral (gavage) 10mL/kg	Mouse	CD1	10M/10F	Vehicle, 5, 10, 20, 30	13 weeks	NR	NR	5, 10, 20, 30mg/kg (Day 90): M: 11.7, 26.6, 47.8, 70.8 F: 8.14, 17.9, 27.1, 43.0	NR	NR	NR	NR	NR

IB Section	Dose type	Species	Strain	Gender (# of subjects listed if applicable)	Dose (mg/kg for animals, mg for humans)	Last Sampling Time (hours)	C0h/min (μg/mL)	C_{max} (μg/mL)	AUC (μg·h/mL)	t1/2 (h)	t_{max} (h)	Cl (L/h/kg)	Vdss (L/kg)	% F_{abs}
	Daily oral (gavage) 5mL/kg	Rat	Sprague-dawley	20M/20F	Vehicle, 1.5, 6, 24, 10 (eod)	13 weeks	NR	NR	1.5, 6, 24, 10: M: 1.7, 10.8, 29.2, 16.4 F: 2.2, 9.5, 60.1, 16.9	NR	NR	NR	NR	NR
Appendix 3	Daily oral (gavage) 2.5mL/kg	Dog	Beagle	3M/3F	Vehicle, 2.5, 10, 40 (day 1-113/114)/ 20(Day 114/115-end)	Day 85	NR	NR	M: 13.7, 61.0, 49.8 F: 18.9, 63.7, 109.2	NR	NR	NR	NR	NR
				4M/4F		Day 176	NR	NR	M: 14.2, 33.1, 38.1 F: 17.5, 42.7, 51.3	NR	NR	NR	NR	NR

IB Section	Dose type	Species	Strain	Gender (# of subjects listed if applicable)	Dose (mg/kg for animals, mg for humans)	Last Sampling Time (hours)	C0h/min (μg/mL)	C_{max} (μg/mL)	AUC (μg·h/mL)	t1/2 (h)	t_{max} (h)	Cl (L/h/kg)	Vdss (L/kg)	% F_{abs}
Appendix 3 (page 55)	Daily oral (gavage) 5mL/kg	Dog	Beagle	3M/3F	Vehicle, 10, 40/20 (lowered at Day 57) daily, 140 twice weekly	2 months	NR	NR		NR	NR	NR	NR	NR
				3M/3F		6 months	NR	NR	M: 45, 75.5, 417 F: 60.5, 51.4, 240	NR	NR	NR	NR	NR
Appendix 3 (page 156)	Daily oral (gavage) 5mL/kg	Dog	Beagle	4M/4F	Vehicle, 2, 6, 18 (qd), 14 3x weekly	Day 273	NR	NR	M: 11.7, 27.8, 43.5, 67.6 F: 13.3, 40.5, 62.4, 81.4	NR	NR	NR	NR	NR
Appendix 5 (page 158)	Daily oral (gavage)	Rat	Sprague-Dawley	8F (pregnant)	Vehicle, 5, 15, 45	Gestation day 14	NR	At 45mg/kg: 1.50	At 45mg/kg: 28.8	NR	NR	NR	NR	NR

IB Section	Dose type	Species	Strain	Gender (# of subjects listed if applicable)	Dose (mg/kg for animals, mg for humans)	Last Sampling Time (hours)	C0h/min (μg/mL)	C_{max} (μg/mL)	AUC (μg·h/mL)	t1/2 (h)	t_{max} (h)	Cl (L/h/kg)	Vdss (L/kg)	% F_{abs}
Appendix 5 (page 158)	Daily oral (gavage)	Rat	Sprague-Dawley	24F (pregnant) + 24F for toxokinetics	Vehicle, 5, 15, 45	Gestation day 16/17	NR	0.56	12.1	NR	NR	NR	NR	NR
Appendix 5 (page 159)	Daily oral (gavage)	Rabbit	New Zealand white	3F	Vehicle, 10, 30, 90	Day 4/5	NR	At 90mg/kg: 0.28 At 300mg/kg: 0.73	At 90mg/kg: 6.1 At 300mg/kg: 14.5	NR	NR	NR	NR	NR
Appendix 5 (page 159)	Daily oral (gavage)	Rabbit	New Zealand white	6F (pregnant)	Vehicle, 30, 100, 300	Gestation day 16	NR	0.73	13.2	NR	NR	NR	NR	NR

IB Section	Dose type	Species	Strain	Gender (# of subjects listed if applicable)	Dose (mg/kg for animals, mg for humans)	Last Sampling Time (hours)	C0h/min (μg/mL)	C_{max} (μg/mL)	AUC (μg·h/mL)	t1/2 (h)	t_{max} (h)	Cl (L/h/kg)	Vdss (L/kg)	% F_{abs}
Appendix 5 (page 160	Daily oral (gavage)	Rabbit	New Zealand white	20F (pregnant)	Vehicle, 10, 30, 100	Gestation day 19/20	NR	At 100mg/kg: 0.46	At 100mg/kg: 8.7	NR	NR	NR	NR	NR
Table 18; Appendix 5 (page 161)	Daily oral (gavage) 7.5mL/kg	Rat	CD	22F	Vehicle, 5, 15, 45	Day 6 of lactation	NR	Parent: 0.13, 0.49, 2.13 Offspring: 0.24, 0.65, 1.15	Parent: 2.59, 11.2, 44.6 Offspring: 5.26, 13.5, 25.6	NR	Parent: 8.00, 3.67, 2.67	NR	NR	NR
Appendix 5 (page 162)	Daily oral (gavage) 5mL/kg	Rat	CD	32M/32F	Vehicle, 5, 15, 45	Day 60	NR	NR	M: 2.61, 10.5, 34.1 F: 4.66, 16.3, 55.0	NR	NR	NR	NR	NR
Note: for human studies, doses listed are in mg. Concentrations and AUCs listed are ng/mL and ng×h/mL, respectively.														
Table 22	Daily	Humans with Tb		13?	25	Day 7	16.8±6.15	22.8±8.87	484±169	NR	12.0±0.0-24	NR	NR	NR

IB Section	Dose type	Species	Strain	Gender (# of subjects listed if applicable)	Dose (mg/kg for animals, mg for humans)	Last Sampling Time (hours)	C0h/min (μg/mL)	C_{max} (μg/mL)	AUC (μg·h/mL)	t1/2 (h)	t_{max} (h)	Cl (L/h/kg)	Vdss (L/kg)	% F_{abs}
				14?	100		58.5±13.5	80.0±20.3	1706±401	NR	10±0.0-24	NR	NR	NR
				13?	400		281±91.7	379±127	8001±2488	N	8.0±0.0-24	N	N	N
T	Single oral			16 M	300			38	6471	NR		NR	NR	NR
	Single oral + daily rifampin	Healthy humans		16M	300 TMC207 + ? rifampin	3		49	9321	NR		NR	NR	NR
Table 34	Daily oral	Healthy humans		22M	400	Session 2,	269.4±58.3	427.2±100.9	7858±1840	NR	8.0(4.0-8.0)	NR	NR	NR

IB Section	Dose type	Species	Strain	Gender (# of subjects listed if applicable)	Dose (mg/kg for animals, mg for humans)	Last Sampling Time (hours)	C0h/min (μg/mL)	C_{max} (μg/mL)	AUC (μg·h/mL)	t1/2 (h)	t_{max} (h)	Cl (L/h/kg)	Vdss (L/kg)	% F_{abs}
	Daily oral + daily H/Z			22M	400 TMC207 + 300/2000 H/Z	Days 10 and 15	333.9 ±69.0	545.7±121.6	10130±2063	NR	24.0 (0.0-24)	NR	NR	NR
Table 37	Daily	Healthy humans		15M	400qd	Session 2 Days 11 and 14	224.5 ±57.6	355.7±95.1	7086±1713	NR	6.0 (5.0-12.0)	NR	NR	NR
	Daily+ketoconazole			15M	400qd TMC207 400qd ketoconazole		244.9 ±73.2	369.9±137.0	7333±2427	NR	8.0 (0.0-12.0)	NR	NR	NR
Table 41	Single	Healthy humans		16?	400	336	NR	44.56±10.84	8135±2758	NR	8.0 (6.0-24.0)	NR	NR	NR
	Single + LPV/rtv bid			13?	400 TMC207 + 100/100 LPV/rtv		NR	21.19±7.279	4374±1369	NR	12.0 (8.0-168.0)	NR	NR	NR
Table 45	Single	HIV-1 infected humans		16?	400	336	NR	44.6±10.15	8477±1939	NR	12.0 (12.0-48.0)	NR	NR	NR

IB Section	Dose type	Species	Strain	Gender (# of subjects listed if applicable)	Dose (mg/kg for animals, mg for humans)	Last Sampling Time (hours)	C0h/min (μg/mL)	C_{max} (μg/mL)	AUC (μg·h/mL)	t1/2 (h)	t_{max} (h)	Cl (L/h/kg)	Vdss (L/kg)	% F_{abs}
	Single + NVP bid + 2 N(t)RTIs			16?	400 TMC207 + 200 NVP		NR	44.69± 14.07	9512±34 79	NR	12.0 (8.0-168)	NR	NR	NR

NR=Not reported in the Investigator's Brochure
^Intermittent dosing (twice weekly)
^^ AUC determined over 168 hours and divided by 7
a=dose reduced from 40 to 20mg/kg on Day 115/114
b=dose reduced from 40 to 20 mg/kg at 2 months
