# Supplementary material for: Mycobactericidal activity of bedaquiline plus rifabutin or rifampin in ex vivo whole blood cultures of healthy volunteers: A randomized controlled trial
Source: PLoS One. 2018 May 2;13(5):e0196756. doi: 10.1371/journal.pone.0196756 (PMC5931679; doi:10.1371/journal.pone.0196756)
Supplement: S5 File — (PDF) [file pone.0196756.s005.pdf]

WBA MGIT PROTOCOL

4/15/2018 1:14 PM

ROBERT S. WALLIS, MD, FIDSA

TABLE OF CONTENTS

1. Method description and protocol 3
1.1. Purpose 3
1.2. Scope 3
1.3. Principle 3
1.4. Safety 4
1.5. Equipment 5
1.6. Materials 6
1.7. MGIT TUBE preparation 7
1.8. Mycobacterial stock preparation (standard method) 7
1.9. Stock titration (standard method) 9
1.10. Stock preparation and titration (streamlined method) 10
1.11. Whole blood culture initiation 10
1.12. Whole blood culture harvesting 12
1.13. Data entry 12
2. Spreadsheet installation and instructions 13
2.1. Purpose 13
2.2. Scope 13
2.3. Principle 13
2.4. Requirements 13
2.5. Loading the Solver Add-in 13
2.6. Spreadsheet SETUp 14
2.7. Spreadsheet structure 14
2.8. Macros overview 15
2.9. Titrations page 16
2.10. Curves page 18
2.11. Main page 19
2.12. Generating a report and exporting results 22
2.13. Saving the spreadsheet file 22
2.14. Calculating cumulative effects of drug treatment 22
3. Recommended method summary for publications 23
4. Revision History 23
4.1. Whole blood method 23
4.2. Spreadsheet 24
5. My contact information 25

1. METHOD DESCRIPTION AND PROTOCOL

1.1. Purpose

This protocol describes the materials and method for measurement of mycobactericidal activity in whole blood culture using time to positive in MGIT as the readout for mycobacterial viability.

1.2. Scope

This protocol is to be used in early development of anti-TB drugs or vaccines. It may require modification for a specific study.

1.3. Principle

Mycobacteria added to heparinized blood rapidly undergo phagocytosis and thus become subject to cellular immune mechanisms and the effect of drugs with intracellular accumulation. Overall assessment of these antimycobacterial mechanisms can be accomplished by comparing mycobacterial viability (CFU) of completed culture to that of the inoculum. In this method, time to positivity (TTP) in mycobacterial growth indicator tubes (MGIT) is used instead of conventional colony counts as the readout. This has the advantage of greater sensitivity and reproducibility as well as reduced technician time and exposure hazard.

MGIT tubes contain Middlebrook 7H9 liquid media plus an oxygen-quenched fluorochrome (tris 4, 7-diphenyl-1, 10-phenonthroline ruthenium chloride pentahydrate) embedded in silicone at the bottom of the tube. During bacterial growth within the tube, the free oxygen is utilized and is replaced with carbon dioxide. With depletion of free oxygen, the fluorochrome is no longer inhibited, resulting in fluorescence within the MGIT tube when visualized under UV light. The intensity of fluorescence is directly proportional to the extent of oxygen depletion. MGIT tubes placed in a MGIT 960 instrument are incubated and monitored for increasing fluorescence every 60 minutes. The instrument declares a culture positive if either it reaches a predetermined level of fluorescence or if it shows a rapid rate of increase in fluorescence. Cultures of *M. tuberculosis* generally have approximately 10^5 – 10^6 colony-forming units (CFU) per ml of medium at the time they are declared positive, although cultures with large inocula may be declared positive earlier. The instrument declares a tube negative if it remains non-fluorescent for six weeks (42 days). The detection of growth can also be visually observed by the presence of a non-homogeneous light turbidity or small granular/flaky appearance in the medium. Growth of some NTM (most commonly rapid growers) results in light turbidity, while contaminating bacteria generally produce heavy turbidity.

MGIT TTP is inversely proportional to the log of the inoculum size – the larger the number of inoculating mycobacteria, the shorter the TTP. This relationship is utilized to quantify the change in mycobacterial viability during whole blood culture. If there was neither mycobacterial growth nor killing during the period of whole blood culture, the TTP of the bacteria recovered at the completion of the whole blood culture would exactly equal that of the inoculum. The difference between the two in TTP therefore reflects the change in the number of viable bacilli. A titration curve is used to make this result quantitative. There are two options regarding the preparation of this titration curve.

1. **Standard method.** In this method, a relatively large volume of stock is prepared in advance, and a full titration curve is tested to determine the relationship between log inoculum volume and log CFU, as well as the specific volume to be used in each whole blood culture. Then, when each aliquot is thawed, a viability control culture is performed, in which the same inoculum volume used in whole blood culture goes directly into MGIT. The TTP of this control is then compared to that of the completed whole blood culture to determine change in viability. This method is best suited to an experiment in which a small number of strains (usually 1 or 2) are tested with a large number of subjects.

2. **Streamlined method.** In this method, the titration curve is not determined in advance of the whole blood cultures. Instead, an arbitrary inoculum volume (between 10 to 100 μl) is tested. At the same time, that volume, and at least 1 (or preferably 2) 100-fold dilutions are inoculated directly into MGIT – an "on the fly" titration curve. The TTP of the completed whole blood culture is then compared directly to the abbreviated titration curve to determine change in viability. This method is better suited to experiments in which each subject will be tested using a different strain (usually his or her own). The advantages of this method are savings in time (10-14 days) and cost (fewer MGIT tubes). The disadvantages are that the inoculum volume may not be in a linear portion of the volume-TTP curve, and that unrecognized problems with the bacterial stock (mixed TB and non-TB mycobacteria, for example) may cause the data from that time point to be lost.

Whichever method is used, duplicates are recommended for all cultures. A single batch of stock usually lasts 6 months or more. Aliquots should not be refrozen. Any remaining thawed stock should be discarded, as repeated freezing and thawing causes loss of viability. Viability will also gradually decline during freezer storage (approximately 1 log per year).

TTP (and bacterial density when positive) are affected by the amount of oxygen available in the MGIT tube, *i.e*., the amount of empty "head" space. Less head space will result in shorter TTP and reduced bacterial density when positive. For this reason the total volume of the cultures must be kept constant. There is one exception – the stock cultures – for which slightly more empty space is used, to increase bacterial density and decrease the number of MGIT tubes needed for stock preparation.

1.4. Safety

This protocol shall follow current local and national guidelines as are appropriate for *M. tuberculosis* drug susceptibility testing (DST). Historically, the US CDC has recommended a Biosafety Level (BSL) 2 laboratory with negative air pressure and with an appropriate ventilation system for mycobacterial work, including primary culture and DST. More recently, the CDC has recommended that work involving manipulation of TB cultures, such as DST, be done in a BSL 3 laboratory. However, this work may be done in a BSL 2 laboratory providing the exhaust air from the laboratory is discharged to the outdoors, the ventilation is balanced to provide directional airflow into the room, access to the room is restricted when work is in progress, and the practices and equipment recommended for BSL-3 are followed. This includes use of proper protective gowns, gloves and respirator masks (approved by OSHA or other regulatory bodies) while handling specimens and mycobacterial cultures. International Safety Standards, along with the local specifications, may also be followed. Use an appropriate mycobacterial disinfectant such as Amphyl® for cleaning the work area. The CDC states: "With so many disinfectants available, it is

important to consult the product brochures to make certain the disinfectant is bactericidal for mycobacteria.”

The caps of MGIT tubes must be tightly sealed and the external surface of the tubes decontaminated with an appropriate disinfectant prior to removing the tubes from the biohazard facility. A minimum period of time for decontamination after applying the disinfectant may be specified. Any outside transport of tubes (e.g., to another facility) must be done in an approved container system, usually involving double containment.

1.5. Equipment

1.5.1. MGIT 960 instrument and printer

1.5.2. Table top high-speed microcentrifuge with rotor for 2 ml screw top tubes
Note: Be sure that the rotor can accommodate 2 ml tubes. Do not use smaller tubes. The extra volume is required to provide oxygen for blood cells.

1.5.3. Adjustable calibrated pipetting device

1.5.4. Waste container with bleach or other appropriate disinfectant

1.5.5. Tube racks for 50 ml conical tubes, 2 ml screw top tubes, and MGIT tubes

1.5.6. Incubator 37°C. Added CO_2 is not needed.

1.5.7. Freezer -70°C

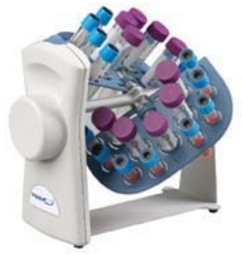

1.5.8. Tube rotators e.g., (3) VWR 13916-822. The rotator provides end-over-end mixing (once every few seconds). Each rotator comes with a specimen plate that has a capacity of 36 2ml tubes. Two rotators may be needed to handle the expected specimen load from a study. The rotators are placed in the incubator. The power cord for the rotator can exit the incubator between the door and the gasket if needed. Tape or other sealing material can be used to limit heat loss through the small gap that results.
Note: A third rotator should be used to hold the blood specimens if they are to be sent in batches to the lab. They should not be allowed to rest on a benchtop, but must be kept in motion, to maintain viability of neutrophils. For this rotator, an accessory rotisserie plate (VWR 13916-832) is needed. This plate has 13 and 16 mm diameter holes to fit vacutainer tubes (2-6 ml and 10 ml, respectively).

1.5.9. Computer with Office 2013 (minimum) or 2016 (preferred), and WBA software version 16 (preferred)

1.6. Materials

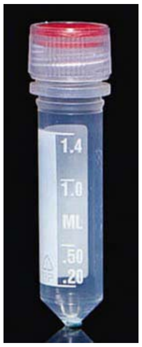

1.6.1. Sterile screw top freezer tubes with O ring seals, 2 ml total capacity (Fisherbrand 05-669-43, Axygen SCT-200-C-S, VWR 10011-600 or equivalent).
Note: Do not substitute tubes of smaller capacity. The extra volume provides oxygen for the duration of the whole blood culture. Do not use snap-top tubes, as they do not seal reliably.

1.6.2. Sterile 50 ml conical bottom tubes

1.6.3. Sterile distilled water without preservatives

1.6.4. RPMI 1640 tissue culture medium with l-glutamine and 20 mM HEPES (Invitrogen or equivalent). Do not use powdered medium to which water must be added. Do not use medium with penicillin, streptomycin or gentamicin!

1.6.5. MGIT 960 automated tubes (containing 7.0 ml 7H9 broth base)

1.6.6. BBL MGIT OADC growth supplement (liquid)
MGIT OADC growth supplement contains 15 ml of the following formula:

Bovine Albumin	50 gm
Dextrose	20 gm
Catalase	0.03 gm
Oleic Acid	0.1 gm
Polyoxyethyline state (POES)	1.1 gm

1.6.7. MGIT PANTA antibiotic mixture (lyophilized)
Each vial of MGIT 960 PANTA (for MGIT 960) contains:

Polymyxin B	6000 units
Amphotericin B	600 µg
Nalidixic Acid	2400 µg
Trimethoprim	600 µg
Azlocillin	600 µg

1.6.8. 7H10 agar plates (for CFU determination of stock)

1.6.9. Sterile disposable plastic bulb pipettes

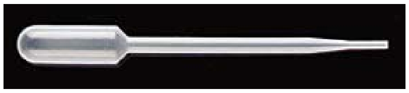

1.6.10. 5 ml syringes with Leur-lok tip, and disk filters for Leur-lok syringes, 0.45 and 0.2 micron
Note: the filters are necessary ONLY if the protocol requires that the supernatants of completed whole blood cultures be saved for subsequent analysis of drug or cytokine levels.

1.6.11. Blood or chocolate agar plates (to check for bacterial contamination)

1.6.12. Note regarding pipetting!

Good laboratory technique requires selecting the appropriate size and shape of container for a particular purpose. Inadvertent touching of the inside wall of a tall, narrow tube with the non-sterile shaft of an adjustable pipette will result in bacterial contamination. One advantage of 50 ml conical tubes is that one can easily reach to the bottom of the tube with the pipette tip without accidentally touching the tube wall. When you must remove the contents of a tall narrow tube (such as a MGIT tube) it is best to do so using a fully sterile disposable plastic bulb pipette. Transfer the contents to a 50 ml tube, from which precise volumes can be measured without introducing contamination.

1.7. MGIT TUBE preparation

1.7.1. Reconstitute one bottle of lyophilized MGIT PANTA with 15.0 ml (one bottle) MGIT growth supplement. Mix until completely dissolved.

1.7.2. Add 0.8 ml of this enrichment to each MGIT tube. Recap the tube(s).
Note: MGIT growth supplement is a sterile product. Handle aseptically. Do not use if turbid or if it appears to be contaminated. Do not leave MGIT tube caps open after adding PANTA/ enrichment. It is important to add the growth supplement in the biological safety cabinet to avoid contaminating the medium. The enrichment with reconstituted PANTA should be added to the MGIT medium prior to inoculation of specimen in MGIT tube. Do not add PANTA/ enrichment after the inoculation of specimen. Do not store MGIT tube after the addition of PANTA/enrichment. It must be used that day or discarded.

1.8. Mycobacterial stock preparation (standard method)

1.8.1. This is best suited to studies in which many subjects will be tested using 1 or 2 strains. Prepare 6 MGIT tubes for stock preparation for each strain by adding PANTA/enrichment (see above)
Note: 7H9 broth may be obtained in bulk from BD. Alternatively, one MGIT tube may be sacrificed to obtain the small volumes needed for this procedure. I recommend using 7H9 with PANTA/enrichment supplement.

1.8.2. Add the appropriate volume of stock. The simplest and most reliable way to prepare stock is to use a previous batch as a starter. If a previous batch is available, add half the volume specified for 5.5 days TTP for that strain (*eg*, if you had previously been using 20 μl in each whole blood culture, use 10 μl to inoculate each new MGIT tube). Add additional 7H9 medium plus PANTA/enrichment to each MGIT tube to bring the total added volume to 400 μl (*eg*, 10 μl old stock plus 390 μl 7H9). Proceed to instruction 1.8.4.

1.8.3. If no previously titered stock is available, picking the correct inoculum volume takes a bit of guesswork. Stocks growth in broth culture other than MGIT often are grown to higher bacterial density. If you estimate the seed culture has 10^7 CFU/ml, prepare a 1:200 dilution of strain seed stock by mixing 20 μl seed culture with 4.0 ml 7H9 broth with PANTA/enrichment in a 50 ml tube. If you estimate other CFU counts, adjust the dilution factor accordingly. Cap and vortex thoroughly. Bacterial clumps often form in cultures grown to high density. Allow clumped bacteria to settle for 10 minutes. Carefully transfer approximately 3 ml to a new 50 ml tube without disturbing the sediment. Add 400 μl diluted stock to each MGIT tube.
Note: The most common error in making new stock is starting with too large an inoculum. If you need stock quickly, it may be best to also prepare a further 1:100 dilution, and set up a set of 5 MGIT tubes for each. Use the batch positive in 6-16 days, and discard the other. Otherwise you may have to wait an extra 1-2 weeks while you sort things out.
Note: The total volume added to the MGIT tubes (400 μl) is reduced from 500 μl to allow for more head space. This will result in somewhat greater mycobacterial growth before the cultures are read as positive, and therefore smaller inoculum volumes.

1.8.4. Place tubes in MGIT 960 instrument. Remove them 3 days after they are recorded as positive.
Note: Stock cultures must become positive between 6-16 days after inoculation. If the cultures are not positive within this time frame, the process of stock preparation should be repeated with more or less dilute stock. Cultures that are inoculated with too large an inoculum and that are positive too soon will contain too few mycobacteria. This is because the algorithm used in the 960 instrument to detect growth includes the rate at which oxygen is consumed as well as its absolute level.
Note: All the tubes may not turn positive simultaneously. In this case, pick a time of harvesting such that all tubes fall within the 1-2 post positivity range. If the variation in TTP among the tubes is greater than 3 days, the stock preparation must be repeated.

1.8.5. Mix the MGIT tubes by inversion. Allow clumps to settle for 10 minutes. Carefully transfer all but the bottom 0.5 ml (approximately) into a single 50 ml tube using a disposable pipette. Cap and mix thoroughly by vortexing. Aliquot into screw top freezer tubes with O ring seals. Save as 1-1.2 ml aliquot and many 100 and 200 μl aliquots. Freeze at -70°C.
Note: I have had 3 instances so far in which Mtb or BCG stock that had been kept in the freezer for extended periods (usually 1 year or more) failed to grow at the expected rate (*ie*, it grew more slowly than expected). This was apparent because the inoculum volume required for the necessary TTP value (5.5 or 6.5 days) was too large (usually more than 60 μl). This did not necessary alter results of the assay, but it does mean that the mycobacterial strain has altered growth characteristics, and therefore

might have altered killing characteristics. If this does happen, the best solution is to reorder stock from ATCC. To prevent this from happening, I recommend thawing, passaging, and refreezing 1 tube of stock in MGIT every 4 months.

1.9. Stock titration (standard method)

1.9.1. Prepare 22 MGIT tubes by adding PANTA/enrichment.
Note: This scheme will have 2 MGIT tubes inoculated with 500 μl, and 4 MGIT tubes inoculated with each of the smaller volumes, down to 0.005 μl. Having 4 replicates improves the accuracy, especially at low inoculum volumes. Having only 2 replicates at 500 μl saves 1 ml of stock.

1.9.2. Thaw one 1.4 ml aliquot of stock. Mix thoroughly by vortexing.

1.9.3. Prepare 5 serial 10-fold dilutions of stock by adding 2.16 ml 7H9 broth (with added PANTA/enrichment) to each of 5 empty dilution tubes. Label these as “50”, “5”, “0.5”, “0.05” and “0.005”.

1.9.4. Inoculate 2 MGIT tubes directly by adding 500 μl of undiluted stock culture. Mix, seal, and label as “500”.

1.9.5. Add 240 μl undiluted stock to the first dilution tube. Cap, mix thoroughly, uncap, and remove 240 μl to the next tube. Repeat until all 5 dilutions are prepared.

1.9.6. Inoculate 4 MGIT tubes each for each dilution by adding 500 μl of each diluted stock. Seal and label appropriately.

1.9.7. Spot 10 ml of undiluted stock onto 3 spots of a blood or chocolate agar plate. Cover and place in incubator. Inspect for bacterial contamination after 3-5 days. Discard stock if there is evidence of contamination.

1.9.8. Divide two 7H10 agar plate into 3 sectors each. Spot 10 μl from each dilution onto 3 spots per section. Seal plate with parafilm. Place in incubator. Count colonies after approximately 4 weeks. Enter counts from spots with >4 and <200 colonies in the appropriate row in spreadsheet (on Standards page).

1.9.9. Discard unused thawed stock. Decontaminate the external surfaces of the MGIT tubes. Place MGIT tubes in MGIT 960 instrument. Note TTP, and enter values in spreadsheet.

1.9.10. The inoculum volume for whole blood cultures will be selected as that volume calculated to be positive in 5.5 days.
Note: See section 2.9.2. This value may be decreased (*i.e.*, inoculum volume increased) for trials of highly bactericidal drug combinations. This volume may be

decreased (*i.e.*, longer TTP) for vaccine trials. Stock must have been frozen prior to titration to account for a small loss of viability due to freezing. In addition, stock will slowly decline in viability during further storage, usually amounting to 0.5 log in 6 months. This will be apparent in the spreadsheet (see spreadsheet instructions). New stock preparation to account for loss in viability during storage should be anticipated and fresh stock prepared periodically for long studies.
Note: The titration curve for each batch of stock must be approved by Dr. Wallis prior to use in whole blood culture experiments.

1.10. Stock preparation and titration (streamlined method)

1.10.1. Any MGIT culture that has been reported as positive may be used. The culture should have been allowed to continue incubation for 1-2 additional days after being identified as positive. It may have been previously frozen, or may be used directly from the 960 instrument.

1.10.2. An arbitrary inoculum volume is used for whole blood culture. As a general rule 20 μl is recommended.

1.10.3. MGIT tubes for the titration curve are prepared simultaneously with setting up the whole blood cultures. A total of 3 pairs of tubes are required if exponential regression is to be used. Exponential regression usually produces a more accurate fit. One pair of tubes should contain the arbitrary whole blood inoculum volume (plus sufficient supplemented 7H9 broth for a total added volume of 500 μl). The other 2 pairs of tubes should contain 1:100 and 1:10000 dilutions of the first. For example, if 20 μl is used for whole blood culture, the titration curve tubes should be inoculated with 20 μl of neat, 1:100, and 1:10000 dilutions. These will be recorded in the spreadsheet as 20, 0.2 and 0.002 μl.

1.10.4. If linear regression is substituted, two data points are sufficient. In this case, neat and 1:100 are used.

1.11. Whole blood culture initiation

1.11.1. Blood is collected using sterile sodium heparin vacutainers. The top of the vacutainer should be cleaned with an alcohol wipe prior to filling or emptying. Tubes may be kept at room temperature for up to 16 hrs to facilitate shipping batches from the clinical site to a lab. However, the blood tubes must be either be in same type of rotator used in the incubators, or kept horizontal on a rocking platform.

1.11.2. Label 2-2.0 ml screw top tubes for each blood specimen (18 blood specimens, 36 tubes). Label according to time post-dose and subject.
Note: 2 cultures per specimen is sufficient when a set of several time points are

studied for each patient. However, if only a single blood draw is being collected, 4 cultures should be performed per sample.

1.11.3. Add PANTA/enrichment to 4 MGIT tubes. Label as to date.

1.11.4. Examine the spreadsheet to determine the appropriate inoculum volume. This volume is calculated on the Standards sheet and is carried over to the Main sheet of the workbook. Thaw sufficient stock for the full day's experiments. Combine the contents of the thawed tubes and mix well. Add the specified volume to each of 4 MGIT tubes. Add additional MGIT medium to bring the total added volume to 500 μl (*eg*, 20 μl stock plus 480 μl supplemented 7H9 medium). Cap tightly, mix, decontaminate external surface and place in MGIT 960 instrument. These are direct-to-MGIT controls to assess stock viability.
Note: In some cases, the desired inoculum volume may be too small to pipette directly (0.5 to 1.5 μl). In this case, remove 10 μl of stock, add 90 μl of RPMI 1640, and use 10 times the indicated volume (*eg*, 5 to 15 μl). In this case, see note 12.6, centrifugation to remove OADC is not needed.

1.11.5. Add the same volume of stock to each of 2-2.0 ml screw top tubes with O rings. Sediment mycobacteria in centrifuge at 12000 RPM for 10 min.

1.11.6. Remove tubes from centrifuge. Remove liquid by pipetting (leave no more than 10 μl remaining).
Note: MGIT medium is removed to avoid adding 7H9 plus OADC to mammalian cell culture. For small inoculum volumes (2 μl or less, or 20 μl or less of 10-fold diluted stock) this is not necessary. In these cases, add the 300 μl RPMI first (step 12.7), add the small inoculum directly, and then add blood.

1.11.7. Add 300 μl RPMI with l glutamine and HEPES to each tube. Seal tubes. Resuspend bacteria by vortexing.

1.11.8. Add 300 μl heparinized blood to each tube. Seal tube. Mix gently.

1.11.9. Place in rotator in incubator at 37°C for 72 hr.
Note: The duration of whole blood culture may be shortened by up to 4 hrs (*ie*, 68 hrs). This may be done if specimens arrive late in the day, so that harvesting the whole blood cultures can be done without unduly extending the workday. In this case, be sure to enter in the spreadsheet the correct day <u>and time</u> that the whole blood cultures were placed in the incubator and when they were removed. The spreadsheet calculates change in viability per day of whole blood culture, and will adjust for the slight reduction in culture duration.

1.11.10. Discard any remaining thawed stock. Do not refreeze!

1.12. Whole blood culture harvesting

1.12.1. 13.1 Add PANTA/enrichment to 1 MGIT tube for each whole blood culture tube.

1.12.2. Remove whole blood cultures from incubator. Centrifuge at 12000 RPM for 5 min. Remove supernatants (approximately 450 μl) from replicate cultures. Supernatants may be combined and saved, or if not required, may be discarded.
Note: If necessary, supernatants may be sterilized by passing first through 0.45 micron and then 0.2 micron filters. Save and label according to subject and timepoint. Freeze at -70C.

1.12.3. Add 1.0 ml sterile distilled water. Vortex. Repeat vortexing after 5 and 10 min at room temperature. This step will lyse most RBCs.

1.12.4. Centrifuge at 12000 RPM for 10 min. Remove and discard liquid. Resuspend pellet in 500 μl supplemented MGIT medium. Vortex thoroughly. Add to a MGIT bottle.
Note: Some completed whole blood cultures will have a small visible clump of platelet fibrin debris at the bottom of the tube. If this material is not fully transferred to the MGIT tube by the first pipette transfer, remove a small volume of medium from the MGIT tube, resuspend the pellet, and again attempt to transfer this clump of material to the MGIT tube.

1.12.5. Decontaminate external surface of the MGIT tube and place in MGIT 960 instrument. Record the TTP when positive.
Note: MGIT bottles should be visually inspected when they are positive. A small percentage of cultures (generally much less than 1%) may be bacterially contaminated. This will be apparent as very short TTP (<2 days) and heavy visible turbidity. Data from these cultures cannot be used and should be reported as contaminated. Suspected bacterial contamination may be confirmed by gram and AFB staining if necessary. All used MGIT bottles must be autoclaved prior to being discarded.

1.13. Data entry

The use of pre-printed bar code labels is highly recommended. For Pfizer studies, extra bar code labels are provided along with blood samples. Use these to label the whole blood and MGIT tubes.

Each day as tubes turn positive, print out a hard copy from the MGIT 960. Affix the barcode specimen ID labels. Save the hard copy. Enter TTP data in the spreadsheet. Add your initials to the rows of data.

Each week, present the spreadsheet and hardcopy to the laboratory supervisor for QC. The supervisor will have been provided the supervisor passcode for WBA software, and will lock the cells or rows that are confirmed by the hard copy.

2. SPREADSHEET INSTALLATION AND INSTRUCTIONS

2.1. Purpose

This document describes the installation and use of the Excel spreadsheet WBA v16 for measurement of antimycobacterial activity in whole blood culture using MGIT as the readout for mycobacterial viability.

2.2. Scope

This spreadsheet is to be used for studies of TB drug activity, vaccines, and immunomodulators, added directly to whole blood cultures or after oral or parenteral administration.

2.3. Principle

The MGIT 960 system uses culture tubes containing an oxygen-quenched fluorochrome embedded in silicone at the bottom of the MGIT tube. The system continuously monitors tube fluorescence to detect bacterial growth. MGIT time to positivity (TTP) is inversely proportional to the log size of the inoculum. Δ log CFU (change in viability during whole blood culture) therefore can be calculated as log(*final*) – log(*initial*), where *initial* and *final* are the volumes of stock culture corresponding to TTP values of the inoculum and the completed whole blood culture, respectively. This software will assist you in quantitative use of MGIT by:

- Facilitating data entry by detecting and preventing common errors
- Creating a standard curve that relates inoculum volume to TTP for each batch of stock
- Determining the appropriate inoculum volume for that stock based on the desired TTP, using point-to-point interpolation of the growth curve
- Calculating the log change in viability (growth or killing) during whole blood culture based on the difference in TTP of the inoculum and the completed whole blood culture
- Monitoring the viability of the stored stock culture over time
- Generating standardized reports

2.4. Requirements

2.4.1. Excel 2013 (minimum) or 2016 (preferred)

2.4.2. MGIT 320 or 960 instrument and printer

2.4.3. WBA workbook version 16

2.4.4. Solver Excel add-in
Note: This add-in is included in most versions of Office. For Office versions on the Mac, Solver is available as a free download at www.solver.com

2.5. Loading the Solver Add-in

2.5.1. Start Excel. Go to the Office button in the upper left corner of the Excel window. From

there, go to Excel Options at the bottom, then Add-ins, then Manage Add-ins, and select Go.

2.5.2. Check the box for the Solver Add-in.

2.5.3. The add-in will then be loaded automatically each time Excel is started.

2.6. Spreadsheet setup

2.6.1. Open a blank version of the WBA workbook.
Note: the file extension is xlsb, not xls. The file is password protected. I will provide you with the password. You will also be asked for your initials. These will be used to sign rows of entered data.

2.6.2. You must enable macros for this workbook. How you do this will depend on the setup of Excel and Windows. Usually a Security Warning bar will appear below the toolbar, with a button to press for Options. Select “enable macro content.”

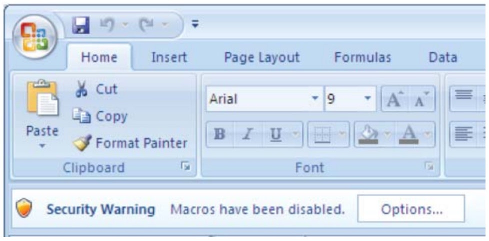

2.6.3. Save the workbook using a different name, such as NAC study WBA 2Feb18.
Note: Save the workbook every week using a different filename that includes the save date. This way you can go back to previous files in the event of problems.
Note: The spreadsheet uses the xlsb (binary) format. I recommend <u>always</u> saving in this format, as it is smaller, loads faster, and permits much larger spreadsheets.

2.7. Spreadsheet structure

The workbook has several sheets. The 3 you will use most often are Titrations, Main, and Report. The Curves page lets you inspect the curve fitting. The Temp and Imported sheets are used to bring data regarding specimens into the workbook. If you export data as a comma-delimited CSV file, a new page containing that data will also be created.

There are macros that can be called from the Standards and Main sheets, using a combination of <ctrl> plus another key. These keys are listed in blue in row 1 of the Standards and Main pages.

You will notice that on the Standards and Main sheets, many rows and columns are hidden from view. These cells contain formulas that are used to populate the workbook. They are hidden and locked so they cannot inadvertently be changed.

- Cells with a white background are blank. You can enter data in these cells as needed.

- Cells with a cyan background cannot be changed.
- Cells with a light cyan background have a recommended value. The values in these cells are not locked, and may be changed. Such cells are present on the Titrations and Main sheets.

2.8. Macros overview

2.8.1. **<ctrl> a adds rows** to the bottom of a spreadsheet. On the Standards page, it will add 6 rows for one additional standard curve. On the Main page, it will add 10 rows. It does not matter where the cursor is located when you do this – the rows will always be added after the last formatted row.

2.8.2. **<ctrl> u underlines** the selected row. Use this to separate each day's experiments.

2.8.3. **<ctrl> n** removes underlines.

2.8.4. **<ctrl> p toggles supervisor mode**. A password will be required (again, I will provide this to you). A red highlighted box indicating this mode will appear in the upper right of the screen. Supervisor mode is required to QC and lock entries. <ctrl> s again will turn off supervisor mode.

2.8.5. **<ctrl> s signs** the selected row(s) with your initials.

2.8.6. **<ctrl> q QC locks** the selected entries. Wait until the entire row of data is complete. Select the rows you wish to lock, then enter <ctrl> q. Neither users nor supervisors can undo QC locking. If you have inadvertently locked the wrong cells, send the spreadsheet to me with a note of explanation and I will repair it.

2.8.7. **<ctrl> r creates a new report** page. If there is a previous report, it will be erased and updated. Two report formats are possible: standard or export. Reports can be rewritten at any time. A full section appears below regarding reports and exported CSV files.

2.8.8. **<ctrl> v pastes values** (see below).
Note: Occasionally you may enter a value in the wrong cell. Resist the temptation to drag or to cut and paste! This may cause problems if another cell is looking at the value you just entered. Cutting and pasting may move the reference to the new cell. You do not want to do this! Instead, copy, move to the correct cell, and use **<ctrl> v** to paste the value, and delete the incorrect entry. Or just re-enter the number in the right place, and delete the erroneous entry.

2.8.9. **<ctrl> f fits** a regression curve to the titration data (see below).

2.9. Titrations page

	A	B	C	D	E	F	G	H	I	J	M	N	O	P	Q	R
1	<ctrl> a=add_rows u=underline n=no_underline s=supervise q=QC_lock r=report v=pasteValues f=fit											Default TTP target: 5.5				
2	WBA v13.1 ©RS Wallis	TTP raw data				Colony counts (10 ul)			Inoculum	CFU/ml	TTP	log Inoculum		Parameter	Parameter	QC
3	Isolate & date	days	hrs	days	hrs	cols	cols	cols	ul		days	actual	predicted		Value	date
10	H37Rv 03-Mar-10	3	2	3	4	80	90	80	500	8.33E+03	3.13	2.70	2.79	Volume (ul)	13.32	11-Jun-13
11	H37Rv	4	18	4	16	40	31	34	50	3.50E+04	4.71	1.70	1.58	y0	0.000	11-Jun-13
12	3-Mar-10	6	12	7	3	15	11	9	5	1.17E+05	6.81	0.70	0.49	a	8.049	11-Jun-13
13	5.5	7	21	8	12	2	2	0	0.5		8.19	-0.30	-0.05	b	0.339	11-Jun-13
14	exponential	10	21	14	8	1	0	0	0.05		12.60	-1.30	-1.32	Σ residual	0.128	11-Jun-13
15						0	0	0	0.005			-2.30		log CFU/ml	4.73	11-Jun-13
16	H37Rv 03-Mar-11	3	15	3	17				500		3.67	2.70	2.67	Volume (ul)	27.04	
17	H37Rv	4	23	4	21				50		4.92	1.70	1.77	y0	0.000	
18	3-Mar-11	6	11	7	8				5		6.90	0.70	0.74	a	8.793	
19	5.5	10	1	10	5				0.5		10.13	-0.30	-0.43	b	0.328	
20	exponential	14	9	13	3				0.05		13.75	-1.30	-1.36	Σ residual	0.040	
21		17	7	18	20				0.005		18.06	-2.30	-2.20	log CFU/ml		
22									500			2.70		Volume (ul)		
23									50			1.70		y0	0.000	
24									5			0.70		a	10.000	
25	5.5								0.5			-0.30		b	1.000	
26	exponential								0.05			-1.30		Σ residual		
27									0.005			-2.30		log CFU/ml		
28									500			2.70		Volume (ul)		
29									50			1.70		y0	0.000	
30									5			0.70		a	10.000	
31	5.5								0.5			-0.30		b	1.000	
32	exponential								0.05			-1.30		Σ residual		
33									0.005			-2.30		log CFU/ml		
34									500			2.70		Volume (ul)		
35									50			1.70		y0	0.000	
36									5			0.70		a	10.000	
37	5.5								0.5			-0.30		b	1.000	
38	exponential								0.05			-1.30		Σ residual		
39									0.005			-2.30		log CFU/ml		

Titrations Curves Main Report

2.9.1. Strain and date

Enter the strain name and the start date for the MGIT titration in column A. Each strain and batch must have a unique name, which will consist of these two entries strung together. The correct date must be entered in dd-mmm-yy format using the English 3 letter month abbreviation.

2.9.2. Target TTP value

The default target TTP for all existing and new batches of stock appears in cell P1. I have used 5.5 days for drug studies, 6.5 for vaccine studies. You may change this value for a new spreadsheet (and a new series of experiments). However, do not change this value midway through an experiment, as the change will cascade to other pages in the spreadsheet. You may change the target TTP for an individual batch of stock later on if you need to do so. However, here again, do not change this value after you have started using that batch of stock in specific experiments, as this change will also cascade to other pages in the spreadsheet. It is desirable for the TTPs of the inoculum and the completed cultures to be in the mid-portion of the curve.

2.9.3. Regression type

The next cell down lets you select one of two regression types: exponential (the default) or linear. The exponential regression equation is as follows:

$$y = y0 + ae^{-bx}$$

where y = TTP in days, and x = log inoculum volume in μl. Solving this equation for x yields:

$$x = -\ln\left(\frac{y - y0}{a}\right)/b$$

This equation is used to calculate the apparent inoculum volume of each culture based on its TTP.

Linear regression is an alternative option. It is generally less accurate and not recommended except in circumstances when only 2 data points are available, or when exponential curve fitting is not successful. The equation is as follows:

$$y = y0 - ax$$

Solving for x yields:

$$x = -(y - y0)/a$$

Negative values (-*ax*) are used so that the values of *y0* and *a* are similar whether one selects exponential or linear regression. This helps avoid some error messages in the Solver module when changing from one regression type to the other.
NB: The cells for *y0, a,* and *b* are not protected and do not contain formulas. You may enter values as you wish to see the effect on the regression curve. The default values are 0, 10, and 1, respectively. If there is a specific batch of stock for which the curve fitting is not successful, send me the file and I will see what I can do.

2.9.4. TTP entry

TTP values of replicates (in days and hrs) are entered in columns B to E. Columns may be left blank if only single values are used in a particular study. Rows may be left blank if the streamlined method is used. In this case, I suggest using the bottom 2 or 3 rows in the block for data entry. Change the inoculum volumes in these rows to match those used. This will help avoid graph errors on the Curves page (see below). Note: the current spreadsheet has columns for TTP values of 4 replicate MGIT cultures.

2.9.5. CFU count entry

Enter the colony counts from the replicate 10 μl spots in the appropriate cells. Spots with <3 or >200 colonies will be ignored. The other entries will used to calculate the average CFU count per ml. This is for information purposes only, but most manuscript reviewers want to know this.

2.9.6. Curve fitting

You must fit the curve to the TTP values of the titration curve before using the stock. This is done on the titration page of the spreadsheet. To calculate the regression parameters, select the cell containing the full name (strain and date), and enter <ctrl> f. Enter yes to continue. Solver uses an iterative process to identify parameters such that the difference between predicted and actual values (in the cell marked Σ

residual) is minimized. After a few moments new results will be displayed. Answer Yes to accept them. NB: The Σ of residual value indicates the goodness of fit, with smaller being better. For exponential curves, this should generally be <0.5. Curves with values greater than this will appear highlighted in red, as the fit is suboptimal. For linear equations, the Σ of the residual values may be as high as 5. If the value is greater than 5, the resulting volume from this equation will not be carried over to the Main page, which will indicate an error message (a red X).

2.9.7. Inoculum volume

The inoculum volume calculated to be positive in desired number of days appears in column Q. This volume will be used to inoculate all whole blood cultures with this batch of stock.

2.9.8. QC lock

QC lock the rows for the batch of stock (see section B13 below). Be sure to lock the rows for each batch of stock before conducting any experiments using that batch!

2.10. Curves page

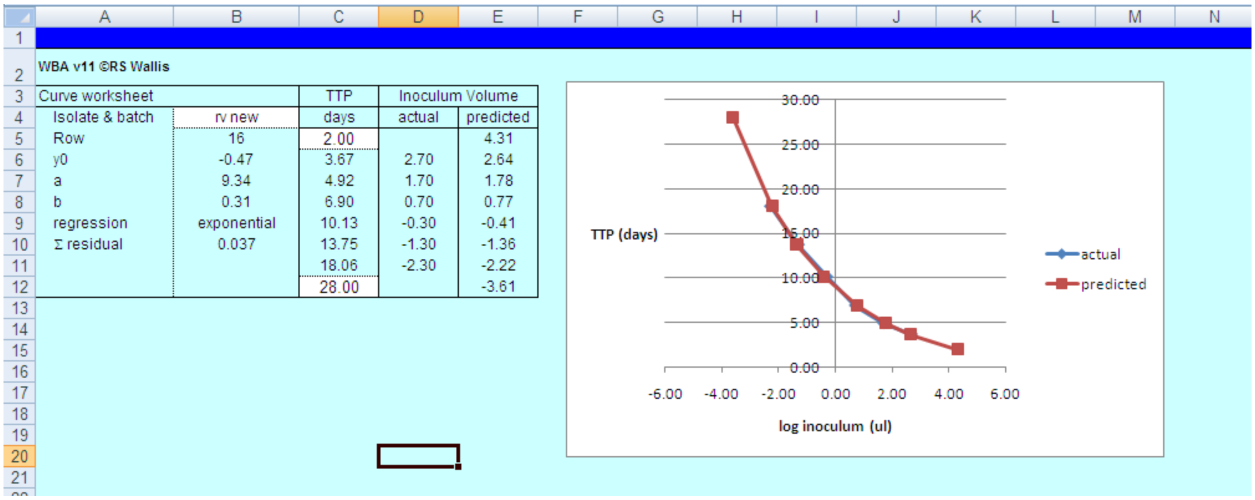

WBA v11 ©RS Wallis

Curve worksheet		TTP	Inoculum Volume	
Isolate & batch	rv new	days	actual	predicted
Row	16	2.00		4.31
y0	-0.47	3.67	2.70	2.64
a	9.34	4.92	1.70	1.78
b	0.31	6.90	0.70	0.77
regression	exponential	10.13	-0.30	-0.41
Σ residual	0.037	13.75	-1.30	-1.36
		18.06	-2.30	-2.22
		28.00		-3.61

This page can help you visually inspect the quality of the regression curve. Enter the name of the batch in column B. The corresponding curves will be displayed. They should be superimposed.

The advantage of curve fitting over point-to-point interpolation (the method in previous WBA spreadsheets) is that predictions can extend somewhat beyond those values actually measured (*i.e.*, results may be extrapolated). This can be useful for unexpectedly active drug combinations that are highly bactericidal. The 2 blank cells in column C can be used to examine how the equation parameters will be used to calculate such outliers.

XY charts in Excel do not gracefully handle missing data. I have tried to include a work around to fix this, but you may see odd looking graphs when fewer than 6 dilutions are tested.

2.11. Main page

	A	B	C	D	E	F	G	H	I	J	K	L	M	N	AB	AC	AD	AE	AF
1	<ctrl> a=add_rows u=underline n=no_underline s=supervise q=QC_lock r=report v=pasteValues f=fit									Dmax= 0.5									
2	Subject	Group	Specimen	Date or	Type	Isolate	Ctrl	MGIT	TTP				Inoc		WB	TTP		WBA	QC
3			barcode	study day	or time	& batch	row	start date	days	hrs	days	hrs	ul		days	mean	stdev	log/d	date
14					ctrl	H37Rv 05-Apr-13		12-May-13	5	14	5	16	168.87	√		5.63	0.06	-0.092	
15	A1				pre	H37Rv 05-Apr-13	14	15-May-13	6	3	6	7	168.87	√	3	6.21	0.12	-0.137	
16																			
17																			
18																			
19																			

2.11.1. General

Estimate how many experimental samples you will be adding to the spreadsheet that day. Add sufficient new rows to the spreadsheet before you enter data. Each <ctrl> a keypress will add 10 rows. (If you mistakenly enter data in an empty row, it will be lost when you add rows). Note: the current spreadsheet has columns for TTP results of 4 replicate cultures.

Mark the beginning of a new day's experiment with an underline. Select the previous row and enter <ctrl> u.

Additional columns have been added in the current spreadsheet version so that 4 replicates can be entered.

2.11.2. Direct-to-MGIT control

If you are using the Standard titration method, start off the entries for that day by entering the direct-to-MGIT controls. You will have one row for each mycobacterial strain thawed that day. Multiple subjects do not require multiple direct-to-MGIT controls – one will suffice, so long as they were all studied on the same day with the same aliquot. However, you should enter 4 replicates for these controls. No SID (subject ID no) or visit are required for these control rows. Select the appropriate strain and date from the pull-down list. The correct inoculum volume will be carried forward from the Standards pate. In column E enter "ctrl" – exactly as written. Leave column G blank, and enter the date the MGIT bottle was placed in the 960 instrument in column H.

2.11.3. Whole blood entries

Use the next rows for that day to enter the whole blood cultures. Enter the subject, group (if needed), batch, and inoculum volume. In column E ("type or time"), enter the time post dose the specimen was collected. (Actually you may enter anything here other than "ctrl". The column cannot be left empty, however.)

The entry for column I ("Ctrl row") depends on the titration method. If you used the Standard titration method, enter the spreadsheet row number of the direct-to-MGIT control for that whole blood culture. The names of the isolates in the two rows must match, otherwise a red x will appear. If you used the Streamlined method, enter 0 (zero) in this column.

Note: The viability of the direct-to-MGIT controls tends to decrease over time during freezer storage. It can also appear to increase or decrease randomly. Viability of the controls is compared to expected viability using the titration curve. Controls that differ more than 0.5 log are not considered acceptable.

Sample rows that refer to controls that are more than 0.5 logs off expected viability will display an x and will not have log change calculated.

Enter the date the MGIT bottles go in the 960 instrument. The spreadsheet will calculate from this the duration (in days) of the whole blood culture. This is used to calculate the log change in viability per day of whole blood culture.

Enter in days and hours the TTP values of the MGIT cultures as they become positive. The spreadsheet will calculate the mean and standard deviation of the replicates.

2.11.4. Standard method

If the Standard titration method is used, the spreadsheet will compare the mean TTP of the direct-to-MGIT control with the same volume on the standard curve for that batch of stock. The difference in inoculum volumes will indicate any decrease in viability of the stock. For these direct-to-MGIT controls, column AE ("WBA") indicates the $\log_{10}$ change in viability of this aliquot compared to the original stock when first frozen. Negative values indicates reduced viability. The value for the control will appear in parentheses. This is to indicate represents simply log change, not log change per day as in all other reported results. The spreadsheet will then compare the TTP of the completed whole blood culture to its direct-to-MGIT control. For these rows, column AE indicates changes in viability per day during whole blood culture. Again, negative values indicate reduced viability. Cell K1 indicates the maximum loss of viability of the stock in the freezer (Dmax). The default value is 0.5 log. If the stock has declined in viability greater than this, results will not be calculated for the samples. The value may be changed, but this is not recommended.

2.11.5. Streamlined method

If the Streamlined titration method is used, the TTP of the completed whole blood culture is compared directly to the titration curve.

Some researchers have expressed interest in examining changes in viability immediately after inoculation. In this case, results are expressed as log change rather than log change per day to eliminate the division by zero error. Results appear in parentheses as they do for controls.

2.11.6. Importing specimen information using the Import macro

Note: This feature is not functional in this version.

In some situations, it may be handy to use data from an external spreadsheet to populate the Main sheet. If your clinical unit can generate this file at the time the specimens are shipped to the lab, this approach can help avoid errors in transcription or copying and pasting. This can be doubly advantageous if this spreadsheet can also be used as the basis of final report, to be uploaded to a database at the completion of a study. There are 2 steps to this process: copying the data from the external file to the Temp sheet, and running the Import macro (<ctrl> i). An example using the Pfizer lab data file specification is as follows.

The Pfizer lab data file specification calls for 23 fields (columns) and 1 row per result. The fields start with study number, lab ID, specimen accession number, SSID, visit, collection date, collection time, test name, etc. The file generated by the New Haven CRU has entries in many but not all these columns (for example, test result, column P, is blank). In addition, the CRU adds 2 more columns not included in the file specification, specimen barcode and simple subject ID. These fields are used in the lab to manage specimens but are not reported back to Pfizer.

Copy all columns in the CRU file (A:Y). Place the cursor in cell a1 of the Temp sheet of the WBA workbook and paste values (<ctrl> s). The Temp sheet has additional columns to the right (AI:AL) that review the date and time of specimen collection and use this to calculate the date on which the lab will receive the specimen. This is necessary because specimen batching may require specimens to be collected on Sunday evening and Monday morning to be batched together and sent to the lab Monday afternoon. Finally, a MGIT start date is calculated, based on 3 days whole blood culture incubation (*i.e.*, 3 days after lab receipt of specimen). These columns may require specific modification for other file specifications and study protocols with different batching procedures.

Press <ctrl> i to initiate importing. Data will first be copied to the Imported sheet, using the first empty row. This sheet is cumulative, so that each import fills subsequent rows. Next specific columns of data will be copied from the Temp sheet to the Main sheet. Additional rows of formulas will be added to the Main sheet as needed to accommodate these specimens. One empty row will be left after the last existing entry to be used for the direct-to-MGIT control cultures. You will be asked for the isolate and batch for these cultures. Be sure to spell this exactly as it appears on the Standards sheet.

Inspect the results. If all is satisfactory, select columns A:Y from the Temp sheet and delete the data. Do not delete the columns – just the contents.

2.11.7. Signing results

You should always sign the data you have entered. Do this by selecting the appropriate rows and entering <ctrl> s.

2.11.8. Supervisor mode and QC locking

Cells that have been locked cannot subsequently be changed. <u>This is highly recommended to ensure data integrity.</u> Locking also removes formulas in cells and replaces them with values. This helps the spreadsheet to run quickly.

Cells can only be locked while in Supervisor mode. Supervisor mode is entered by <ctrl> p. A password will be requested. I will provide this password to you. It differs from the password used to open the file. The word "Supervisor" will then be displayed in red in the upper right of the sheet.

Check the data entry against the MGIT printout to be sure there have been no transcription errors. Select either specific cells in a row to be locked, or one or more entire rows. Enter <ctrl> q. The color of the type in the selection will now appear dark red rather than black. If an entire row has been selected, the column on the far right will now contain the date of locking.

Enter <ctrl> s again to leave Supervisor mode.

2.12. Generating a report and exporting results

Reports are useful to summarize and review results in a study. Unlike the Main sheet, entries on the Report sheet have no formulas, are not protected, and have minimal formatting. You may sort and filter as needed. Reports may be generated many times during a study. Each new report replaces the previous one. Reports may also be used to generate a permanent file for export. This should generally be done only when a study is completed, and is possible only if the Import macro was used to populate the Imported and Main sheets of the workbook.

Generate a report by entering <ctrl> r. Select 1 of the 2 options: Standard or Export. The Standard report has many columns, including Subject, Group, Strain, inoculum volume, time post dose, whole blood culture date and duration, mean and SD days to positive, and WBA. This type of report is useful to review data during a study, or to organize the results to perform calculations (such as AUC) at the conclusion of the study. This type of report includes the direct-to-MGIT controls.

Note: The Export option is not functional in the current version. It first creates an abbreviated report, with only subject, barcode, time post dose, and result. Direct-to-MGIT controls are not included. The data are sorted according to barcode. The macro then similarly sorts the data in the Imported sheet, and inspects each row to ensure that the subject ID and barcodes of the 2 sheets match. If a discrepancy is found, an error message will be returned indicating the row number of the mismatch, and the macro will terminate.

If no discrepancies are found, a new sheet will be created. Relevant columns from the Imported and Report sheets are copied to the new sheet to create a final report. The entire workbook will be saved, and then the final report will be saved in a comma-delimited CSV format. The name for this file will be prompted according to the file specification. Be sure the file is saved in the folder you intend. The sheet will be renamed to match that of the CSV file. You will then be prompted to resave the entire workbook in a new file. See comments below regarding file naming.

2.13. Saving the spreadsheet file

The spreadsheet file contains all the data for the entire project. It is recommended that a copy be saved each week with a file name indicating the project and the date it was saved (*e.g.*, B1171002 15-Dec-2008.xlsb). Remember that slashes ("/") cannot be used in file names, although dashes are fine. I recommend avoiding periods except to separate the .xlsb file name extension.

16.2 Excel may ask you if you want to save this file in a more secure format (.xlsm or xlsx), since it is already encrypted and protected. Answer "NO" to this question. <u>Always</u> save in .xlsb format.

2.14. Calculating cumulative effects of drug treatment

Results are generally represented in 2 formats: discrete timepoints, and as cumulative effect. Cumulative effect is calculated as the integral (AUC) of values at discrete timepoints, using the trapezoid

method. This in essence creates a dynamic time-kill curve (in which drug concentrations change during the dosing interval) from static data. An example is shown in the figure below.

	E	F	G	H	I	J	K	L	M	N
1	Time	Isolate	MGIT start	Inoc	WB days	TTP mean	TTP SD	log/d	cumulative	comment
2	ctrl	H37Rv 13-	18-Nov-16	76.81		5.79	0.06	-0.16857		
3	0.00	H37Rv 13-	21-Nov-16	76.81	3.00	5.15	0.32	0.128568	0.00	starts at zero
4	1.00	H37Rv 13-	21-Nov-16	76.81	3.00	5.79	0.41	0	0.0026785	equals M3+(E4-E3)*AVERAGE(L3,L4)/24
5	2.00	H37Rv 13-	21-Nov-16	76.81	3.00	8.88	0.00	-0.46413	-0.006991	equals M4+(E5-E4)*AVERAGE(L4,L5)/24
6	3.00	H37Rv 13-	21-Nov-16	76.81	3.00	7.54	0.06	-0.2871	-0.022642	etc
7	4.00	H37Rv 13-	21-Nov-16	76.81	3.00	6.77	0.09	-0.16986	-0.032162	
8	6.00	H37Rv 13-	21-Nov-16	76.81	3.00	6.42	0.12	-0.11144	-0.043882	
9	8.00	H37Rv 13-	21-Nov-16	76.81	3.00	5.92	0.12	-0.02322	-0.049493	
10	24.00	H37Rv 13-	21-Nov-16	76.81	3.00	5.79	0.40	0	-0.057233	

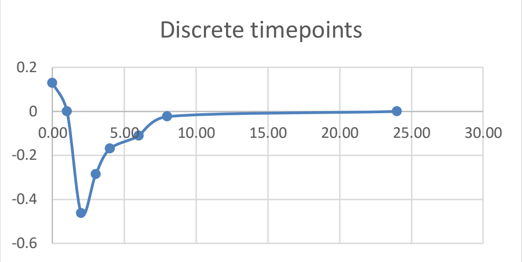

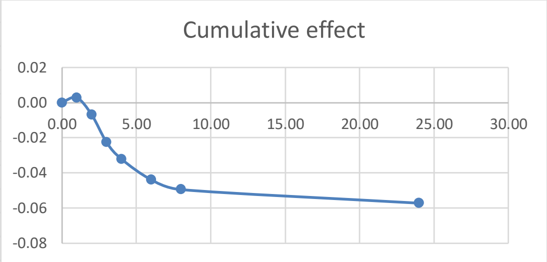

The left figure shows column L (log/d) plotted vs col E (hr), whereas the right figure shows column M. The formulas for the cells in column M are shown in column N. Each cell in column M represents the sum of the cell in the previous row, plus the AUC during the next interval.

3. RECOMMENDED METHOD SUMMARY FOR PUBLICATIONS

For each batch of stock, a titration experiment was conducted to establish the relationship between log inoculum volume and MGIT TTP, using a 3 parameter exponential decay curve. The inoculum volume for each whole blood culture was that volume predicted to have a MGIT TTP of X.X days based on this curve. The log change in viability in each whole blood culture was calculated as log (*final*) – log (*initial*), where *initial* and *final* are the apparent volumes of the inoculum and the completed culture based on their respective TTP values, calculated using the titration curve. Results at discrete time points are reported as log change per day of whole blood culture. The cumulative effect is calculated as the integral (AUC) of values at discrete time points, creating a dynamic time kill curve from static data. The full protocol is available online at rswallis.com.

4. REVISION HISTORY

4.1. Whole blood method

February 26, 2009 - Duration of whole blood culture corrected for TB drug trial.

March 9, 2009 – Updated dosing mg and when Period 4 occurs.

March 10, 2009 – Sterile filtering added, 6 hrs substituted for 8 hr time point.

April 01, 2009 – Updated fed subjects text to state this occurs in cohort 2, not cohort 1.

May 12, 2009 – QA/QC added, including OK required for titration curve. Dilution of stock to accommodate small volumes added. Fax number added.

June 25, 2009 – Rocking platform specified for holding blood specimens for batch shipping. Alcohol wipe of vacutainer tops added.

September 23, 2009 – Filter sterilization of supernatants changed to as needed.

November 9, 2009 – Notes added regarding stock started with too large an inoculum. QC procedure revised.

February 19, 2010 – Clarification added re folder name for addins.

June 4, 2010 – Clarification added prohibiting refreezing of stock, and specifying that contents of multiple aliquots be combined and mixed prior to use.

January 3, 2011 – Requirement added to test each batch of stock for bacterial contamination.

Feb 2, 2011 – Comment added regarding 6.5 day TTP for vaccine trials.

May 9, 2011 – Part number added for rotisserie plate for vacutainer tubes.

May 27, 2011 – Streamlined titration method added.

September 11, 2013 – Recommendation to passage mycobacterial stock every 4 months added.

September 3, 2017 – Increased to 4 replicates for titration curve and direct to MGIT controls.

4.2. Spreadsheet

Revision 8.0 December 10, 2008 First version for MGIT.

Revision 8.1 December 12, 2008 CFU counts added.

Revision 8.2 minor errors corrected

Revision 8.3 June 2009 QC added, inoculum volume carried over from Standards page.

Revision 8.4 June 2009 QC macros modified, inoculum volume format changed, error corrected in handling of DTPs longer than standards.

July 2009 Emphasis added regarding the use of a mixing or rotating platform for batches of blood samples.

Revision 8.5 "Group" column added to spreadsheet. Macro to replace formulas corrected to lock cells.

Revision 8.6 Supervisor function added, with authorization to QC. Control keys revised. Delete rows macro removed. Macros to place and remove underlines added.

Revision 9.0 Import and Export functions added. Password security enhanced. Binary file format (xlsb) adopted. Office version 2007 or later required.

Revision 10.0 Calculation method changed from interpolation to exponential regression. Option for triplicate cultures removed.

Revision 11.0 Linear regression option added. Graph page added. Strain and batch concatenated to create full name. Pull down lists of names added. Limits added to MGIT start dates. Standards sheet renamed to Titrations. Streamlined method (on the fly titration) added.

Revision 11.1 Macro to replace formulas corrected.

Revision 12.0 Macros digitally signed.

Revision 12.1 Limit on difference of ctrls from expected values replaced.

Revision 12.11 Clarification that curve fitting must be done prior to use of new stocks, and must be done from the titrations page.

Revision 12.2 Standard language added to describe calculations in publications. Requirement for English 3 letter month abbreviations specified. Inoculum volume now specified to 2 decimal places.

Revision 13.0 Column widths on the Main page are user selectable. Formulas are now shown. Dmax appears on the Main and can be changed by the user.

Revision 13.1 Pfizer digital signature replaced by personal signature. Default target TTP for new stocks now can be set by user. Red x now appears in a ctrl row if its loss in the freezer exceeds Dmax. Results for ctrls and for cultures harvested on day 0 are now shown in parentheses to indicate they are log change rather than log change per day. Improved procedures for curve fitting.

Revision 15. Columns added to spreadsheet to accommodate up to 4 replicate samples. Column added for technician signature. Method updated for titration curve to increase the number of replicates. Calculation of cumulative WBA described.

Revision 16. User initials are now collected when the spreadsheet is opened. A macro fills this information into selected rows.

5. MY CONTACT INFORMATION

Email: rswallis@gmail.com or rwallis@auruminstitute.org

Phone: +27 (0)74 609 2779 or +1 860 271 6745
